# Stability-Driven Pose Prediction and Ligand Design via Contact Persistence Analysis from Molecular Dynamics Simulations

**DOI:** 10.34133/csbj.0093

**Published:** 2026-05-29

**Authors:** Mochammad Arfin Fardiansyah Nasution, Gert-Jan Bekker, Suyong Re, Kenji Mizuguchi

**Affiliations:** ^1^ Institute for Protein Research, The University of Osaka, Suita, Osaka 565-0871, Japan.; ^2^ Department of Chemistry, Graduate School of Science, The University of Osaka, Toyonaka, Osaka 560-0043, Japan.; ^3^ Artificial Intelligence Center for Health and Biomedical Research, National Institutes of Biomedical Innovation, Health and Nutrition (NIBN), Settsu, Osaka 566-0002, Japan.

## Abstract

Molecular dynamics simulations provide deep insights into protein–ligand interactions, but their high computational cost often limits their application in early-stage drug design. To address this issue, we developed a computational workflow that integrates classical docking, high-temperature molecular dynamics simulations at 400 K, and residue-level contact persistence analysis via the *R*-value metric for a rapid assessment of stable binding poses. By retrospective evaluation using 137 protein–ligand complexes across 10 therapeutically relevant targets with a total of 1.64 ms of aggregate simulation time, we found that 83.9% of top-ranked poses identified by this pipeline exhibited a root-mean-square deviation ≤3.0 Å from experimental structures, indicating structural agreement with experimentally observed binding modes under the evaluation criteria used in this study. Furthermore, the *R*-value, a normalized measure of intermolecular noncovalent contact persistence, showed a correlation with experimental binding affinities (p*K*_d_), ranging from 0.40 to 0.94 across 31 systems involving 4 targets, suggesting an association between interaction persistence and experimental binding affinity within the analyzed dataset. We examined whether functional group-level contact persistence can identify ligand moieties associated with stable interactions, thereby supporting hypothesis-driven ligand modification strategies based on persistent contacts. To illustrate the potential application of the workflow, we generated *in silico* derivatives of TH-Z835 and MRTX1133 as KRAS^G12D^ inhibitors and evaluated them using the *R*-value framework as a case study, where several derivatives showed higher predicted interaction persistence than the parent compounds. These analyses illustrate how the workflow may assist in prioritizing ligand modifications for further investigation in structure-based drug design.

## Highlights

• Across 137 protein–ligand complexes (10 targets; 1.64-ms total simulation), 83.9% of top-ranked poses achieve a root-mean-square deviation ≤3.0 Å relative to experimental structures.
• *R*-values linearly correlate with experimental p*K*_d_ (0.40 to 0.94 across 4 targets), validating the stability–affinity relationship.
• Functional group-level persistence pinpoints unstable moieties for stability-guided lead optimization, demonstrated using KRAS^G12D^ inhibitors as a case study.

## Graphical Abstract

**Figure:**
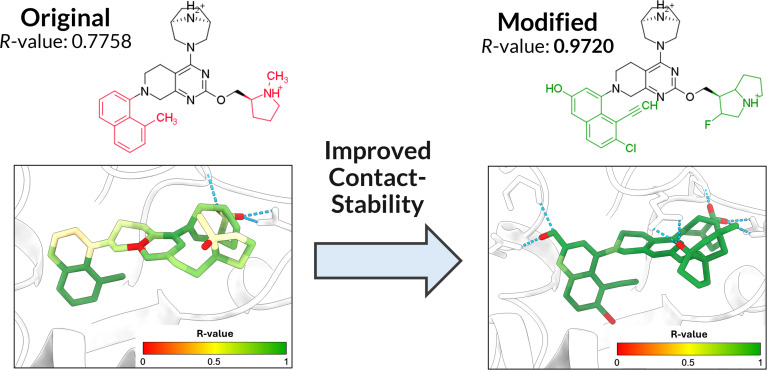

## Introduction

Protein–ligand interactions (PLIs) are essential to fundamental cellular processes such as enzyme catalysis, signal transduction, and receptor activation, making them central to understanding disease mechanisms and designing targeted therapeutics [1]. The accurate characterization of PLIs, which includes not only binding affinity, selectivity, and drug-like properties but also their bound structures, is critical for therapeutic success [2]. While x-ray crystallography provides high-resolution structural information, the resulting structures represent static snapshots typically obtained under cryogenic conditions [3]. Conversely, nuclear magnetic resonance spectroscopy offers dynamic information at physiological temperatures, although high-resolution characterization can be technically demanding [4]. Since these methods are highly demanding in terms of time, cost, and technical complexity, experimental characterization of PLIs remains resource intensive, limiting its throughput in large-scale drug discovery efforts [5].

Recently, artificial intelligence (AI)-based structure prediction tools have rapidly evolved, facilitating the identification of plausible binding poses and interaction patterns in drug discovery workflows. For example, AlphaFold3 [6] predicts not only protein structures but also their interactions with other molecules such as protein–protein [7] and protein–peptide complexes [8]. Some emerging models, such as Boltz-2 [9], further attempt to estimate interaction strength and provide approximate affinity predictions to support drug discovery applications. PLIs involving small molecules can also be predicted using AI-based models, although the accuracy is limited by reliance on Protein Data Bank (PDB)-derived training data [10] and limited ligand diversity [11]. Traditional docking is still widely used for the prediction of binding poses and affinities [12]. Its computational cost is relatively low to allow structure-based screening of large compound libraries. However, docking methods often neglect explicit solvent molecules and rely on empirical scoring functions that are typically optimized for predicting binding affinity rather than correctly identifying the native binding conformation [13–15], often failing to reflect the true binding modes and making accurate binding estimation difficult [16,17]. To address these limitations, machine learning and deep learning approaches have been integrated into virtual screening [18,19] and docking [20–22], showing promise in capturing ligand features and improving affinity estimation. Nevertheless, these pose selection methods have shown only moderate improvements over classical scoring functions and remain limited by biased crystallographic training datasets and poor model interpretability [17]. In general, docking-based methods rely on static structure representation, and further improvement would be the incorporation of protein flexibility and conformational dynamics [23].

Molecular dynamics (MD) simulations are a powerful tool for exploring the dynamical aspects of PLIs over time, such as the stability of bound poses and binding-relevant conformational ensembles [24–26]. Because biologically relevant conformational transitions may occur on microsecond or longer time scales, canonical MD simulations are often insufficient to capture these dynamics within practical computational limits, hindering their use in rational drug design workflows [27]. To address this limitation, high-temperature MD (HT-MD) can be used as a rapid stress-testing approach to probe the robustness of protein–ligand binding modes under thermal perturbation, enabling efficient discrimination between stable and unstable poses within practical simulation times [28,29]. Although performed at elevated temperatures, HT-MD has been supported by experimental observations and provides valuable insights into binding dynamics [30,31]. The simulation trajectories can be analyzed using *R*-value analysis, which assesses binding stability by measuring the fraction of intermolecular contacts preserved between a target structure and a reference (e.g., an ensemble average) [32]. This method extends *Q*-value analysis, a framework that evaluates contact persistence between a target and the experimentally determined structure, for proteins, offering a dynamic, thermodynamically informed metric of binding stability [33]. *R*-value analysis has been successfully applied to identify the most probable binding poses in protein–small molecule and protein–peptide interactions [34–36]. Recently, HT-MD simulations have been combined with *R*-value analysis at scale in Dynamics DB, a database that stores, analyzes, and visualizes per-residue contact stability across a subset of 23,968 PDB entries [37]. This integration highlights the method’s feasibility, reproducibility, and potential for broad application in binding pose prediction.

In this study, we present a computational pipeline that integrates HT-MD simulations with *R*-value-based contact analysis to assess the stability of binding poses and explore residue-level ligand modifications. The pipeline is demonstrated on multiple experimentally determined protein–ligand complexes, illustrating how *R*-value analysis provides complementary information to static docking scores by capturing dynamic interaction patterns that are not accessible from docking alone. This workflow offers a practical, computationally efficient approach to support small-molecule lead prioritization and identify interactions contributing to binding stability, providing insights to guide future structure-based ligand optimization.

## Results

**Fig. 1.**
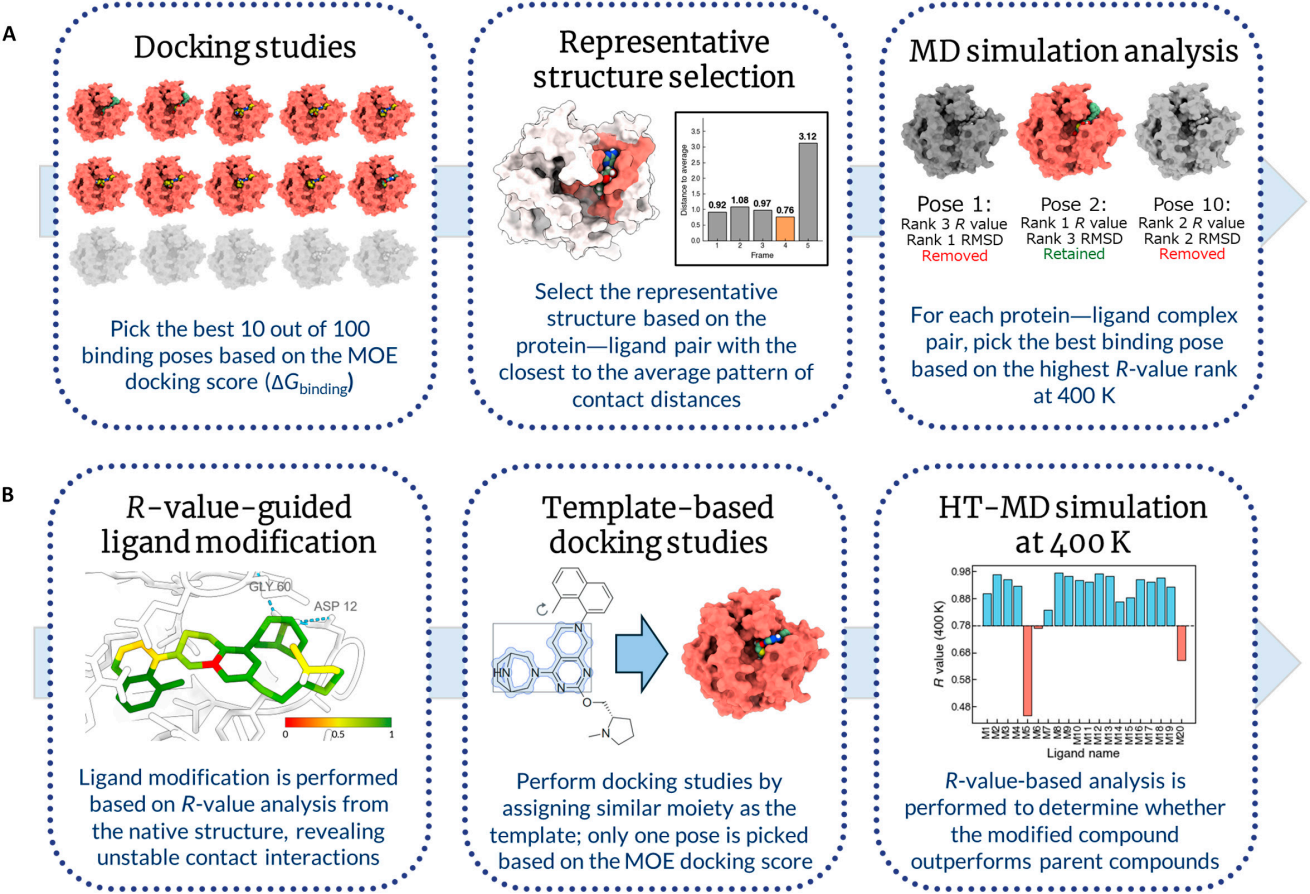
Workflow of the study. (A) Protocol for predicting and evaluating binding poses from template-free docking using high-temperature molecular dynamics (HT-MD) simulations and *R*-value analysis. (B) Extended workflow for ligand optimization, integrating template-based docking into HT-MD and enabling residue-level *R*-value analysis.

Our pipeline, which integrates HT-MD simulations and *R*-value-based contact analysis to validate binding poses and guide residue-level ligand optimization, is illustrated in Fig. 1. The pipeline uses multiple experimentally validated protein–ligand complexes (Table 1 and Table S1) to compute *R*-values from HT-MD trajectories, quantifying the fraction of intermolecular contacts preserved relative to an ensemble-derived reference. We validate our approach by comparing docking poses to experimental data (Fig. 1A) using both structural similarity (root-mean-square deviation [RMSD] to the experimental structure) and experimental binding affinity (*K*_d_). Residue-level stability features, such as persistent hydrogen bonds or van der Waals interactions, identify key binding hotspots and stable interaction motifs that could inform residue-level ligand optimization. These features provide interpretable insights that may guide medicinal chemistry efforts (Fig. 1B).

**Table 1.** List of the PDB structures used in this study

No.	Enzyme name	Enzyme classification	Total ligands	PDB ID as template (resolution)	Form for MD simulation	Ref.
1	Monkeypox VP39	Transferase-methyltransferase	6	8B07 (2.05 Å) [48]	Monomeric	
2	Set domain-containing protein 2 (SETD2)	Transferase-methyltransferase	8	5LT7 (1.51 Å) [111]	Monomeric	
3	Protein arginine methyltransferase 6 (PRMT6)	Transferase-methyltransferase	9	7NUD (1.65 Å) [112]	Dimeric	[113–115]
4	HIV-1 protease (HIV-1pro)	Hydrolase-protease	9	3OXC (1.16 Å) [116]	Dimeric	[55–61]
5	SARS-CoV-2 papain-like protease (PLpro)	Hydrolase-protease	14	8UOB (2.52 Å) [50]	Monomeric	[49,117]
6	ER glucosidase I (ERGluI)	Hydrolase-glycosidases	30	7R6J (1.91 Å) [51]	Monomeric	[118]
7	Kirsten rat sarcoma virus (KRAS^G12D^)	Hydrolase-GTPase	12	7RT1 (1.27 Å) [44]	Monomeric	[45,52]
8	Mammalian Ste20-like protein kinase 3 (MST3)	Kinase-protein kinase	12	8QLT (1.47 Å) [119]	Monomeric	[120–122]
9	Janus kinase 2 (JAK2) JH1 domain	Kinase-protein kinase	22	8BXH (1.30 Å) [43]	Monomeric	[53]
10	HMG-CoA reductase (HMGR)	Oxidoreductase	15	1HW8 (2.10 Å) [54]	Dimeric	[46,123]

PDB, Protein Data Bank; MD, molecular dynamics

### *R*-value in binding pose prediction: The higher the *R*-value, the closer it is to native

**Fig. 2.**
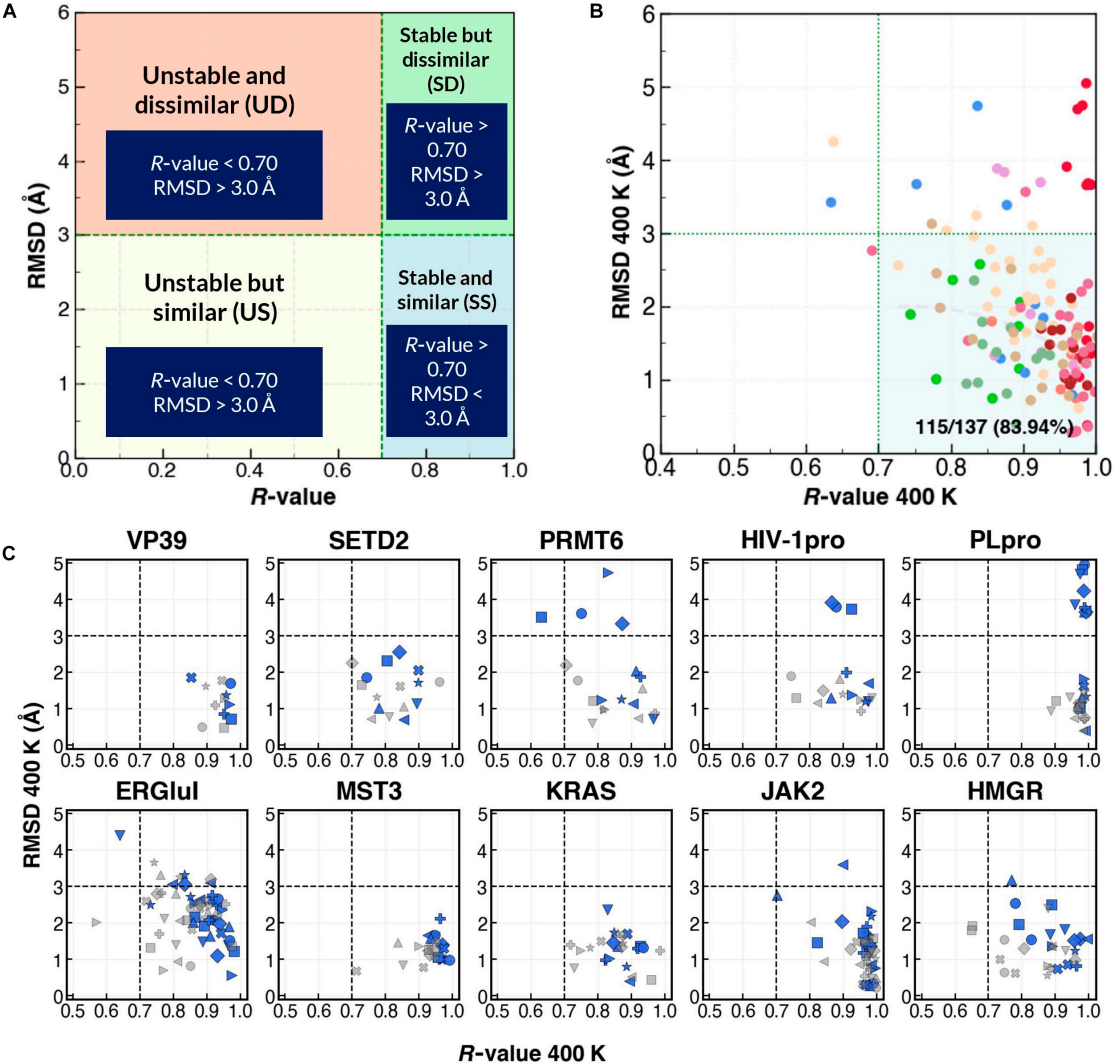
Assessment of docking-derived and experimentally derived ligand-binding poses using stability (*R*-value) and structural similarity (root-mean-square deviation [RMSD]) relative to the experimental structure across multiple systems. (A) Ligand pose classification into 4 categories: stable and similar (SS), stable but dissimilar (SD), unstable but similar (US), and unstable and dissimilar (UD). A pose is classified as SS if RMSD < 3.0 Å [16] and *R*-value > 0.70 [32]. (B) 400 K molecular dynamics (MD) simulation results from protein–ligand complexes initialized at top-ranked docking poses, based on the *R*-value, with corresponding RMSD values. (C) Per-system comparison of representative docking-derived poses (blue) and experimental poses (gray) across 10 systems; identical marker shapes indicate matched ligand identities.

Ligand-binding poses derived from computational studies are traditionally evaluated using heavy-atom RMSD, which quantifies geometric similarity to experimentally resolved structures. Specifically, RMSD is computed from the absolute coordinates of the ligand after protein superposition, thereby reflecting geometric deviation from the experimentally determined binding structure [38]. However, in predictive scenarios where experimental reference structures are unavailable, RMSD cannot be applied directly. We therefore investigated whether the *R*-value, a contact-based stability descriptor that measures the similarity and persistence of protein–ligand intermolecular contacts throughout the MD trajectory [32], can serve as a complementary indicator of native-like binding poses by examining whether thermodynamically stable configurations correspond to experimentally observed structures. To test this hypothesis, we performed HT-MD simulations at 400 K on 137 protein–ligand complexes across 10 systems initialized from their experimental structures. Two descriptors were used: (a) binding stability measured as the *R*-value from the 400 K MD representative structure (*R*(400K, repr)) and (b) pose similarity quantified as ligand heavy-atom RMSD relative to the experimental pose (RMSD(repr)). The representative structure was defined as the snapshot whose contact matrix most closely matches the trajectory-averaged contact matrix (Section S1). To integrate stability and structural similarity into a unified evaluation framework, poses were categorized into 4 classes: stable and similar (SS), stable but dissimilar (SD), unstable but similar (US), and unstable and dissimilar (UD) (Fig. 2A). The SS category represents structures that satisfy both stability and similarity criteria, as determined by selected *R*-value and RMSD thresholds.

To establish appropriate thresholds, we systematically examined the sensitivity of global and system-level %SS and Δ%SS to variations in *R*-value and RMSD cutoffs (Fig. S1a to d). At an RMSD threshold of 3.0 Å, global %SS remained stable between *R* = 0.60 and 0.70, with a marked decline only at *R* = 0.80, indicating that the selected *R*-value lies within a stability plateau and supporting the selection of 0.70 as the stability indicator, consistent with previous reports [34]. In contrast, reducing the RMSD cutoff to 2.5 or 2.0 Å decreased %SS and increased inter-system dispersion, as reflected by larger absolute Δ%SS values. These results indicate that stricter RMSD thresholds introduce sensitivity to system-specific flexibility under HT-MD conditions, leading to less consistent classification across datasets. A notable difference was observed for ERGluI (Δ%SS of 29.1 at the 0.70/2.5 Å threshold compared with 10.8 at 0.70/3.0 Å). Based on this robustness analysis, we adopted *R*(400K, repr) > 0.70 and RMSD(repr) < 3.0 Å to define the SS category, as this combination preserved high global performance while minimizing cross-system variability.

Using these criteria, 130 of 137 complexes (94.9%) were classified as SS (Table S2), demonstrating that HT-MD largely preserves experimental contacts [32] and native-like conformations [39]. A moderate inverse correlation between *R*(400K, repr) and RMSD(repr) (Fig. S2a) indicates that while the 2 metrics are related, they capture different aspects of binding, namely, interaction stability and geometric similarity. Comparison of *R*(400K, repr) with *R*(400K, exp), calculated directly from the experimental pose, showed that *R*(400K, repr) is consistently higher (mean 0.874 *vs.* 0.830; Fig. S2b and Table 2), indicating mild conformational adjustment rather than contact loss. Thus, whereas *R*(400K, exp) reflects the extent to which protein–ligand intermolecular contacts observed during the MD trajectory are maintained relative to the experimental binding structure, *R*(400K, repr) quantifies the persistence of intermolecular contacts relative to the simulation-derived representative pose and more effectively captures intrinsic binding stability. Because *R*(400K, repr) is calculated solely from the MD trajectory without requiring an experimental reference structure, it can be used in predictive docking scenarios to rank candidate poses based on their relative stability. Together, these results demonstrate that thermodynamic stability quantified by the *R*-value strongly associates with native-like binding geometry, supporting its use as a stability-informed surrogate indicator of pose similarity. We therefore use *R*(400K, repr) as the primary *R*-value metric and retain RMSD(repr) to assess geometric deviation.

**Table 2.** Summary of *R*-values and RMSD (mean values across ligands) from 400 K HT-MD simulations initiated from docking poses and experimental structures

No.	Enzyme name	HT-MD simulations from docking poses (most stable pose)				HT-MD simulations from experimental structure (PDB)			
		SS ligand pose (%)	*R*(400K, dock) value	*R*(400K, repr) value	RMSD(repr) (Å)	SS ligand pose (%)	*R*(400K, exp) value	*R*(400K, repr) value	RMSD(repr) (Å)
1	VP39	6 out of 6 ligands (100%)	0.8711	0.9449	1.2707	6 out of 6 ligands (100%)	0.9139	0.9252	1.1273
2	SETD2	8 out of 8 ligands (100%)	0.7806	0.8383	1.6704	8 out of 8 ligands (100%)	0.7742	0.8031	1.3928
3	PRMT6	5 out of 9 ligands (55.5%)	0.7561	0.8007	2.5943	9 out of 9 ligands (100%)	0.7846	0.8303	1.2132
4	HIV-1pro	6 out of 9 ligands (66.7%)	0.7974	0.9199	2.2482	9 out of 9 ligands (100%)	0.8042	0.8708	1.3928
5	PLpro	6 out of 14 ligands (42.9%)	0.9157	0.9820	2.8990	14 out of 14 ligands (100%)	0.9331	0.9607	1.0070
6	ERGluI	25 out of 30 ligands (83.33%)	0.7310	0.8927	2.2409	25 out of 30 ligands (83.33%)	0.8017	0.8261	2.2319
7	KRAS^G12D^	12 out of 12 ligands (100%)	0.7682	0.8742	1.3201	12 out of 12 ligands (100%)	0.7629	0.8423	1.2148
8	MST3	12 out of 12 ligands (100%)	0.8316	0.9590	1.3676	12 out of 12 ligands (100%)	0.8071	0.8976	1.1555
9	JAK2	21 out of 22 ligands (95.4%)	0.8204	0.9490	1.4513	22 out of 22 ligands (100%)	0.9363	0.9590	1.0476
10	HMGR	14 out of 15 ligands (93.3%)	0.7221	0.8959	1.6620	13 out of 15 ligands (86.67%)	0.7471	0.8195	1.2261
All systems	115 out of 137 ligands (83.9%)	0.7994	0.9056	1.8724	130 out of 137 ligands (94.89%)	0.8265	0.8735	1.3009

RMSD, root-mean-square deviation; HT-MD, high-temperature molecular dynamics; PDB, Protein Data Bank; SS, stable and similar.

To assess the applicability of our stability-based framework in distinguishing plausible binding poses from decoys, we generated 10 docking poses per protein–ligand pair (137 systems; 1,370 total) using MOE 2022.02. All poses were advanced to HT-MD system preparation without additional score-based filtering. During setup and equilibration, 22 poses failed to initialize due to unstable starting conformations (e.g., severe steric clashes) and were excluded. The remaining 1,348 poses were successfully equilibrated and subjected to HT-MD production and subsequent analysis. An inverse relationship between *R*-value and RMSD(repr) was observed (Fig. S3), although substantial deviations were present across poses; 44% of poses were SS and 34.7% were US (Table 3), indicating that geometric similarity does not guarantee thermodynamic stability. Here, the *R*-value was used as a ranking criterion within each docking ensemble to identify the most stable pose. Notably, 83.9% of ligands (115/137) had their top-ranked pose classified as SS (Fig. 2B and Table S3), with an average *R*-value increase of 0.11 relative to the initial *R*(400K, dock) (Table 2), demonstrating the ability of HT-MD to refine suboptimal conformations while preserving key contacts. Four systems (VP39, SETD2, KRAS^G12D^, and MST3) achieved 100% SS success, underscoring both the quality of docking and HT-MD’s refinement power. Poses with RMSD(dock) > 2.0 Å generally improved during 400 K MD, although the extent varied by system (Fig. S4). This aligns with previous findings [25,40]; native-like poses remain stable, while dissimilar ones often undergo marked refinement. SD poses may reflect experimental heterogeneity (e.g., PLpro with R1/R2-swapped electron density maps; Fig. S5), while US/UD outliers indicate stable alternative conformations driven by weak interactions or crystallization artifacts (e.g., ERGluI; Section S2 and Figs. S5 to S7).

**Table 3.** Summary of pose classification by *R*-value and RMSD (post-MD, 400 K) for each target system. Poses are grouped into 4 categories: SS, SD, US, and UD (as in Fig. 2A).

System	Total ligands	Total poses	%SS	%SD	%US	%UD	nSS	nSD	nUS	nUD
VP39	6	60	60	0	20	20	36	0	12	12
SETD2	8	80	45	0	32.5	22.5	36	0	26	18
PRMT6	9	87	29.89	8.05	29.89	32.18	26	7	26	28
HIV-1pro	9	90	27.78	15.56	25.56	31.11	25	14	23	28
PLpro	14	140	24.29	37.86	20.71	17.14	34	53	29	24
ERGluI	30	286	40.21	6.29	39.51	13.99	115	18	113	40
MST3	12	119	84.87	0	15.13	0	101	0	18	0
KRAS^G12D^	12	119	60.5	1.68	37.82	0	72	2	45	0
JAK2	22	220	47.27	3.18	42.27	7.27	104	7	93	16
HMGR	15	147	29.93	3.4	56.46	10.2	44	5	83	15
All systems	137	1,348	43.99	7.86	34.72	13.43	593	106	468	181

RMSD, root-mean-square deviation; MD, molecular dynamics; SS, stable and similar; SD, stable but dissimilar; US, unstable but similar; UD; unstable and dissimilar; %, fraction of total poses per system; n, raw count.

To evaluate docking performance at the system level, we compared top-ranked pose accuracy, docking ensemble coverage, and HT-MD rescue performance (Table 4). The top-ranked docking pose reproduced native-like conformations (RMSD < 3.0 Å) in 78.8% of cases. Nevertheless, native-like poses were present in 98.3% of docking ensembles, indicating that docking effectively sampled correct binding modes even when scoring failed to rank them first. Notably, among cases where docking failed at top 1 (21.2%), HT-MD refinement recovered correct and stable conformations in ~86% of these failures (25/29), highlighting its effectiveness as a postdocking validation step that identifies conformations satisfying both geometric and stability criteria.

**Table 4.** System-level docking and HT-MD performance, showing top-1 hit rates, dock success rates, and HT-MD rescue of correct poses following docking failure

No.	System	Total ligands	Top-1 hit rate (%)	Dock success rate (%)	HT-MD rescued poses
1	VP39	6	100.00	100.00	0/0
2	SETD2	8	75.00	100.00	2/2
3	PRMT6	9	66.67	88.89	1/3
4	HIV-1pro	9	44.44	100.00	4/5
5	PLpro	14	64.29	100.00	5/5
6	ERGluI	30	80.00	100.00	6/6
7	MST3	12	100.00	100.00	0/0
8	KRAS	12	100.00	100.00	0/0
9	JAK2	22	81.80	100.00	4/4
10	HMGR	15	73.33	100.00	3/4
All systems	137	78.83	99.27	25/29

HT-MD, high-temperature molecular dynamics; Top-1 hit rate, percentage of cases with top-ranked docking pose root-mean-square deviation (RMSD) < 3.0 Å; Dock success rate, percentage of cases with at least one docking pose RMSD < 3.0 Å; HT-MD rescued poses, top-1 failures (RMSD > 3.0 Å) rescued by HT-MD (RMSD < 3.0 Å and *R*-value ≥ 0.70), shown as rescued/failed cases.

Comparison of docking- and experimental-structure-initiated simulations (Fig. 2C) reveals system-dependent behavior. In VP39, MST3, and KRAS^G12D^, docking-derived poses converged to stable, experimental-structure-like conformations. In contrast, PRMT6 and PLpro showed greater stability when initiated from experimental structures, likely due to either (a) alternate binding modes not resolved in crystallography or (b) docking’s inability to generate the correct stable complex, although such cases were limited (only 2 ligands classified as UD). Together, these demonstrate that HT-MD reliably refines plausible poses into thermodynamically stable, experimentally relevant conformations, with RMSD(repr) < 3.0 Å serving as a practical indicator of native binding modes.

### *R*-value correlates with experimental binding affinity (*K*_d_)

To evaluate the extent to which the *R*-value from HT-MD simulations correlates with experimental *K*_d_, we analyzed representative structures generated from experimental complexes (Fig. S2a). We further performed residue-level stability analysis to explain the observed affinities and identify key residues contributing to binding interactions. The rationale for correlating *R*-value with *K*_d_ lies in the fundamental relationship $K_{d}=k_{\mathrm{off}}/k_{\mathrm{on}}$ [41]. Conceptually, the *R*-value is more closely related to the ligand dissociation rate constant (*k*_off_), as it reflects the persistence of PLI contacts during MD simulations. A higher *R*-value indicates greater interaction stability and is therefore qualitatively consistent with slower dissociation. Accordingly, for structurally similar ligands binding to the same site in comparable binding modes, where the ligand association rate constant (*k*_on_) is expected to vary within a relatively narrow range, differences in *K*_d_ are likely to be primarily governed by *k*_off_ [42]. Under these conditions, the *R*-value can serve as a stability-informed surrogate for *K*_d_. From a thermodynamic perspective, binding affinity is governed by the free energy of binding (Δ*G*), which is directly related to *K*_d_ [41]. In this context, the persistence of favorable PLIs captured by the *R*-value may also reflect stabilizing energetic contributions to ligand binding. Thus, within structurally similar ligand series, the *R*-value can be interpreted from both kinetic and thermodynamic perspectives.

**Fig. 3.**
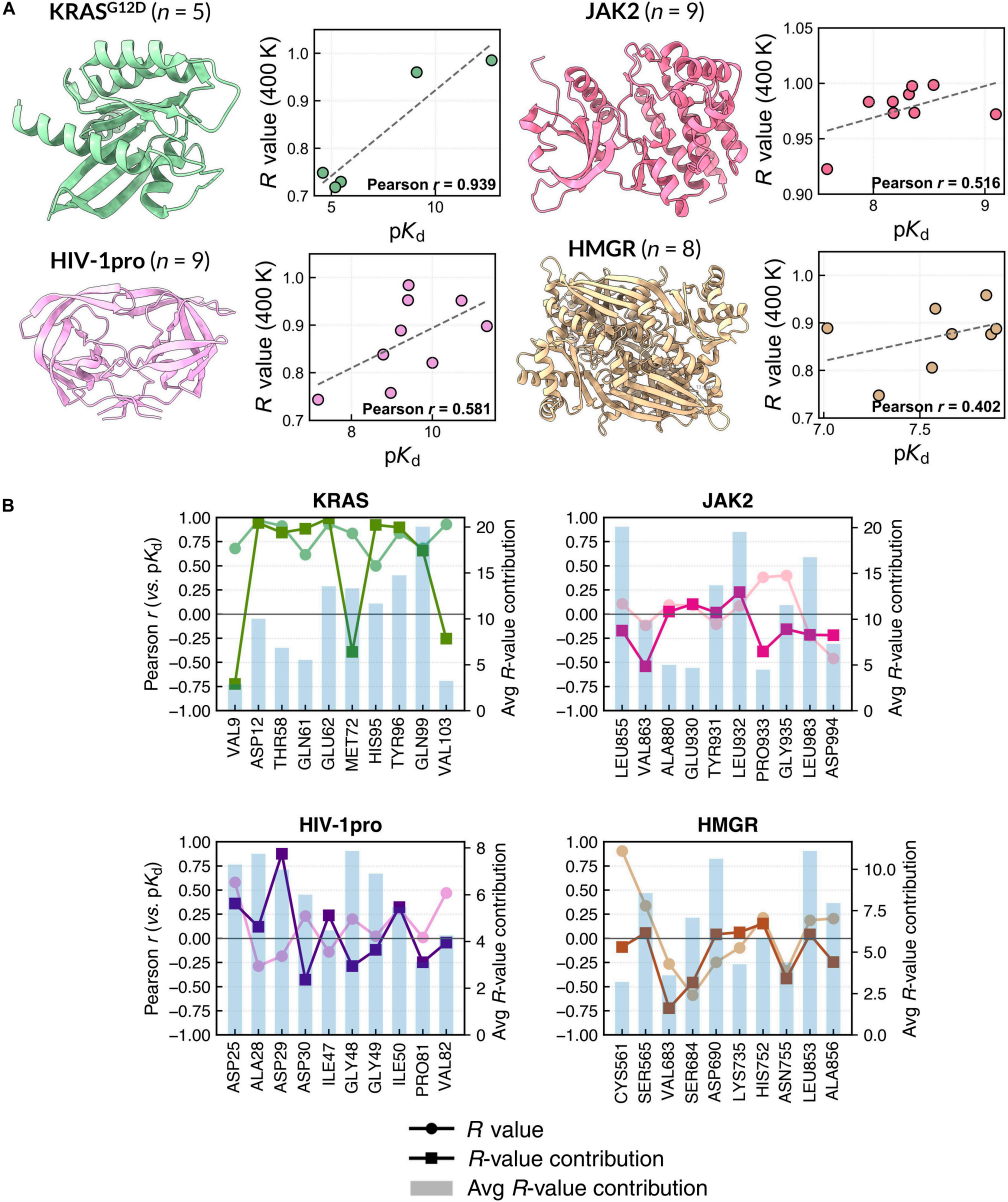
Correlation between high-temperature molecular dynamics (HT-MD)-derived *R*-values and experimental binding affinities. (A) Scatter plots of p*K*_d_ versus *R*-value at 400 K for JAK2, KRAS^G12D^, HIV-1pro, and HMGR; *R*-values are from experimental-structure MD simulation. Sample sizes (*n*) indicate the number of ligands with reported *K*_d_ values; full data in Table S4. (B) Per-residue correlation between *R*-value (circles) and p*K*_d_ (squares) across 4 systems.

We evaluated 4 systems (JAK2, KRAS^G12D^, HMGR, and HIV-1pro) selected based on the availability of experimental *K*_d_ values (from the literature [43–46] or public databases, e.g., ChEMBL [47], Table S4). In the remaining systems, only IC_50_ [44–46,48–54] or *K*_i_ values [50] were available; however, due to the assay-dependent nature of IC_50_ and the model-dependent derivation of *K*_i_ and the context-dependent relationship of these metrics to equilibrium binding affinity, these metrics were not considered directly comparable to *K*_d_. Consistent with this, IC_50_- and *K*_i_-based analyses exhibited inconsistent correlation patterns across systems (Fig. S8) and were therefore excluded from the quantitative correlation assessment. For each system, we compared experimental *K*_d_ values with the *R*-values of protein–ligand complexes initiated from experimental structures. Since the experimental structures capture the lowest-energy binding mode, we expect native-like poses to retain high contact fractions under the elevated-temperature simulations; thus, complexes with *R*-value < 0.70 were classified as non-native-like and excluded. Across all systems, the Pearson correlation between *R*-value and p*K*_d_ ranged from 0.40 to 0.94 (Fig. 3A), indicating that higher contact stability correlates with stronger binding affinity. For HMGR, the 0.40 correlation emerged only after excluding compound 7 (*R*-value = 0.64; *K*_d_ = 26.9 nM), which was outside the native-like regime (*R*-value < 0.70) and thus omitted (Fig. S9). In KRAS^G12D^, a strong correlation highlights dynamic stability as a key driver of affinity in rigid pockets, suggesting that stability-based metrics may guide the design of higher-affinity inhibitors. JAK2, although relatively rigid, exhibits greater dynamics than KRAS^G12D^; its Pearson correlation coefficient (*R* = 0.52) reflects a positive but less pronounced linear relationship. Notably, this trend is strongly influenced by ruxolitinib, which shows the lowest *K*_d_ but only the eighth highest *R*-value, thereby diminishing the overall correlation strength. Weaker correlations in HMGR and HIV-1pro may arise from increased conformational flexibility, solvent accessibility, or experimental heterogeneity (e.g., ligands from different structural studies under varying conditions [55–61]). Collectively, these results show that contact stability (via the *R*-value) can serve as a predictor of binding affinity in relatively rigid, native-like binding pockets, while conformational dynamics and experimental variability may weaken this relationship in more flexible or variably characterized systems.

To confirm whether specific residues directly contribute to ligand-binding affinity, we performed correlation analysis between the per-residue *R*-value and its *R*-value contribution (defined as the unnormalized per-residue *R*-value; see Section S1) against the experimental p*K*_d_ values for all ligands across the 4 representative systems (Fig. 3B and Fig. S10). In the KRAS^G12D^ system, the top 5 residues, Glu62, Gly60, Asp12, His95, and Asp69, show the strongest positive correlation between the *R*-value and p*K*_d_ (*r* ≈ 0.8), consistent with their roles in anchoring the ligand through hydrogen bonding. The stability of these interactions also appears to influence nearby residues (e.g., Tyr65, Tyr96, and Gln99), suggesting that local stabilization in this region contributes significantly to ligand potency. In the HIV-1pro system, similar positive correlations were also observed in several key residues across multiple ligands, such as Asp29 and Ile50, although the correlations were weaker than those in the KRAS^G12D^ system. For JAK2, most residues showed weak *R*-value–p*K*_d_ correlations, except for Val863 and Gly935, which had moderate positive ones. Excluding ruxolitinib (Fig. S11) improved overall correlations, with Leu855 (*r* = 0.75; previously −0.17) and Gly935 (*r* = 0.54; previously −0.16) showing moderate to strong negative correlations. Leu855 is part of the P-loop in the ATP-binding site and is known to be important for ligand recognition [53]. Finally, in the HMGR system, no strong positive correlations were observed across residues, with only relatively moderate negative correlations for Val683 and Ser684. Interestingly, Cys561 displayed a relatively strong positive correlation between *R*-value and binding affinity, consistent with its role as a catalytic residue in the HMGR inhibitory site [62].

We examined the %Contribution of each residue to the *R*-value to identify key stabilizing interactions. As shown in Fig. 3B, residues contributing to hydrophobic interactions dominate in KRAS^G12D^ (Gln99, Met72, and Tyr96) and JAK2 (Leu888, Leu922, and Leu983), collectively accounting for >35% of total contact stability, despite the absence of strong hydrogen bonds. Notably, although Gln99 is a polar residue, it does not form hydrogen bonds with any KRAS^G12D^ ligands and instead contributes through van der Waals interactions (Fig. S12), consistent with the predominantly hydrophobic nature of the binding pocket. This highlights the importance of persistent van der Waals and π–alkyl interactions in hydrophobic cores, which complement directional hydrogen-bond interactions (e.g., Glu62 and Asp12) that anchor ligand positioning and together contribute to binding affinity. In contrast, HIV-1pro and HMGR show greater contributions from polar residues: Asp25/Asp29 in HIV-1pro (catalytic residues) [63] and Asp690/Ser565 in HMGR (active site) [64]. HIV-1pro exhibits a more evenly distributed contribution, consistent with its flexible flap and dynamic binding pocket [63]. Overall, mapping residue-level %Contribution onto structurally and functionally annotated binding sites indicates that the highest *R*-value contributions coincide with catalytically important and functionally relevant interface residues, based on literature-reported catalytic and active-site annotations. While *R*-value–p*K*_d_ correlations link stability to affinity, %Contribution identifies residues that dominate overall contact persistence through hydrophobic, hydrogen-bond, or π-stacking interactions. Together, these results demonstrate that the *R*-value framework, in conjunction with its observed correlation with binding affinity, can quantitatively identify critical interaction hotspots for ligand stabilization.

### Stability-guided, SAR-like analysis enables ligand optimization in KRAS^G12D^

Next, we present a case study for lead optimization to demonstrate the application of the stability-driven *R*-value framework in guiding inhibitor design. We emphasize that this analysis serves as a methodological demonstration rather than a definitive ligand optimization effort. Although no experimental validation was performed for the newly generated compounds, the previously established correlation between *R*-value and p*K*_d_ across multiple systems provides a rationale for this approach. Accordingly, the results should be interpreted as hypothesis generating and illustrated how the framework may support structure–activity relationship (SAR) exploration. Here, template-based (core-constrained) docking was used to preserve experimentally validated binding modes while introducing targeted modifications. Although this constrains initial pose generation, stability was subsequently evaluated using fully flexible MD simulations, enabling an unbiased assessment independent of the starting configuration.

We selected KRAS^G12D^ because the *R*-value showed the strongest correlation with the experimentally reported *K*_d_ (Pearson *r* = 0.94; Fig. 3A). We then expanded the dataset by adding 9 structurally diverse compounds (compounds 6 to 14) and 4 known inhibitors (TH-Z835, Cp5, Cp8, and Cp14), for which *K*_d_ values were approximated from reported IC_50_ measurements. The inverse correlation remained robust, albeit slightly attenuated (*r* = 0.81 with 9 additional compounds; *r* = 0.80 including all compounds with estimated affinities; Fig. S13a). While per-residue correlations with p*K*_d_ weakened slightly with the expanded set (Fig. S13b), Glu62, Gly60, Asp12, His95, and Asp69 consistently ranked as top contributors. Glu62 showed the strongest correlation (*r* = 0.82).

**Fig. 4.**
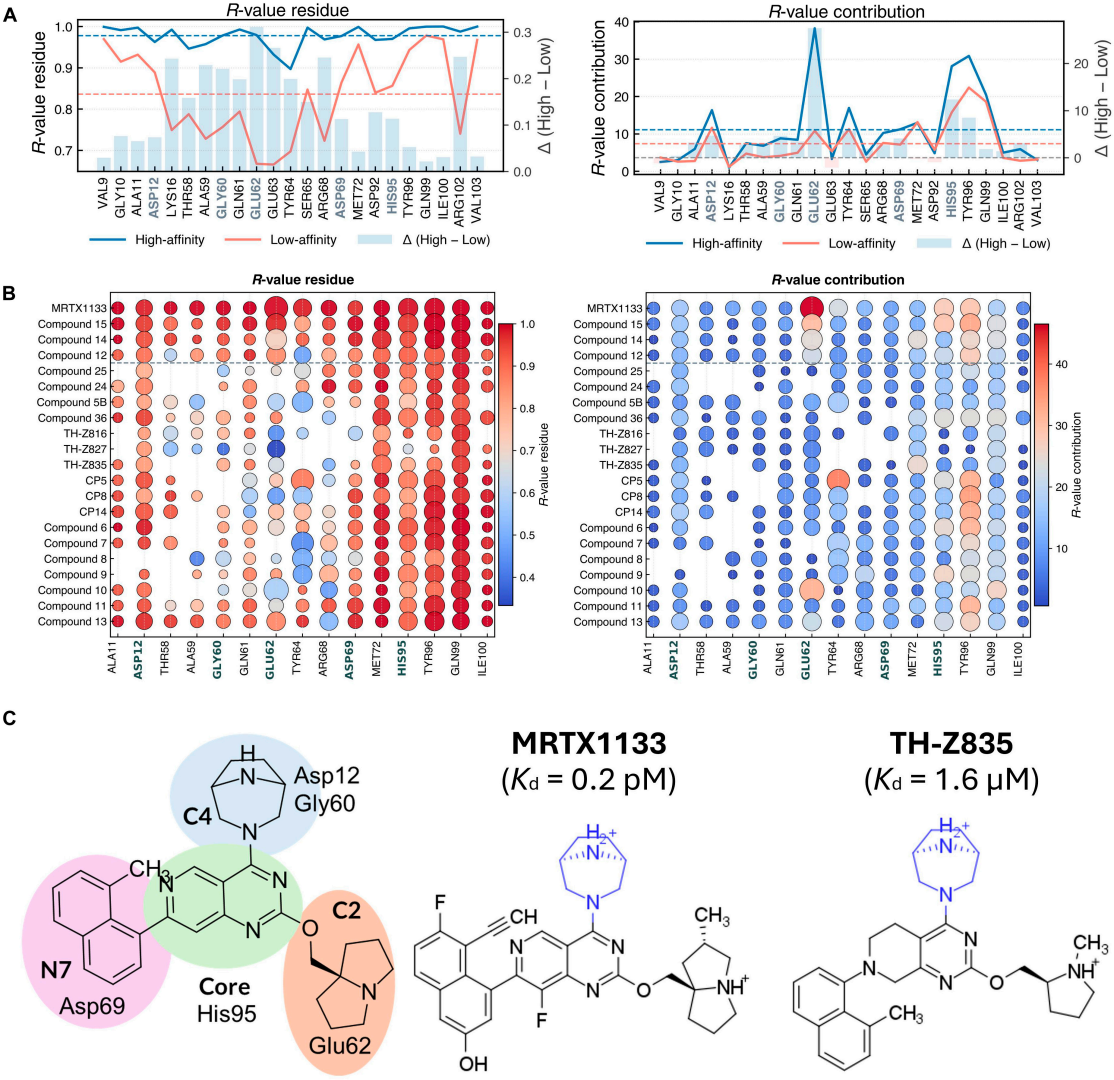
Residue-level *R*-value analysis for structure–activity relationship (SAR)-like evaluation of KRAS^G12D^ inhibitors. (A) Average residue-wise *R*-value and *R*-value contribution for high-affinity (*K*_d_ < 10 nM) and low-affinity (*K*_d_ > 10 nM) ligands; blue bars show average *R*-value differences. (B) Per-compound residue-level *R*-value and contribution; Asp12, Gly60, Asp69, His95, and Glu62 are key stability anchors (bolded, dark gray). Ligands are ordered by affinity, with high-affinity compounds above the dashed divider. (C) SAR-like map of the KRAS^G12D^ binding site with representative structures of MRTX1133 (high-affinity) and TH-Z835 (low-affinity).

Per-residue analysis reveals clear distinctions between high-affinity (*K*_d_ < 10 nM) and low-affinity (*K*_d_ > 10 nM) ligands. High-affinity ligands maintain strong contacts with Glu62 (~14% contribution *vs.* ~6% in low-affinity ligand) and higher total contact counts (~40 *vs.* ~10), reinforced by Asp12, Gly60, and His95 (Fig. 4A and B). Among the analyzed compounds, MRTX1133 and compound 15 exhibit the highest stability and affinity, sharing a [3.2.1] bicyclic diamino substituent that forms persistent hydrogen bonds with Gly60 and Asp12, with the secondary, protonated amine acting as a donor [44]. This interaction network extends through Asp69 (N7-linking group) and His95 (heterocyclic core), stabilizing durable Glu62 interactions via the C2 linker (Fig. 4C). In contrast, low-affinity ligands (e.g., TH-Z835, Cp5, Cp8, and Cp14) contain the same scaffold but lack key hydrogen-bond donors (e.g., at N7 in TH-Z835 or suboptimal Gly60 engagement), reducing stability and potency. Taken together, per-residue *R*-value analysis highlights interaction hotspots that may inform SAR exploration in KRAS^G12D^ inhibitors.

Based on this stability-guided, SAR-like analysis, we outline 2 general design considerations for developing high-affinity KRAS^G12D^ inhibitors: (a) reinforcing the 4-residue interaction network (Asp12, Gly60, Asp69, and His95) through functional group modifications that strengthen Glu62 engagement via hydrogen bonding and (b) applying these insights to scaffold optimization. As a proof of concept, we modified TH-Z835 to incorporate this design principle and analyzed MRTX1133 as a reference system to evaluate whether regions of comparatively lower contact persistence could be further stabilized. This example is intended to illustrate the sensitivity of the *R*-value framework and how contact persistence analysis can guide rational structural hypotheses and provide concrete strategies for functional group modification, particularly for lower-affinity inhibitors, rather than to suggest practical improvement over an already highly optimized inhibitor. The proposed modifications are hypothesis generating and require experimental validation to confirm their impact on affinity.

#### Ligand optimization of TH-Z835

**Fig. 5.**
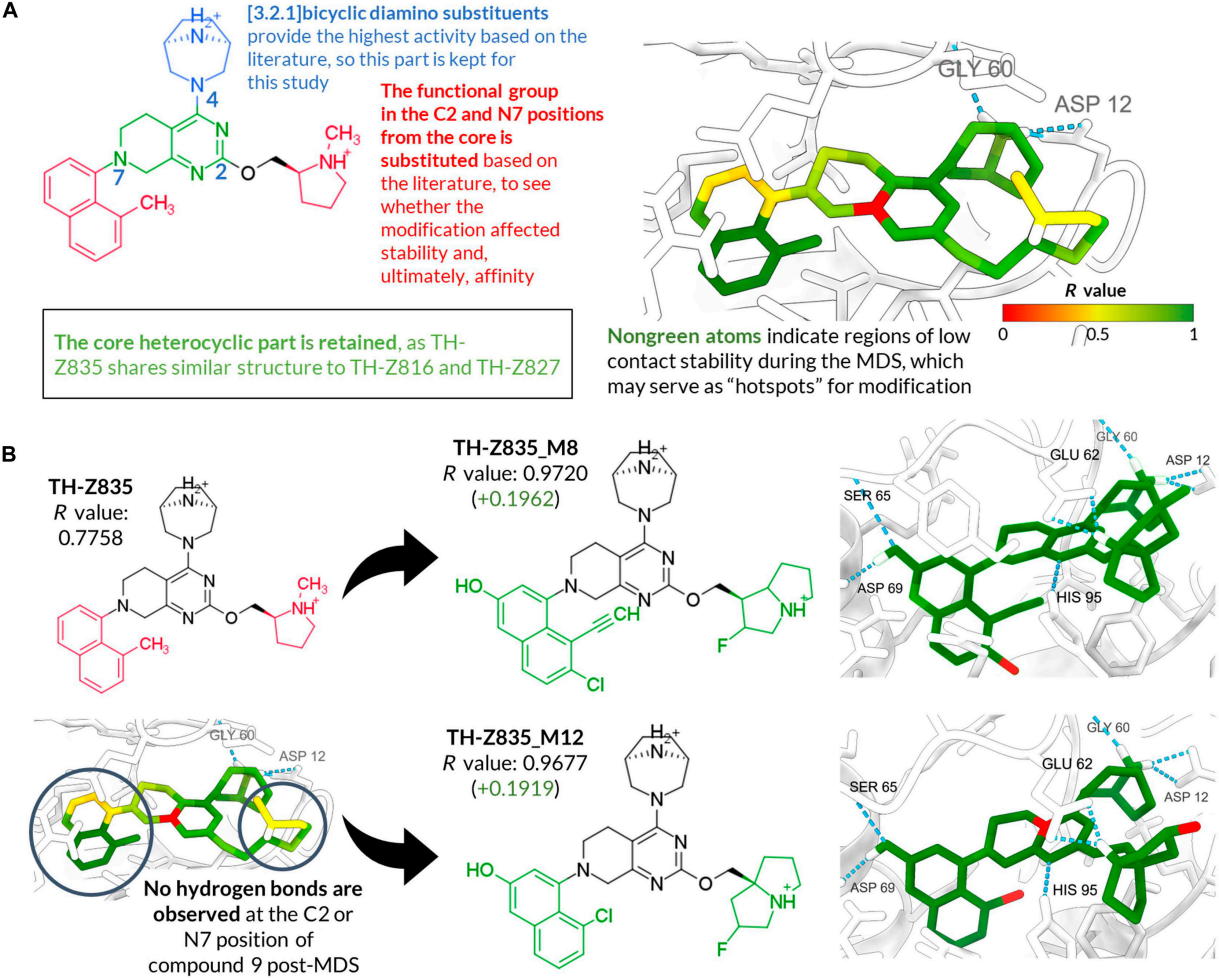
Dynamic-driven structure–activity relationship (SAR) analysis and optimization of TH-Z835. (A) High-temperature molecular dynamics (HT-MD) structure of TH-Z835 with atomic-level stability mapped by *R*-value (green, stable; yellow, less stable; red, unstable/no contacts). (B) Comparison of TH-Z835 with derivatives TH-Z835_M8 and TH-Z835_M12; chemical structures and binding site views with *R*-values indicated. Key residues (Asp12, Gly60, Asp69, His95, and Glu62) are highlighted.

TH-Z835 (IC_50_ = 4.8 μM) contains the [3.2.1] bicyclic diamino motif found in high-affinity KRAS^G12D^ inhibitors but lacks an N7 substituent to anchor to Asp69, which may weaken Glu62 stabilization and limit hydrogen-bond formation with Asp12, Gly60, and His95 (Fig. 5A). To improve stability, we designed 20 analogs with modifications at C2, N7, and the heterocyclic core (Table S5); performed template-based docking; and evaluated them via HT-MD and *R*-value analysis. Of these, 18 showed higher overall stability (*R*-value > 0.85; Table 5), with 13 exceeding 0.90. In the original compound, HT-MD revealed no persistent N7 hydrogen bonds, resulting in weak Asp69 interaction and low contact counts. In contrast, TH-Z835_M8 (M8) and TH-Z835_M12 (M12), which exhibit the highest *R*-values among the designed analogs, introduce an aromatic −OH at N7 that is predicted to form a stable hydrogen bond with Asp69, as well as a fluorine-conjugated pyrrolizine at C2 (similar to MRTX1133) providing superior Glu62 stabilization over the original pyrrolidine. These combined modifications are associated with increased hydrogen bonding and overall interaction stability, with Δ*R* = +0.20 (M8) and +0.19 (M12) (Fig. 5B).

**Table 5.** Analysis of the C2, N7, and core heterocyclic substitution in TH-Z835 analogs and their HT-MD simulation results

Compound name	*R*-value
TH-Z835_M1	0.8955
TH-Z835_M2	0.9645
TH-Z835_M3	0.9452
TH-Z835_M4	0.9231
TH-Z835_M5	0.4428
TH-Z835_M6	0.7658
TH-Z835_M7	0.8342
TH-Z835_M8	0.9720
TH-Z835_M9	0.9596
TH-Z835_M10	0.9424
TH-Z835_M11	0.9365
TH-Z835_M12	0.9677
TH-Z835_M13	0.9580
TH-Z835_M14	0.8651
TH-Z835_M15	0.8805
TH-Z835_M16	0.9475
TH-Z835_M17	0.9361
TH-Z835_M18	0.9536
TH-Z835_M19	0.9198
TH-Z835_M20	0.6466
TH-Z835	0.8485

HT-MD, high-temperature molecular dynamics

**Fig. 6.**
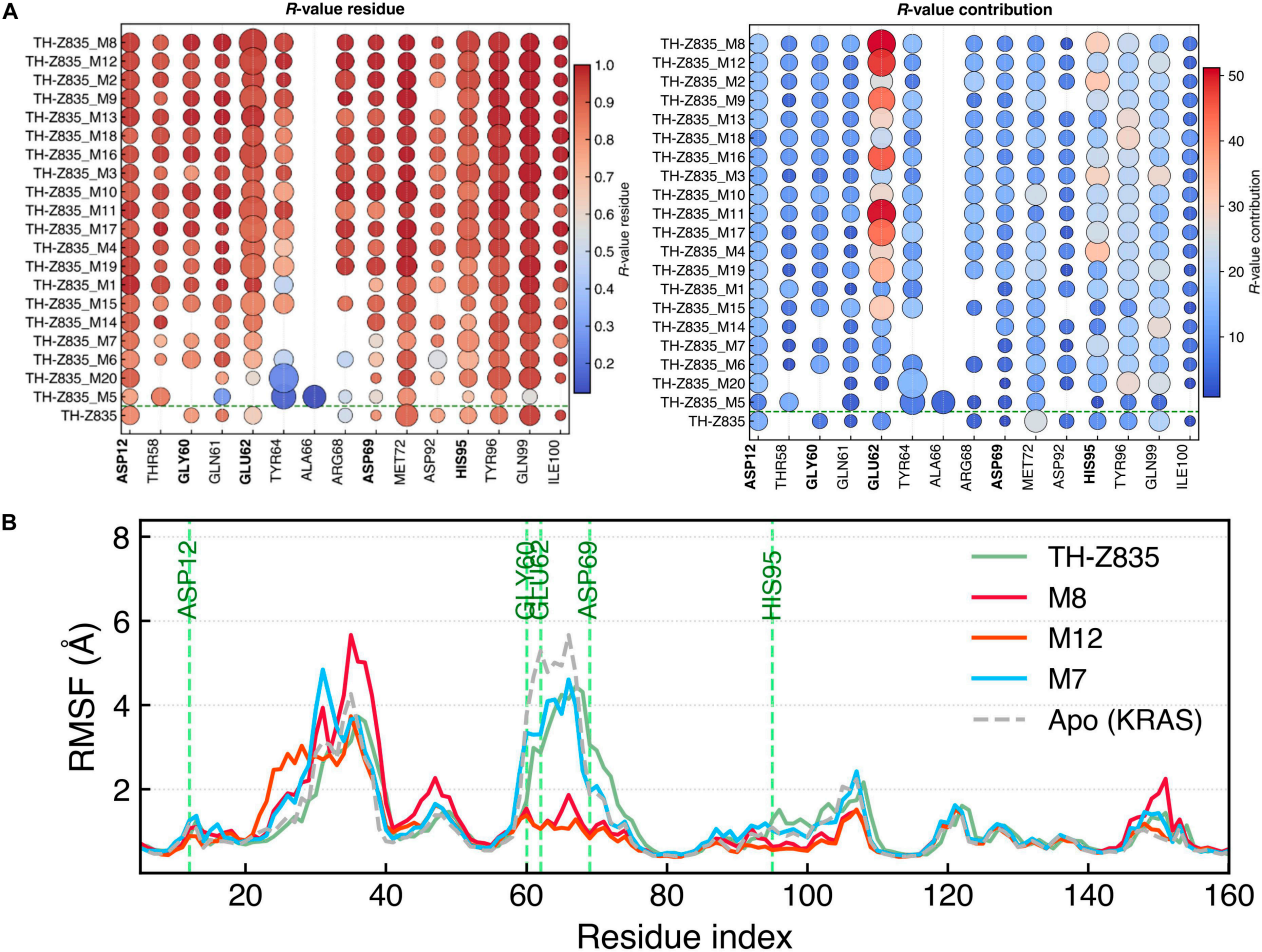
Residue-level maps and root-mean-square fluctuation (RMSF) profiles for TH-Z835 and its analogs (TH-Z835_M*x*). (A) Residue-level *R*-value and contribution plots; ligands ordered by reference *R*-value (highest at the top); the green dashed line marks original TH-Z835. Circle area proportional to contact count; color encodes metric (blue, low; red, high). Key anchoring residues (Asp12, Gly60, Glu62, Asp69, and His95) are bolded. (B) RMSF profiles for TH-Z835, M8, M12 (high-stability), and M7 (low-stability); KRAS^G12D^ apo structure shown as reference.

To characterize the dynamic behavior of TH-Z835 and its designed analogs, we performed residue-level *R*-value and *R*-value contribution analyses (Fig. 6A) together with root-mean-square fluctuation (RMSF) analysis (Fig. 6B) to assess protein flexibility. Analogs lacking an Asp69-engaging substituent (the original compound and M5 to M7) showed weak Asp69-binding stability, accompanied by lower *R*-values for Asp12, Gly60, and Glu62 and increased fluctuations in the Gly60–Asp69 region. The original compound’s fluctuation profile resembled the apo structure, suggesting limited stabilization of the binding pocket. In contrast, analogs containing an Asp69-directed substituent showed tighter RMSF profiles, higher *R*-values across key residues, and overall lower fluctuations. This stabilization appears to propagate to Glu62, supporting its proposed role as the central anchoring residue in the KRAS^G12D^ binding pocket. Engagement of Glu62 was further enhanced when Asp12, Gly60, Asp69, and His95 were concurrently stabilized, whereas disruption of any of these anchors was associated with reduced Glu62 stability and overall ligand affinity. Collectively, these findings suggest that Glu62 may serve as a key anchoring residue for future scaffold modification and ligand optimization strategies.

#### Ligand optimization of MRTX1133

MRTX1133 (*K*_d_ = 0.2 pM) exhibits near-maximal stability at 0.985, but residue-level analysis identifies a weak point at C30 (*R*-value = 0.70) due to unfavorable electrostatic interactions with Lys88 (Fig. S14a), which may influence the stability of the Glu62 hydrogen bond, consistent with prior molecular mechanics Poisson–Boltzmann surface area studies showing Lys88’s positive electrostatic contribution [65]. To explore this feature, we designed 15 C30-modified analogs by introducing polar or anionic/polarizable functional groups to reduce cation–cation repulsion while preserving Glu62 binding (Table S6). Most analogs show slightly reduced *R*-values (down to 0.94 for M5), but M1 and M12 show marginal increases in stability (≈0.99). M1 (carbonyl at C30) is predicted to form a hydrogen bond with Lys88, increasing the *R*-value by 0.005; M12 (–CF_3_) may reduce unfavorable contacts with Lys88, resulting in an increase in *R*-value of 0.006 (Table 6 and Fig. S14b). Residue-level *R*-value and RMSF analyses (Fig. S15a and b) suggest that Lys88 interactions act as a modulator of local stability: while M11 and M14 stabilize Lys88, they are associated with reduced Glu62 stability and lower overall *R*-values. M13 shows weak Lys88 interaction but better overall stability than M11/M14. Backbone flexibility remains similar across compounds, with Lys88 slightly less dynamic in MRTX1133. These results highlight Lys88 as a modulatory residue influencing Glu62 stability and demonstrate the sensitivity of the *R*-value framework in detecting subtle stability differences, rather than indicating practical improvement of an already highly optimized ligand.

**Table 6.** Examination of the C30 substitution of MRTX1133 analogs and their HT-MD simulation results

No.	Compound name	*R*-value	No.	Compound name	*R*-value
1	MRTX1133_M1	0.9907	9	MRTX1133_M9	0.9824
2	MRTX1133_M2	0.9617	10	MRTX1133_M10	0.9751
3	MRTX1133_M3	0.9708	11	MRTX1133_M11	0.981
4	MRTX1133_M4	0.9721	12	MRTX1133_M12	0.9918
5	MRTX1133_M5	0.9412	13	MRTX1133_M13	0.9815
6	MRTX1133_M6	0.9589	14	MRTX1133_M14	0.9795
7	MRTX1133_M7	0.9788	15	MRTX1133_M15	0.9477
8	MRTX1133_M8	0.9813	16	MRTX1133	0.9854

HT-MD, high-temperature molecular dynamics

## Discussions

Integrating MD simulations into rational drug design for binding pose validation and ligand optimization remains a key challenge in computer-aided drug discovery. Docking and pharmacophore modeling dominate due to speed and robustness, but they lack dynamic accuracy and rely on simplified scoring functions [14,66]. MD simulations address these by capturing conformational flexibility and interactions yet remain limited by high computational cost for microsecond-scale sampling [27,67]. Previous studies have examined ligand-binding modes using an integrated docking–MD approach based on RMSD analysis [68,69] and protein–ligand interaction fingerprint-based scoring function [70,71], as well as assessing residue-level dynamics and unbinding behavior in protein–ligand complexes using MD simulations [72,73], highlighting the importance of dynamic metrics in rationalizing binding affinity and binding kinetics. More recently, a study applied multiple short MD simulations combined with contact- and pocket-based descriptors to predict fragment binding modes [74]. However, many of these approaches start from experimentally determined structures, and methods that rely on RMSD as the primary selection criterion for docking poses [68,69] may not be directly applicable to novel compounds lacking structural data.

Here, we present a stability-guided HT-MD framework that aims to bridge static docking predictions and full dynamics, applicable to a range of structurally diverse targets. Using *R*-value-based contact persistence analysis from high-temperature trajectories, we show that binding pose stability is associated with experimentally validated structures and binding affinities. The *R*-value, used here as a contact persistence metric, shows correlations with experimental p*K*_d_ (*r* = 0.40 to 0.94). This trend is consistent with prior work at 400 K showing correlations with experimental melting temperatures [30] and with relative binding free energies (RBFEs) [75], with correlations generally higher at 400 K than at 300 K in multicanonical dynamic docking. Unlike static docking scores, the *R*-value quantifies the persistence of noncovalent contacts, including hydrogen bonds, salt bridges, van der Waals, and π–π stacking, across all intermolecular atom pairs within 4.5 Å, capturing the full spectrum of short-range interactions (hydrophobic contacts typically ≤4.0 Å) [76]. While not a direct measure of free energy, the *R*-value captures key enthalpic and conformational features and thus serves as a practical proxy for binding stability, particularly in HT-MD simulations that enhance sampling of native-like interaction networks. By combining contact frequency and longevity, the *R*-value distinguishes stable, near-native poses from unstable but geometrically similar decoys. Overall, this framework provides a systematic and quantitative approach for evaluating docking-derived complexes, particularly in systems lacking experimental structural data, and may support prioritization in ligand optimization workflows.

The observed correlation between *R*-value and experimental p*K*_d_ across multiple systems supports its use as a stability-based descriptor. While the per-residue *R*-value inherently weights contacts by count, it does not isolate each residue’s contribution to binding stability. We define *R*-value contribution as the per-residue *R*-value multiplied by contact count, enabling direct assessment of individual residues’ impact. This metric combines contact frequency and stability; residues with few contacts but high stability contribute less than those with many persistent interactions. Thus, *R*-value contribution reflects the collective stabilizing role of residue–ligand contact networks. Residues with strong correlations between *R*-value contribution and p*K*_d_ often map to catalytic or functional sites, e.g., Glu62 in KRAS^G12D^ (*r* = 0.99) and Asp29 in HIV-1pro (*r* = 0.88), supporting its potential utility in identifying key anchoring residues that govern affinity and stability. This provides a quantitative link between dynamic stability metrics and SAR analysis, suggesting that stability-guided ligand optimization may complement conventional quantitative SAR approaches. A key application may lie in the design of next-generation KRAS^G12D^ inhibitors, where MRTX1133, despite high potency, faces resistance mechanisms [77,78], including secondary variants (e.g., V9E/W/Q, G13P, and Y96W) and an unresolved phase 1/2 trial termination (NCT05737706) [79]. Our analysis suggest that Asp12, Gly60, Glu62, Asp69, and His95 as critical for MRTX1133 binding. Designing novel inhibitors that preserve interactions with these residues using alternative scaffolds may help maintain affinity while potentially improving resistance profiles.

Enhanced sampling techniques such as umbrella sampling [80], multicanonical MD [81], and replica-exchange MD [82] enable rigorous and exhaustive exploration of conformational space by accelerating biologically relevant motions through biasing potentials or parallel tempering [83,84]. In addition, rigorous RBFE approaches, including free-energy perturbation [85] and thermodynamic integration [86], represent state-of-the-art methodologies for quantitative affinity prediction within congeneric ligand series. These methods typically provide high quantitative accuracy but require substantial computational resources and are time intensive [87,88], limiting their routine application in early-stage or large-scale screening contexts. The presented HT-MD framework is designed as a stability-guided refinement approach rather than a direct free-energy prediction method. Although the use of 12 × 100-ns replicas represents a meaningful computational investment, each trajectory operates within a conventional short-time-scale MD regime without requiring enhanced sampling schemes or alchemical transformations [75], resulting in a comparatively simple and readily implementable workflow. Although HT-MD is conducted at nonphysiological temperatures, previous work by Bekker *et al.* demonstrated that contact-based stability metrics derived from 400 K simulations correlate strongly with experimental melting temperatures and have successfully guided thermostabilizing mutations in single-domain antibodies [30,31], thereby supporting the physical relevance of stability patterns captured under accelerated conditions.

While the *R*-value, derived from the full trajectory and intrinsically integrating temporal dynamics, does not fully capture entropic or solvent contributions typically addressed in more advanced and exhaustive simulations [89,90] and while simulations at elevated temperatures (e.g., 400 K) may not fully reproduce native thermodynamics or solvation effects, these limitations should be considered when interpreting the results. Nevertheless, the *R*-value provides a stability-centered and reproducible descriptor that is directly applicable to pose validation and ligand optimization. The consistent correlations observed between *R*-value and experimental p*K*_d_ across multiple systems further support its use as a practical, early-stage metric for guiding ligand design.

## Conclusions

Here, we present a computational workflow integrating docking, HT-MD simulations, and stability-driven *R*-value analysis to identify stable, native-like binding poses and map residue-level contact stability for rational ligand optimization. Results show that binding stability inversely correlates with pose similarity to the experimental structure: 83.9% of top *R*-value poses have an RMSD <3.0 Å. The framework identifies stable modes that preserve key nonbonded interactions (high *R*-value) even when the overall conformation differs from the experimental structure. The *R*-value shows correlations with experimental p*K*_d_ (0.40 to 0.94), supporting its use as a stability-based descriptor associated with binding affinity. To illustrate its potential application in ligand optimization, we performed a proof-of-concept analysis using *in silico* modifications of TH-Z835 and MRTX1133 targeting KRAS^G12D^. These exploratory examples demonstrate that modifying functional groups to enhance persistent contacts can improve interaction stability, as reflected by increased *R*-values, without requiring extensive free-energy calculations. This stability-driven approach offers a rapid, resource-efficient complement to thermodynamics-based methods. While performance varies by system (e.g., flexible or buried sites), it applies to any docking pose without prior native structure knowledge, highlighting its potential utility as a structure-agnostic prioritization strategy rather than a replacement for experimental validation or rigorous free-energy methods. Thus, stability-driven pose evaluation provides a scalable, structure-agnostic strategy for accelerating rational ligand design in protein–ligand systems.

## Materials and Methods

### Complex structure selections

The initial complex structures were obtained from the Protein Data Bank Japan web services [10,37] comprising 10 different protein systems, each containing a varying number of ligands, ranging from 6 to 30 ligands per protein. The list of PDB entries utilized in this work is detailed in Table 1 and Table S1.

### Molecular docking studies

The docking preparation and docking protocol were conducted using a modified scheme based on our previous study [91] using the MOE 2022.02 software [92,93]. Prior to docking, both proteins and ligands were prepared. Existing solvents, unnecessary ligands (e.g., sulfate and glycerol), and ions were removed. Additionally, the Amber10:EHT force field [94] was used to parameterize the protein. Finally, the default “LigX” protocol in MOE was employed. This protocol includes capping the N- and C-termini with ACE and NME groups, respectively, to avoid artificial charge effects arising from missing terminal residues, to neutralize any exposed charged groups and improve the physical realism of the system. Additionally, any missing loops or residues that were supposed to be present in the protein structure were also generated and modeled using MOE. For each ligand, the 3-dimensional structure was extracted from the original complex and prepared using the “Wash” protocol, including protonation at physiological pH 7.0 (except for HIV-1pro, where pH 5.0 was applied), with the most probable protonation state subsequently assigned. The ligands were then parameterized using the MMFF94x force field in “Gas Phase” solvation, followed by energy minimization with an RMS gradient of 0.001 kcal·mol^−1^·Å^−2^.

For ligands with experimentally determined structures (PDB-available; see the “*R*-value in binding pose prediction: The higher the *R*-value, the closer it is to native” section), template-free (unconstrained) cross-docking was performed, in which each bound ligand was extracted from its original crystal structure and redocked into a single reference receptor (Table 1). The “Site Finder” feature in MOE identified the ligand-binding pockets, with residues within 4.5 Å of the original ligand defined as the docking site. Docking used the “Induced Fit” protocol to allow limited side-chain flexibility, permitting movement only for residues selected by the “Site Finder”. This process generated thousands of initial binding poses per ligand, before selecting 300 nonduplicated configurations, from which the top 100 binding poses were retained based on the MOE docking score (approximation of Δ*G*_binding_). In the end, the best 10 docking poses with the lowest MOE docking score were stored for HT-MD analysis.

For ligand design studies (see the “Stability-guided, SAR-like analysis enables ligand optimization in KRAS^G12D^” section), template-based (constrained) docking was performed using TH-Z835 (PDB ID: 7EWB) [45] and MRTX1133 (PDB ID: 7RPZ) [44] as references: for TH-Z835, the heterocyclic ring and the [3.2.1] bicyclic diamino substituent were selected as the template; for MRTX1133, the entire structure except for the moiety connected to the C2 atom was used as the template (Fig. S16). Here, only the top-ranked pose from this template-guided docking was retained for subsequent HT-MD analysis, as the template constrains the ligand to the reference conformation and reduces sampling of alternative poses.

### Canonical MD simulations

In this study, a modified version [32] of the GROMACS 2024.04 software [95–97] was utilized to conduct the MD simulations, as well as performing tasks such as protein topology preparation, system box setup, addition of solvents/ions, execution of energy minimization, equilibration, and, ultimately, the production run of the protein–ligand systems. In addition, the Gaussian09 software [98] and AmberTools toolset were used to prepare and build the ligand topology parameters. The top 10 binding poses per ligand, obtained from the docking studies as described above, along with all protein–ligand complexes from crystallographic studies, were included in the high-temperature canonical MD simulations to assess the binding stability of the ligands in their respective binding sites.

The system preparation and MD simulation protocol follow the strategy established in our previous studies [34] but is explained in greater detail here. First, ligand geometries were optimized at the Hartree–Fock/6-31G* level [99] under implicit solvent conditions using the polarizable continuum model, and restrained electrostatic potential [100] fitting was performed using Antechamber to derive atomic partial charges consistent with the AMBER convention. Then, the AMBER99SB-ILDN force field [101] was utilized to parameterize the protein, while GAFF2 [102] was used to parameterize the ligand. The protein–ligand complex system was solvated in a triclinic box filled with TIP3P [103] water molecules, extending 9 Å beyond the protein on each side and subsequently neutralized with sodium (Na^+^) and chloride (Cl^−^) ions [104]. Afterward, 2 iterations of energy minimization using steepest descent criteria was employed to address any potential steric clashes that may have arisen earlier, where the first iteration used position restraints on the heavy solute atoms until a force of 1,000 kJ·mol^−1^·nm^−1^, while the second iteration was unrestrained and performed until a force of 100 kJ·mol^−1^·nm^−1^. Subsequently, NVT (constant-volume) and NPT (constant-pressure) equilibration MD simulations were performed for 100 ps at 300 K and 1 bar, to equilibrate the velocities and box size. To achieve a higher integration time during simulation, hydrogen mass repartitioning [105] was applied by transferring some of the mass from heavy atoms to their bound hydrogen atoms, allowing for efficient simulation without compromising stability. Additionally, a center-of-mass restraint was applied to the protein to prevent rotation and translation, allowing the use of a triclinic box to reduce system size and improve performance. Finally, a production run of 12 independent 100-ns MD simulations at 400 K with a timestep of 4 fs was performed, where for each trajectory the velocities were generated from a Maxwell distribution corresponding to 400 K.

### Representative structure selection

The representative structure for each protein–ligand system was defined as the snapshot whose intermolecular contact pattern most closely matches the ensemble average across the analyzed portion of the trajectory. For the MD simulation analysis, only the latter 40 ns of each trajectory is used, with the initial 60 ns treated as equilibration, consistent with our previous work [30,106]. For all snapshots in the analyzed portion of the trajectory, protein–ligand heavy-atom contact pairs were identified, and their average distances were computed across the trajectory. Only contact pairs with an average distance ≤6.75 Å were retained to construct the ensemble-averaged contact matrix. For each frame, the distances of the same retained protein–ligand heavy-atom pairs were assembled into a distance vector, and the Euclidean distance to the ensemble-averaged distance vector was computed. The frame with the minimum Euclidean distance to the ensemble-averaged contact matrix was selected as the representative structure. Full computational details and equations are provided in Section S1.

### Analysis of MD simulations

The analysis stage of the MD simulation results consists of 2 stages [106]. First, the representative structure for each system is determined as described in the “Representative structure selection” section. From this representative structure, we compute the *R*-value [32,33], RMSD, and RMSF to quantify ligand-binding stability and structural fluctuations, using the MDTraj software package [107] for *R*-value analysis. Additionally, the selected representative protein–ligand complex structures were visualized using UCSF ChimeraX 1.10 [108], while protein–ligand complex visualization with mapped electron density was performed using PyMOL 3.0 [109]. Full equations and implementation details are provided in Section S1.

*R*-value analysis was used to assess binding mode stability relative to the MD-derived ensemble, reflecting dynamic interaction persistence. The *R*-value is based on the *Q*-value [33], which is defined as

$$\begin{array}{c} R=\frac{1}{N}\sum_{\left(i,j\right)}\frac{1}{1+\exp\left(\beta \left(r_{\mathrm{ij}}\left(X\right)-\Lambda r_{\mathrm{ij}}^{0}\right)\right)} \end{array}$$ (1)

where *N* is the number of native contacts (distance < 4.5 Å), $r_{\mathrm{ij}}\left(X\right)$ is the interatomic distance of the pair (*i*, *j*) in configuration *X*, and $r_{\mathrm{ij}}^{0}$ is the corresponding (*i*, *j*) pair distance in the reference structure. The parameters *β* (smoothing parameter) and Λ (contact fluctuation parameter) were set to 5.0 Å^−1^ and 1.8, respectively.

RMSD analysis was performed to evaluate structural similarity between predicted and experimental binding poses. The RMSD of ligand heavy atoms was calculated after optimal superposition, using the experimental PDB structure as reference and the MD-derived representative structure as input. RMSD is defined as

$$\text{RMSD}=\sqrt{\frac{1}{N}\sum_{i=1}^{N}{\left(x_{i}-x_{i}^{\mathrm{ref}}\right)}^{2}}$$ (2)

where $x_{i}$ and $x_{i}^{\mathrm{ref}}$ denote the coordinates of atom *i* in the input and reference structures, respectively.

RMSF analysis was performed for protein Cα atoms over the analyzed trajectory. All frames were least-squares aligned to the representative structure using backbone (or Cα) atoms. The RMSF for each residue was calculated as

$${\text{RMSF}}_{i}=\sqrt{\frac{1}{T}\sum_{t=1}^{T}{\left(x_{i}\left(t\right)-\langle x_{i}\rangle \right)}^{2}}$$ (3)

where *x_i_*(*t*) is the position of the Cα atom of residue *i* at time *t*, ⟨*x_i_*⟩ is the time-averaged position of residue *i*, and *T* is the total number of frames.

## Data Availability

The source code for the modified version of GROMACS is available at https://gitlab.com/gjbekker/gromacs. The raw simulation data have been submitted to the Biological Structure Model Archive (BSM-Arc) [110], under BSM-00095 (https://doi.org/10.51093/bsm-00095).
