# Supplementary material for: Stability-Driven Pose Prediction and Ligand Design via Contact Persistence Analysis from Molecular Dynamics Simulations
Source: Comput Struct Biotechnol J. 2026 May 29;35(1):0093. doi: 10.34133/csbj.0093 (PMC13219992; doi:10.34133/csbj.0093)
Supplement: Supplementary 1 — Supplementary Text Figs. S1 to S16 Tables S1 to S6 [file csbj.0093.f1.docx]

# Supporting Information.

# SECTION

# Section S1: Methodology – Analysis of Canonical High-Temperature MD Simulations.

The analysis stage of the MD simulation results consists of two stages^1^. First is to determine a representative structure for each system, where the representative structure was defined as the snapshot whose intermolecular contact pattern most closely matches the ensemble average across the analyzed portion of the simulation. Subsequently, the representative structure is used as the reference structure during the MD simulation. For this analysis, only the latter 40 ns of each trajectory is used, where the initial 60 ns is used as equilibration, as in our previous works^1,2^.

The representative protein-ligand complex structure was defined as the snapshot whose intermolecular contact pattern most closely matches the ensemble average across the analyzed portion of the simulation. A contact matrix (C) was constructed to describe all pairwise heavy-atom distances between the protein and ligand, where each element *Cᵢⱼ* represents the distance (*dᵢⱼ*) between protein atom *i* and ligand atom *j* computed under the minimum-image convention (periodic boundaries). For tractability, we first applied a coarse prefilter on a 10-ns–subsampled trajectory, retaining the atom pairs that ever came within 9.0 Å; we then averaged the contact matrix over frames and fixed a pair list by keeping only those contacts whose average distance was ≤ 6.75 Å. For each frame *t*, these distances were assembled into a distance vector (*d^(t^*^)^), and the ensemble-averaged distance for each contact pair (*k*) was computed as:

$$\bar{d}_{k}=\frac{1}{N}\sum_{t=1}^{N} d_{k}^{(t)}$$

Equivalently, in matrix form,

$$\bar{C_{ij}}=\frac{1}{N}\sum_{t=1}^{N} C_{ij}^{(t)}$$

where *N* is the total number of analyzed frames. To quantify the similarity of each frame to this ensemble average, the Euclidean distance from the mean distance vector (*D(t)*) was computed as:

$$D\left( t \right)= \left\| d^{(t)}-\bar{d} \right\|_{2}= \sqrt{\sum_{k} {{(d}_{k}^{\left( t \right)}-\bar{d}_{k})}^{2}}$$

where the average distance vector is defined as $d$. The frame with the minimum Euclidean distance was designated as the representative structure, as it best reflects the dominant ligand-protein contact network observed throughout the simulation. This representative structure was subsequently used to define the final contact matrix, comprising all protein-ligand atom pairs within 4.5 Å (native contacts in the representative frame), which served as the reference for subsequent R-value and RMSD analyses. By describing all persistent heavy-atom interactions in this manner, the contact matrix captures both stable hydrogen bonds and transient hydrophobic or π-π contacts that together determine binding stability.

R-value analysis was performed as previously reported^3^, provided by MDTraj software package^4^.  The R-value is based on the Q-value^5^, which defined as:

$$Q=\frac{1}{N}\sum_{(i,j)} \frac{1}{1+exp(\beta(r_{ij}\left( X \right)-\Lambda r_{ij}^{0}))}$$

where *N* (also referred as contact counts) is the number of native contact pairs with a distance less than 4.5 Å, $r_{ij}\left( X \right)$ the distance of the pair (*i*, *j*) in configuration X, $r_{ij}^{0}$ the distance in the experimental configuration, $\beta$ a smoothing parameter and $\Lambda$ a parameter to account for the fluctuations formed by the contact, set to 5.0 Å^-1^ and 1.8 Å, respectively. The R-value, however, is designed to assess how contacts change with respect to the experimental structure during MD simulations. Hereby, the R-value generalizes the original Q-value in two key ways: (1) any reference structure, such as an ensemble average or simulation snapshot, can be used, not just the experimental structure; and (2) any subset of contacts (e.g., protein-ligand interactions) can be selected, not just the full protein structure. The reference structure used here is the representative structure calculated above, which corresponds to the ensemble center, providing a realistic, solution-phase benchmark of the stability of the protein-ligand interactions. The resulting value ranges from 0.0 to 1.0, where a value of 1.0 indicates full preservation of interactions between selected atom pairs (e.g., protein-ligand). Therefore, R-value analysis evaluates binding mode stability relative to the MD-derived ensemble, reflecting the dynamic behavior of the system.

Here, we use five R-value-based quantities: R(400 K, repr) (mentioned as R-value in the Section 2.2 to 2.4), R(400 K, exp), R(400 K, dock), the per-residue R-value ($R_{\text{res}}(k)$), and the R-value contribution. For any reference structure $X_{\text{ref}}\in\{X_{\text{repr}},X_{\text{exp}},X_{\text{dock}}\}$, the R-value over the selected set of protein–ligand native contacts $N_{LIG}$ is defined as:

$$R(X{}_{\mathrm{ref}})=\frac{1}{N_{LIG}}\sum_{(i,j)} \frac{1}{1+exp(\beta(r_{ij}\left( X{}_{\mathrm{ref}} \right)-\Lambda r_{ij}^{0}))}$$

where $N_{LIG}$ is the number of native protein–ligand atom pairs within the defined 4.5 Å cutoff. Here, the first three R-value variants differ only by the reference structure: R(400 K, repr) uses $X_{\text{ref}}=X_{\text{repr}}$(400 K MD-derived representative structure), R(400 K, exp) uses $X_{\text{ref}}=X_{\text{exp}}$(experimental structure), and R(400 K, dock) uses $X_{\text{ref}}=X_{\text{dock}}$(docking structure). The per-residue R-value for residue *k* averages the same contact score over the ligand atom corresponds to the residue *k* ($N_{res,k}$), defined as:

$$R_{res}\left( k \right)=\frac{1}{N_{res,k}}\sum_{\left( i,j \right)\in N_{res,k}} \frac{1}{1+\exp\left( \beta\left( r_{ij}\left( X_{\text{repr}} \right)-\Lambda r_{ij}^{0} \right) \right)}$$

where $N_{(\text{res},k)}$ is the number of native protein–ligand atom pairs in which the protein atom belongs to residue $k$. Finally, per-residue R-value contributions calculates the unnormalized, raw score of per-residue R-value for residue *k*, defined as:

$$R_{Contribution, res}\left( k \right)=N_{res,k}\times R_{res}\left( k \right)=\sum_{\left( i,j \right)\in N_{res,k}} \frac{1}{1+\exp\left( \beta\left( r_{ij}\left( X_{\text{repr}} \right)-\Lambda r_{ij}^{0} \right) \right)}$$

this per-residue R-value contributions can be used to calculate %Contribution, obtained by normalizing this raw score by the unnormalized R-value, which equals $R(X_{\text{repr}})\times N_{\text{LIG}}$,

$${\%R}_{Contribution, res}\left( k \right)= 100 x \frac{R_{Contribution, res}\left( k \right)}{R\left( X{}_{\mathrm{repr}} \right) N_{LIG}}$$

RMSD analysis was conducted to evaluate structural similarity between the input and reference structures by computing the root-mean-square deviation of the ligand’s heavy-atom coordinates after optimal superposition. In this study, the original experimental structure from the PDB of each complex was used as the reference structure, while the representative structure obtained from the 400 K 100-ns MD studies were selected as the input structure, thereby calculating the similarity of the ensemble average with the experimental PDB structure. The general RMSD equation is defined as follows:

$$RMSD= \sqrt{\frac{1}{N}\sum_{i=1}^{N} {(x_{i}-x_{i}^{ref})}^{2}}$$

In this equation, x_i_ denotes the coordinates of the i-th atom in the input structure, while x_i_^ref^ denotes the coordinates of the same atom in the reference structure for comparison. Thereby, RMSD analysis evaluates how closely the predicted pose matches the experimental structure, which is a direct measure of structural accuracy.

RMSF analysis was performed for protein Cα atoms over the analyzed trajectory. All frames were least-squares aligned to the representative structure using backbone (or Cα) atoms. The RMSF for each residue was calculated as:

$$\mathrm{RMSF}_{i}= \sqrt{\frac{1}{T}\sum_{t=1}^{T} {(x_{i}(t)-\left\langle x_{i} \right\rangle)}^{2}}$$

where x*_i_*(t) is the position of the Cα atom of residue *i* at time *t*, ⟨x_i_⟩ is the time-averaged position of residue *i*, and *T* is the total number of frames. We evaluated whether the trajectory RMSF profile for the selected pose (the highest-R-value complex, or for PLpro, the highest-R-value yet dissimilar pose) and a pseudo-apo trajectory (generated by removing the ligand), to assess the impact of ligand binding on local flexibility.

# Section S2: Results – R-value Unveils Possible Alternative Binding Configurations.

Beyond the majority of ligands that are located in the SS region, we also examined outliers that fell into the SD, US, or UD regions (see R-value/RMSD threshold determinations; **Fig. 2a**). Of particular interest were ligands in the SD region, where poses remained highly stable yet deviated from their experimental references (20 poses, 14.5%; **Fig. 2b**). We here focus on two such systems: SARS-CoV-2 papain-like protease (PLpro), where the HT-MD simulations suggest alternative native conformations, and ER Glucosidase I (ERGluI), which exhibited the highest proportion of outliers arising in both docking- and native-initiated simulations. In both cases, we employed residue-level R-value and root-mean-square fluctuation (RMSF) analyses to identify the structural basis of ligand stability or instability under HT-MD simulations. Together, these two systems illustrate that SD outliers may reflect experimentally supported binding heterogeneity (PLpro) or apparent alternatives driven by weaker non-polar contacts and crystallization artifacts (ERGluI).

*2.1. SARS-CoV-2 PLpro.* Among the 14 non-covalent PLpro inhibitors analyzed, seven adopted stable yet dissimilar conformations relative to the experimental binding pose, placing them in the SD region. Across these ligands, the mean R-value was 0.98 ± 0.01 while the mean RMSD was 2.9 ± 1.6 Å. This unusual combination indicates that the outcome was not merely an artifact of docking limitations but instead reflects alternative binding modes worthy of further investigation. These inhibitors were previously designed to target the BL2 groove and ubiquitin-binding site,^6–8^ and share a common scaffold with two functional groups (R1 and R2) attached to the phenyl core. R1 was intended to engage the BL2 pocket, while R2 interacts with the ubiquitin site, where tertiary amines further reinforce binding through hydrogen bonding and/or ionic interactions with Glu167 **(Fig. S5a**)^9^. In our HT-MD simulations at 400 K, several inhibitors exhibited an inversion of the designed binding mode; R1 migrated toward the ubiquitin-binding site, while R2 engaged the BL2 pocket. In this swapped configuration, R2 contacted residues near the ubiquitin site (Arg166, Met208, Pro247), whereas R1 interacted with residues surrounding the BL2 groove (Pro248, Tyr264, Asn267) (**Fig. S5b**). Despite these differences, all poses shared a conserved feature: the central aromatic ring consistently anchored to Tyr268 via hydrophobic and π-π interactions, providing a core stabilizing element. RMSF analysis further confirmed Tyr268 as a persistent stabilizer in both the docking- and the experimental structure-initiated trajectories, whereas the apo PLpro structure showed pronounced Tyr268 fluctuations in the absence of ligand stabilization (**Fig. S5c**).

To compare the native-like and non-native-like docking poses, we performed RMSF (**Fig. S5c**) and residue-level R-value analyses (**Fig. S5d**), which showed broadly consistent stability across residues, with Arg166 being the only residue to exhibit a notable ΔR (defined as the difference in per-residue R-value between native-like and non-native-like poses) of ~0.10. This difference likely reflects orientation changes of the R1 and R2 substituents during simulation, but overall stability was not substantially affected. RMSF profiles likewise showed no major differences; the dynamics of predicted native-like and non-native-like docking poses overlapped closely with each other and with native-initiated trajectories, particularly across binding-site residues (Leu162-Thr301), indicating similar protein flexibility in all cases. The plausibility of these alternative binding modes is further supported by experimental evidence. Electron density (ED) maps of eight PLpro inhibitors (**Fig. S6**) indicate that the positions of R1 and R2 cannot be distinguished unambiguously at the available resolution (>2.5 Å). Moreover, the Jun12129 structure (PDB ID: 8UUY, 3.05 Å resolution) contains two resolved conformations in which R1 and R2 appear swapped (**Fig. S5e**). Although this may in part reflect the limited resolution of the crystal, it nevertheless supports the possibility of alternative binding orientations.

*2.2. ER Glucosidase I.* We next investigated the ER Glucosidase I (ERGluI) system, which contained the highest proportion of outliers across all targets, with 10 of 30 ligands (33.3%) classified as non-SS (five initiated from docking and five from experimental structure; **Fig. S7a**). Notably, the experimental pose of EB-0334 fell in the US quadrant while its docked pose remained in SS, whereas EB-0159 showed the opposite pattern, with the docked pose in the UD quadrant despite the experimental pose being SS. To compare these discrepancies, we superposed the experimental and docked poses before and after HT-MD simulations (**Fig. S7b**). For EB-0159, the docked pose bent its hydrophobic tail toward Arg419 rather than extending toward Glu474 as in the experimental structure. This misorientation disrupted π-π and π-alkyl contacts with Phe417 and Phe475 (+2 and +3 subsites) and failed to reproduce key glucose-binding domain (GBD) interactions via the valiolamine ring, particularly with Trp423 and Glu783^10^. The canonical GBD interactions also failed to form during HT-MDS, suggesting that if these contacts were absent in the initial docking pose, they were unlikely to be established within the simulated timescale. As a result, the docking-derived pose of EB-0159 was unstable under HT-MDS. These observations highlight a limitation of docking for ERGluI, where candidate poses frequently fail to capture the GBD interactions required for stable binding. EB-0334. In both docked and experimental poses, the hydrophobic substituent deviated from the +2/+3 subsites and instead engaged Arg394 and Tyr482. In the experimental pose, instability of Phe416 is observed, highlighting that even experimentally derived conformations may undergo destabilization under HT-MD simulations if the critical GBD contacts are absent.

To better understand the interaction patterns involving ERGluI, we performed residue-level R-value analysis across all 30 ERGluI inhibitors (**Fig. S7c**). Ligands initiated from experimental structures showed the highest RMSD of any system (mean 2.23 Å), reflecting greater conformational fluctuations. Stability was consistently concentrated in the non-catalytic GBD residues (Phe417, Phe421, Trp423, Asp424), while the catalytic nucleophile Asp586 and acid/base Glu783 were less stable but still contributed substantially. Together, GBD residues accounted for >40% of average R-value contributions. GBD is slightly buried and most of the interactions are hydrogen bonds, highlighting the critical role of these residues in maintaining ligand stability. In contrast, residues of the +2/+3 subsites (e.g., Phe416, Pro472, Phe475, Tyr725) contributed less, consistent with their reliance on weaker π-π and π-alkyl contacts that were more frequently disrupted during simulation. This explains why conformational changes beyond the hydrophobic linker were common and led to altered interaction patterns, as seen in EB-0334. Unlike PLpro, there is no experimental evidence supporting alternative binding modes in ERGluI. For example, EB-0484 exhibited a representative HT-MD simulations pose that was inconsistent with its ED map, particularly in the distal hydrophobic region (**Fig. S7d**). Similar mismatches across other SD ligands may reflect crystal packing effects that artificially stabilize certain conformations during crystallization^11,12^. As a result, under HT-MD simulations ligands may adopt alternative configurations not captured in the experimental structures, yielding stable poses with relatively high dissimilarity to the reference. While many such cases likely arise from weaker π-mediated contacts at the +2/+3 subsites or from crystal packing artifacts, the possibility that genuine alternative poses exist cannot be excluded given the unique characteristics of the ERGluI binding site.

**FIGURES**

**
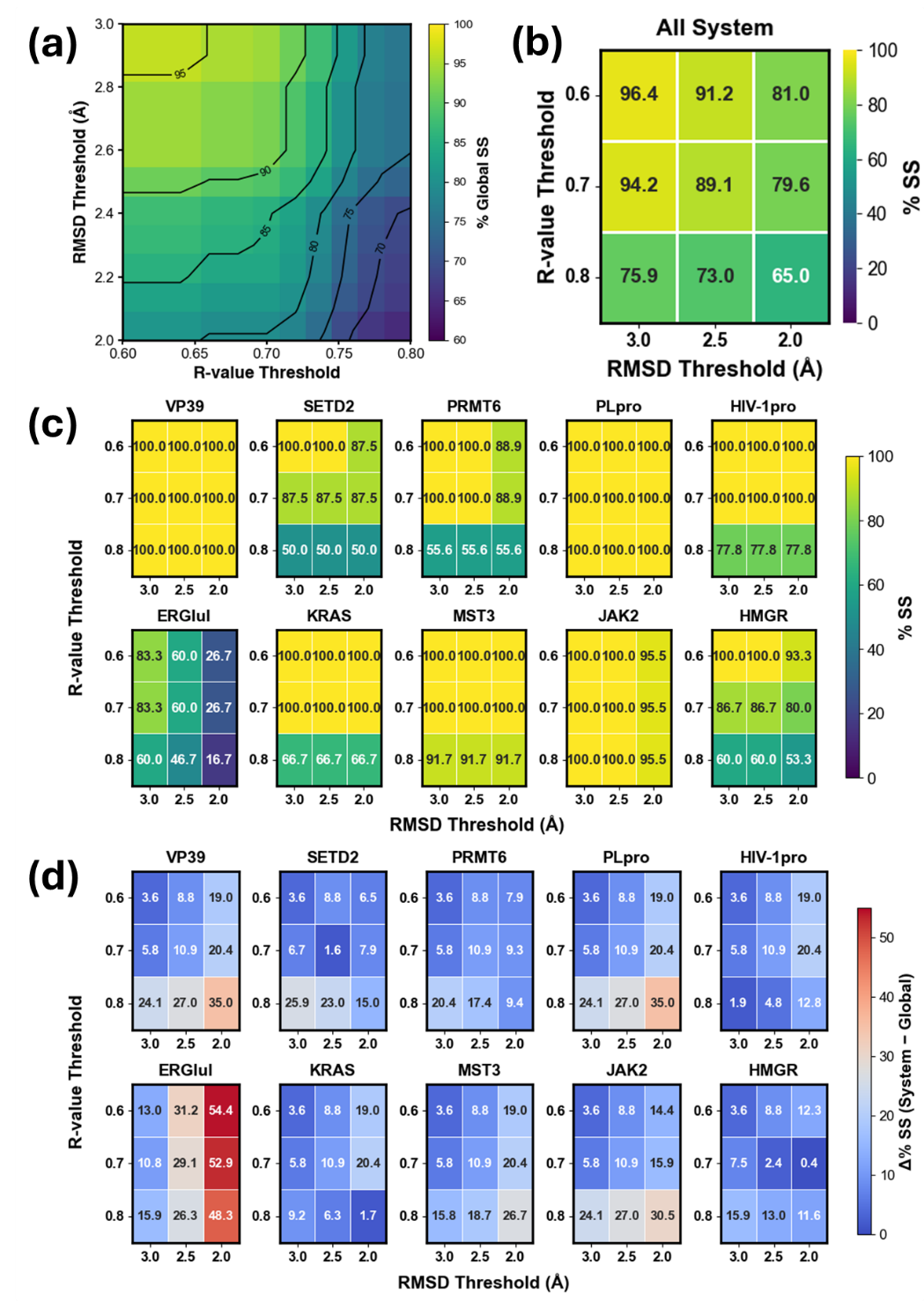
**

**Fig. S1. Global and system-level sensitivity of %SS and Δ%SS to R-value and RMSD thresholds.** (a) Two-dimensional heatmap showing the percentage of Global SS across continuous R-value (0.60–0.80) and RMSD (2.0–3.0 Å) thresholds. Black contour lines indicate 5% SS intervals. (b) Discrete matrix representation of Global SS (%) and (c) Percentage of System-specific SS (%SS) and (d) Δ%SS (%) matrices for individual targets at selected threshold combinations (R = 0.6–0.8; RMSD = 2.0–3.0 Å). Values within each cell denote the percentage of SS retained at the corresponding R-value (rows) and RMSD (columns) thresholds.


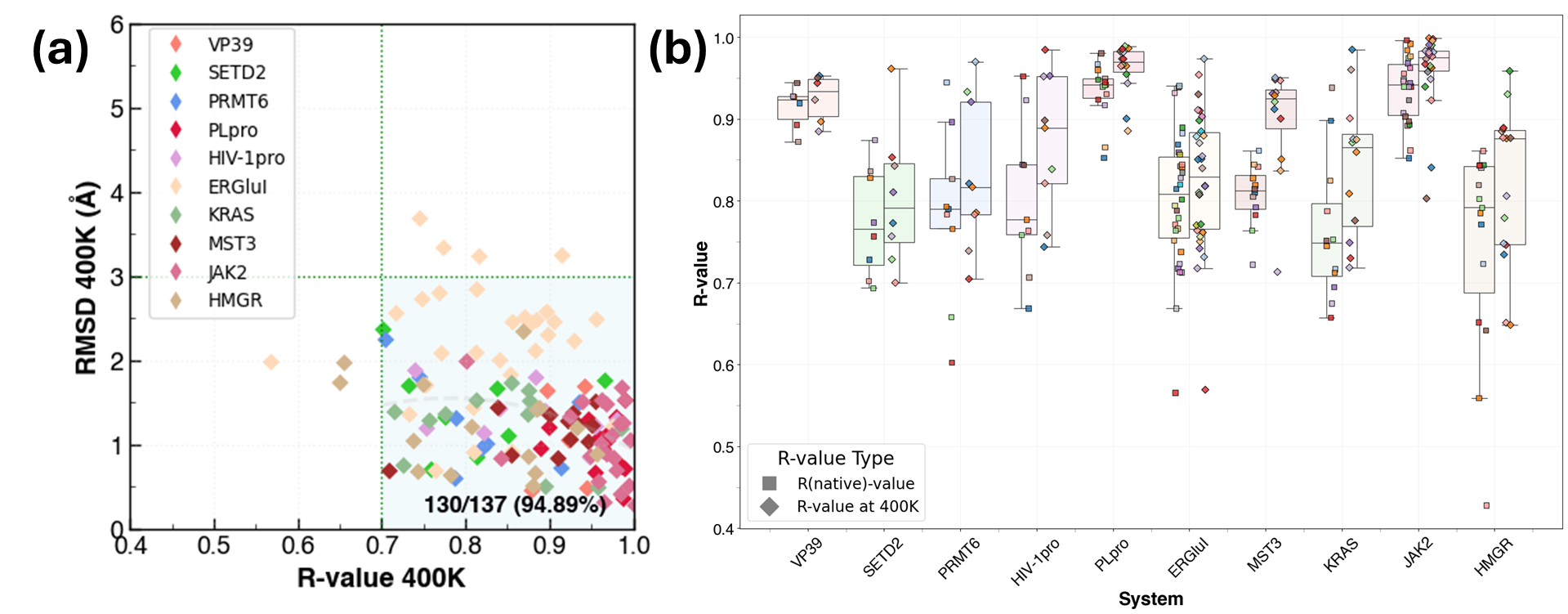


**Fig. S2. (a) 400K MDS results of the protein-ligand complexes started from the experimental structure,** based on the R-value score, with the corresponding RMSD calculated using the native structure as a reference. **(b) Per-system comparison of R(native)-values (circles), representing native contact similarity between the crystal structure and the 400 K MD-derived representative pose, and R-values after 400 K MD simulations (diamonds),** evaluated against the final 40 ns ensemble from twelve independent trajectories. Box plots and overlaid points illustrate the distribution and variability of native contact retention across systems


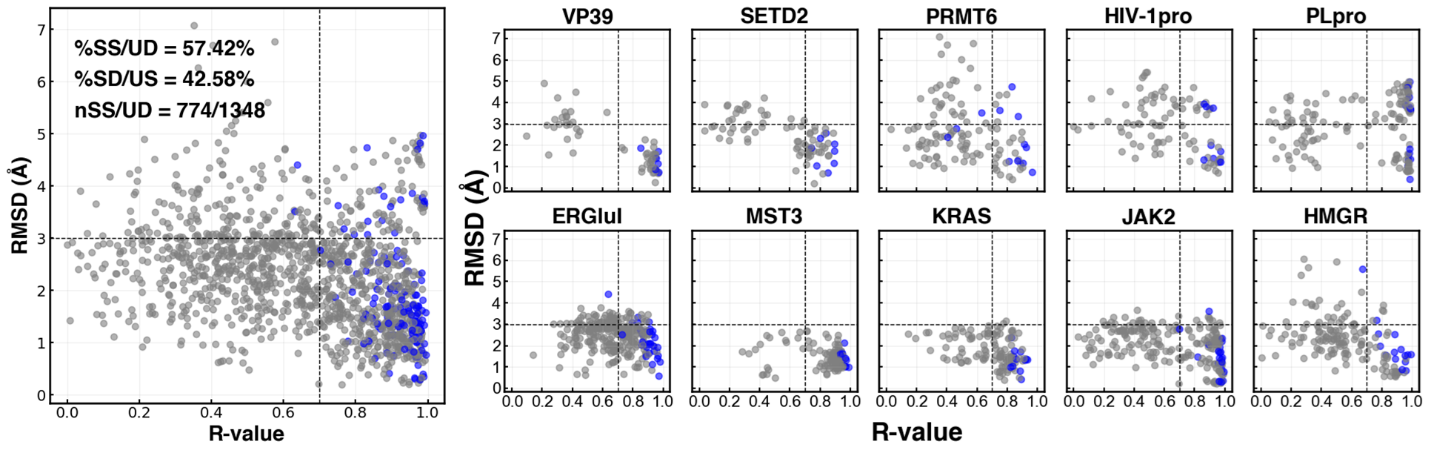


**Fig. S3. Quadrant classification of all binding poses across systems, based on R-value and RMSD to the native structure.** Dots are color-coded by docking rank: dark blue (Top 1), and gray (non-Top 1 poses). Dashed lines at R = 0.7 and RMSD = 3 Å define the boundaries for four categories—SS, SD, US, and UD—as described in **Fig. 2a**. System-specific quadrant plots show the distribution of poses for each target.


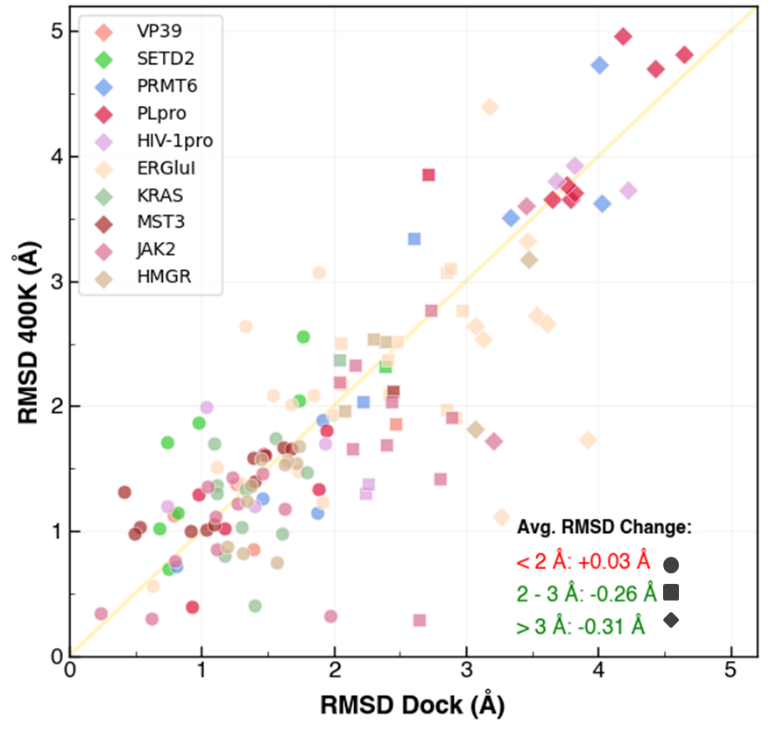


**Fig. S4. Average change in ligand heavy-atom RMSD between the docking pose and the 400 K MD–derived pose.** Colors correspond to target systems; markers denote initial docking RMSD bins: circles < 2 Å, squares 2–3 Å, diamonds > 3 Å.


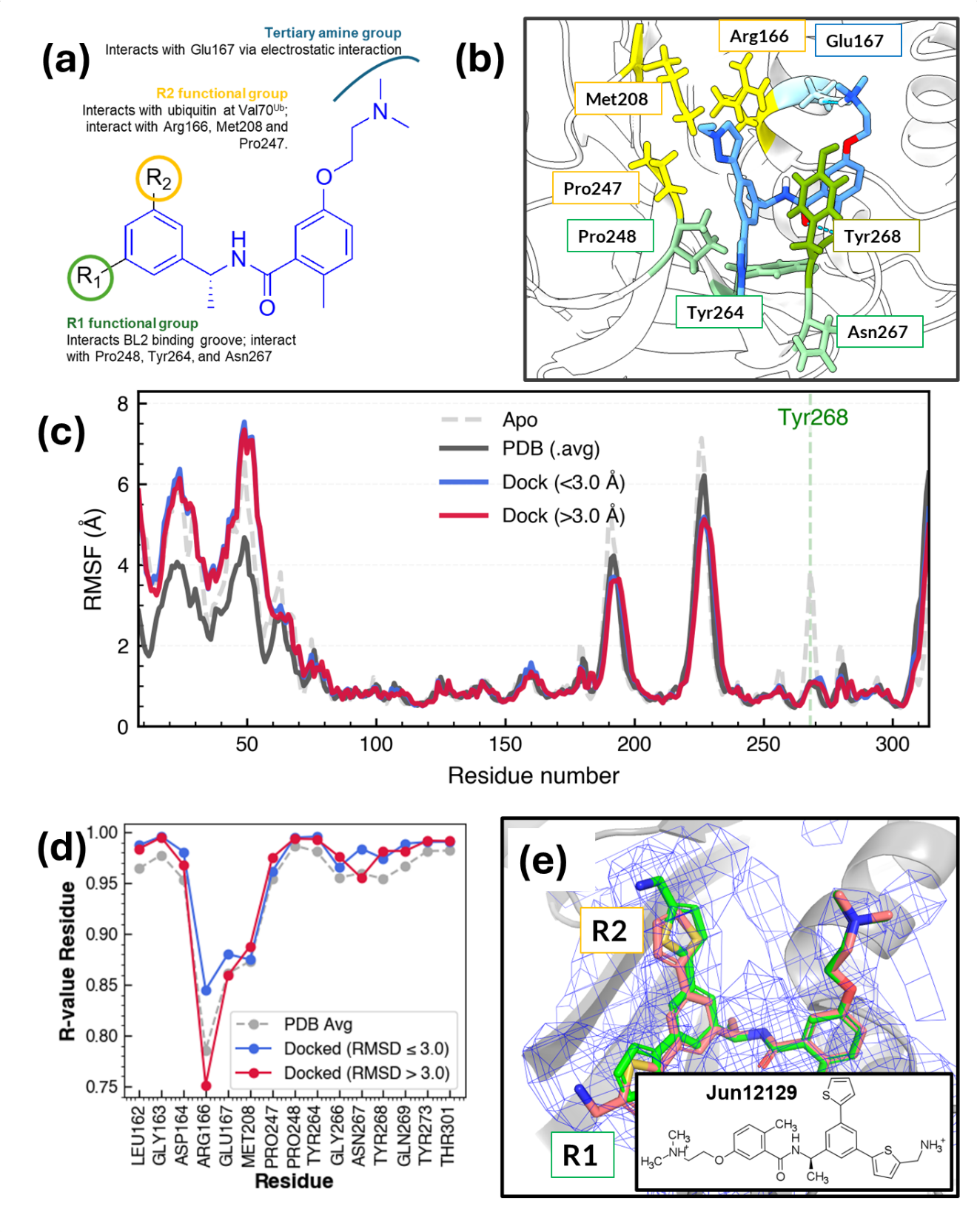


**Fig. S5.** **Structural and dynamic analysis of PLpro ligands reveals alternative binding configurations** (a) Molecular structure of biarylphenyl PLpro inhibitors indicating the R1 and R2 functional groups and the tertiary amine group. (b) Molecular visualization of the PLpro binding site in the presence of the ligand (depicted in light blue), with residues in proximity to the R1 and R2 groups highlighted (Arg166, Glu167, Met208, Pro247, Pro248, Tyr264, Asn267, Tyr268). (c) Per-residue RMSF (nm) of PLpro Cα atoms. Showing average of apo (light gray, dashed), PDB (dark gray, solid), native-like dock pose (RMSD<3.0 A, blue), and non-native-like dock pose (RMSD>3.0 A, red); Tyr268 indicated (green, dashed). (d) Per-residue R-value of PLpro ligands initiated from experimental structures (grey), stable and similar docked structures (blue), and stable but dissimilar docked structures (red). (e) Electron density (ED) map of PLpro with Jun12129 ligand, highlighting the resolved R1 and R2 substituents in the crystal structure.

**
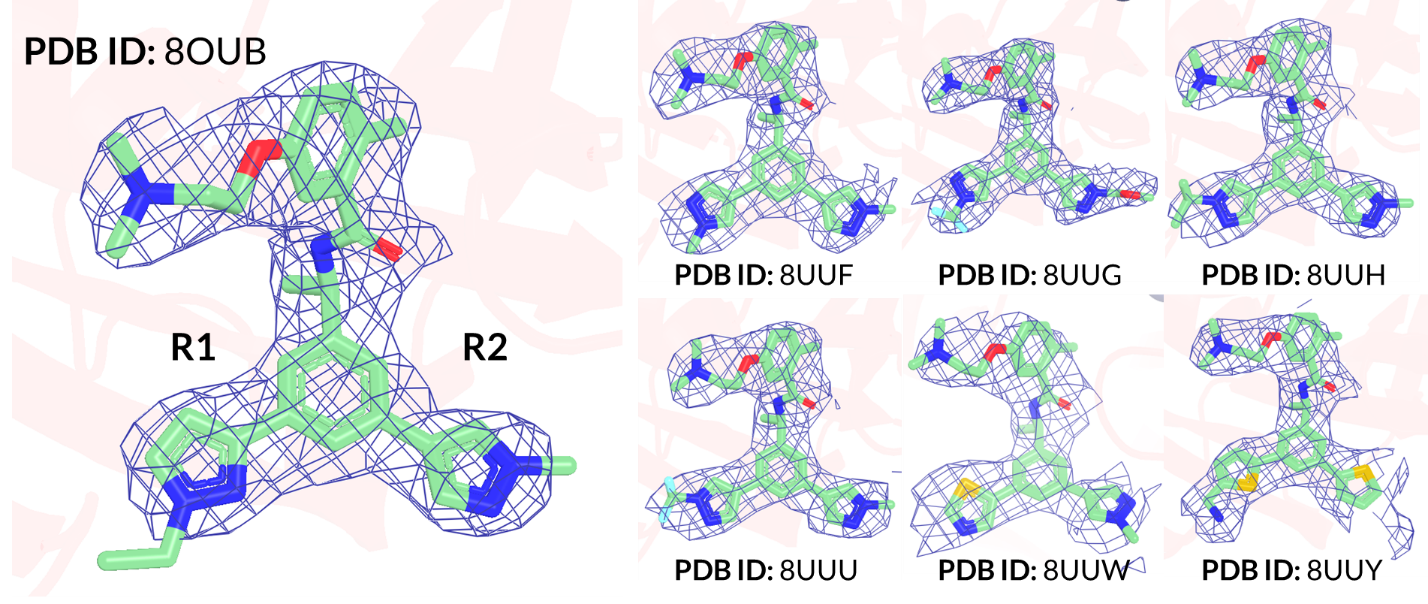
**

**Fig. S6. The electron density map of eight PLpro inhibitors.** The electron density map confirms that the R1 and R2 positions can be swapped due to the similarity in chemical structure, further supporting the possibility of alternate positioning. Moreover, Both Jun12129 and Jun12145, as shown in their crystal structures, indicate that an alternate conformation exists.


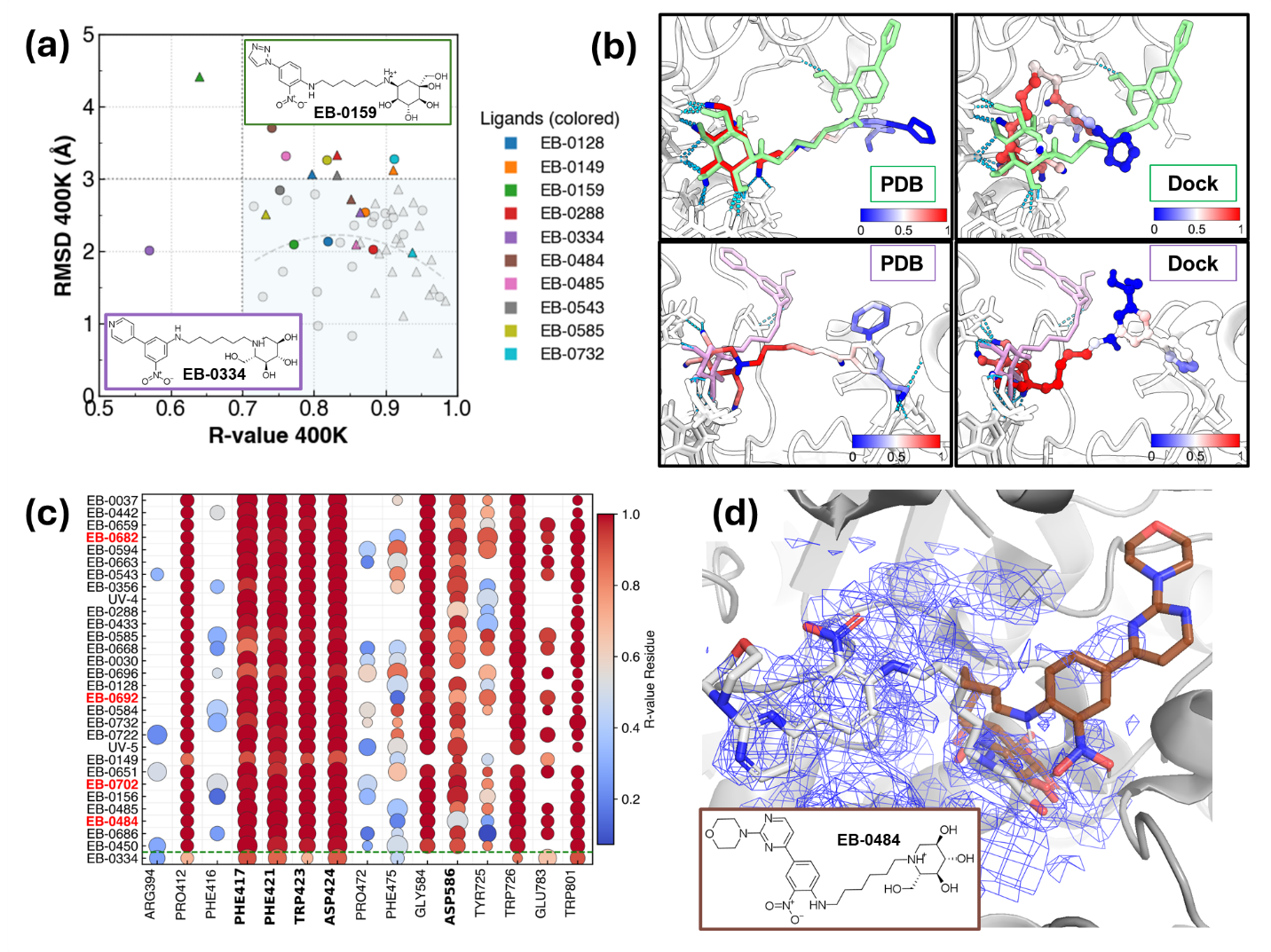


**Fig. S7. Structural and stability analysis of ER Glucosidase I (ERGluI) ligands.** (a) Correlation between R-value at 400 K and RMSD for PDB-initiated poses (circles) and most stable docked poses (triangles) obtained from HT-MD simulations. Ligands with at least one non-SS pose are colored; others are shown in gray. (b) Representative examples of EB-0159 (top) and EB-0334 (bottom), comparing PDB and docking poses with residue contact stability mapped by R-value (blue to red gradient, 0–1.0). The original crystal poses of EB-0159 and EB-0334 are highlighted in green and violet, respectively. (c) Heat map of residue-wise R-value contributions across native-initiated ERGluI ligands. Bold-labeled residues on the x-axis denote the glucose-binding domain (GBD). Red-labeled ligand indicates the ligand with high dissimilarity with respect of its native pose (RMSD > 3.0 Å) (d) Experimental electron density (2mFo–DFc map contoured at 1.0σ, blue mesh) for EB-0484. The inset shows the 2D structure of EB-0484.


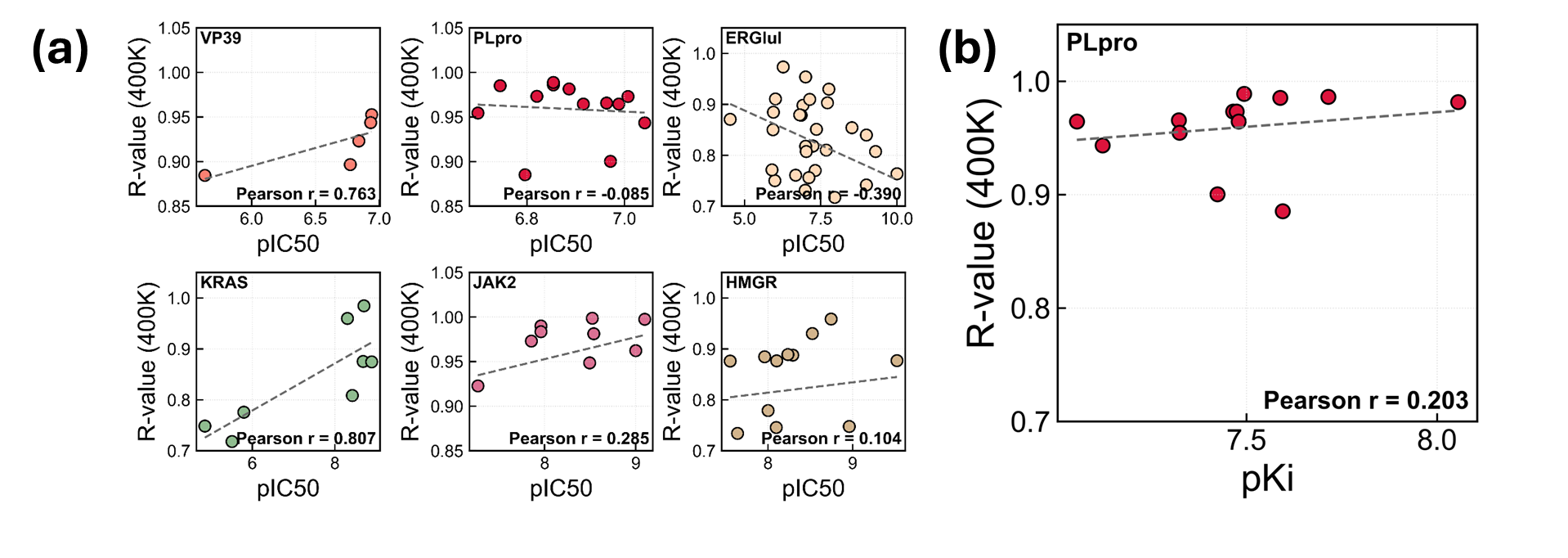


**Fig. S8. Correlation between (a) experimental pIC₅₀ and (b) pK_i_ (for PLpro) and MD-derived R-value across six protein systems (VP39, PLpro, ERGluI, KRAS, JAK2, and HMGR).** Each point representing a ligand. Dashed lines indicate linear regression fits, and Pearson correlation coefficients are shown in each panel.


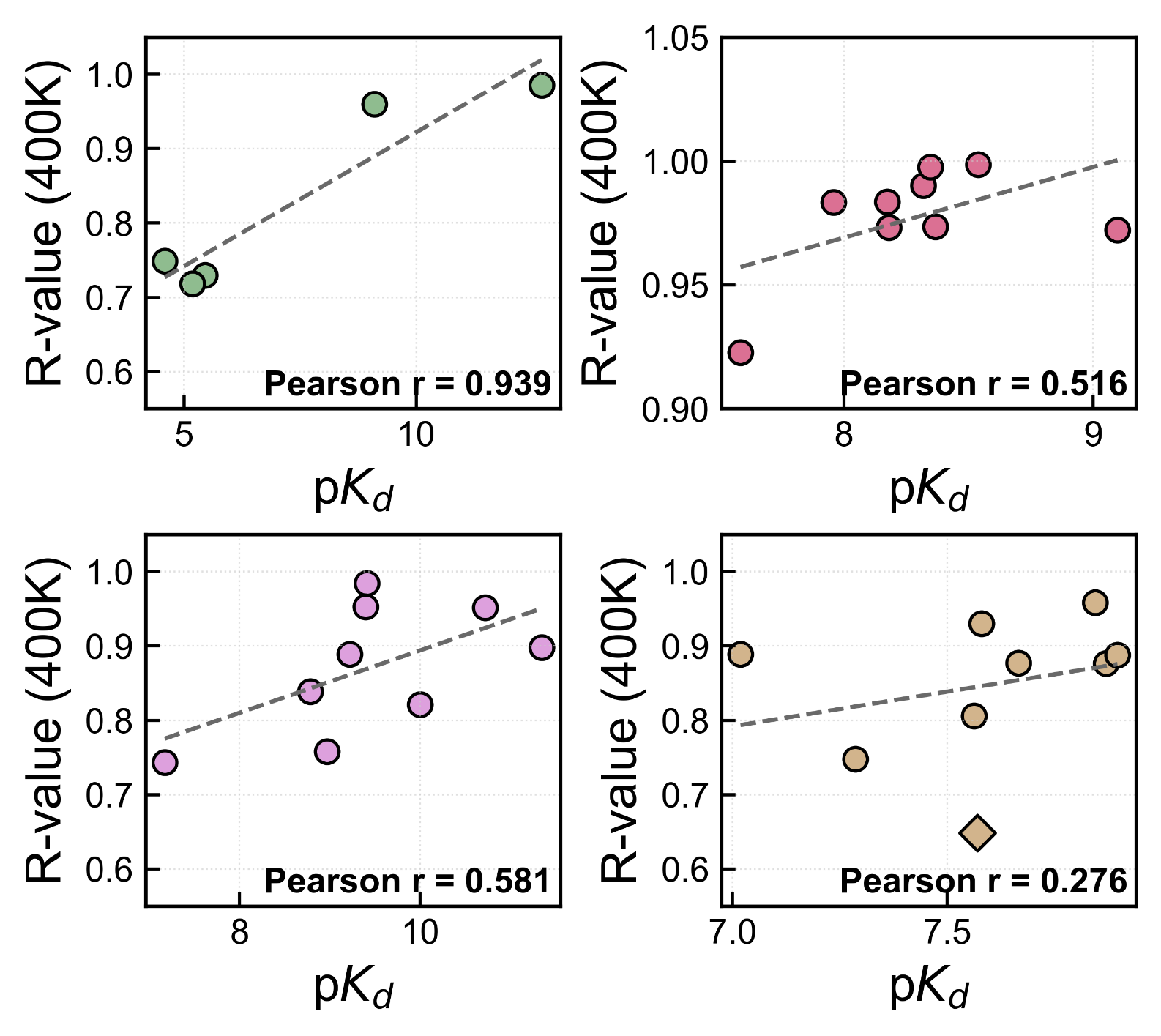


**Fig. S9. Correlation between R-value and experimental binding affinity (p*K*_d_) for HMGR.** Compound 11, excluded from **Fig. 3**, is shown as a diamond marker**.**


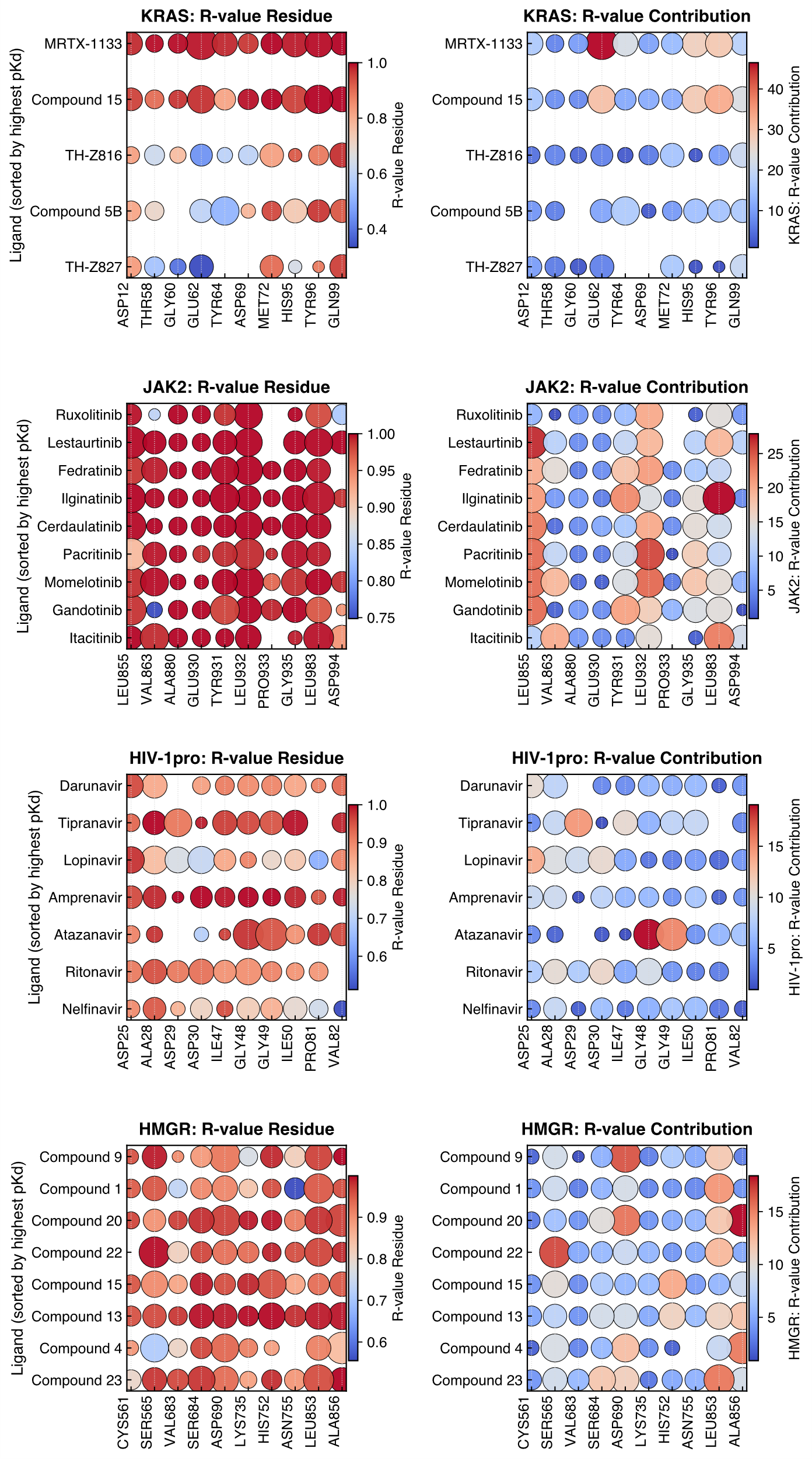


**Fig. S10. Per-residue and R-value contribution maps for representative ligand sets across four systems (KRAS^G12D^, JAK2, HIV-1pro, and HMGR).** Top panels show residue-level contact stability (R-value), while bottom panels indicate weighted R-values combining stability and contact frequency. Circle size ∝ contact count; color represents stability (blue = low, red = high). Ligand are sorted based on the highest p*K*d.


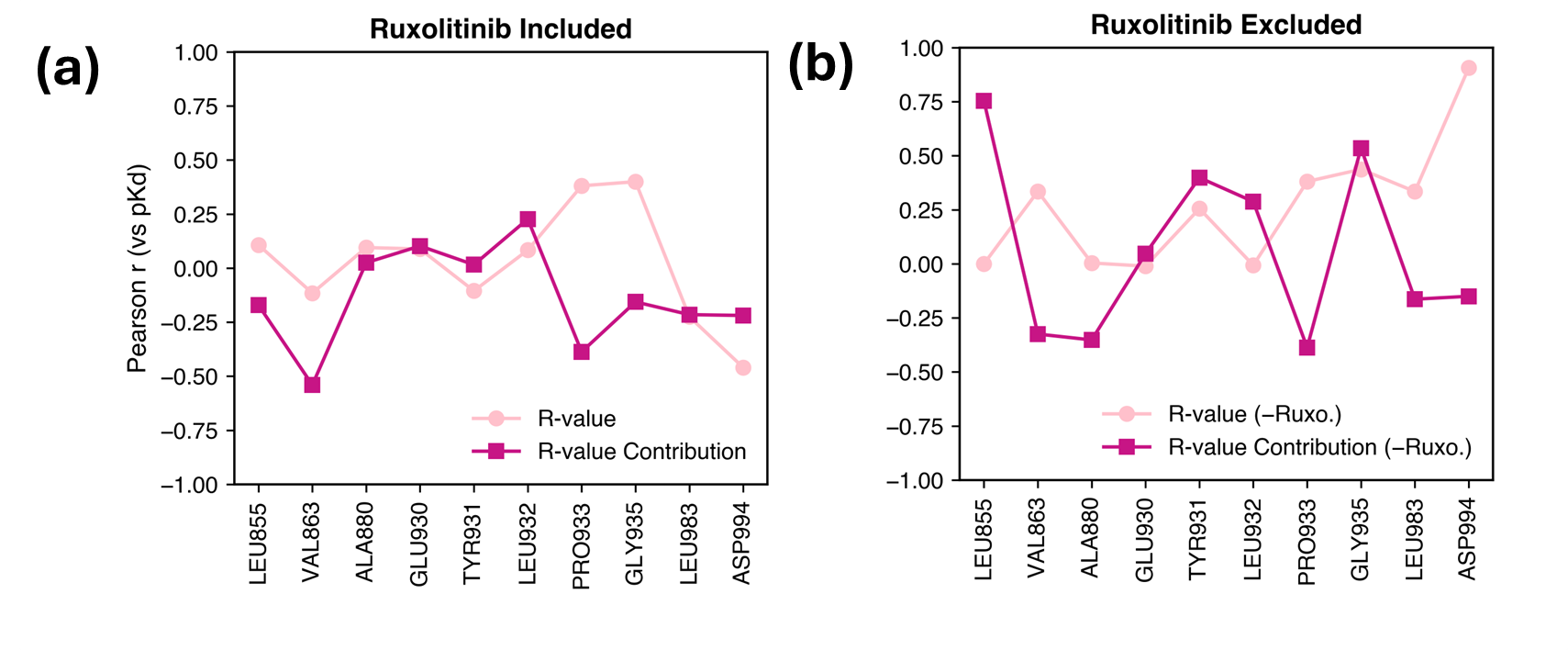


**Fig. S11. Per-residue correlation of R-value and R-value contribution with experimental p*K*_d_ for JAK2 inhibitors.** (a) Correlation including all ligands. (b) Correlation after excluding ruxolitinib, whose removal improves overall correlation and highlights key affinity-determining residues.


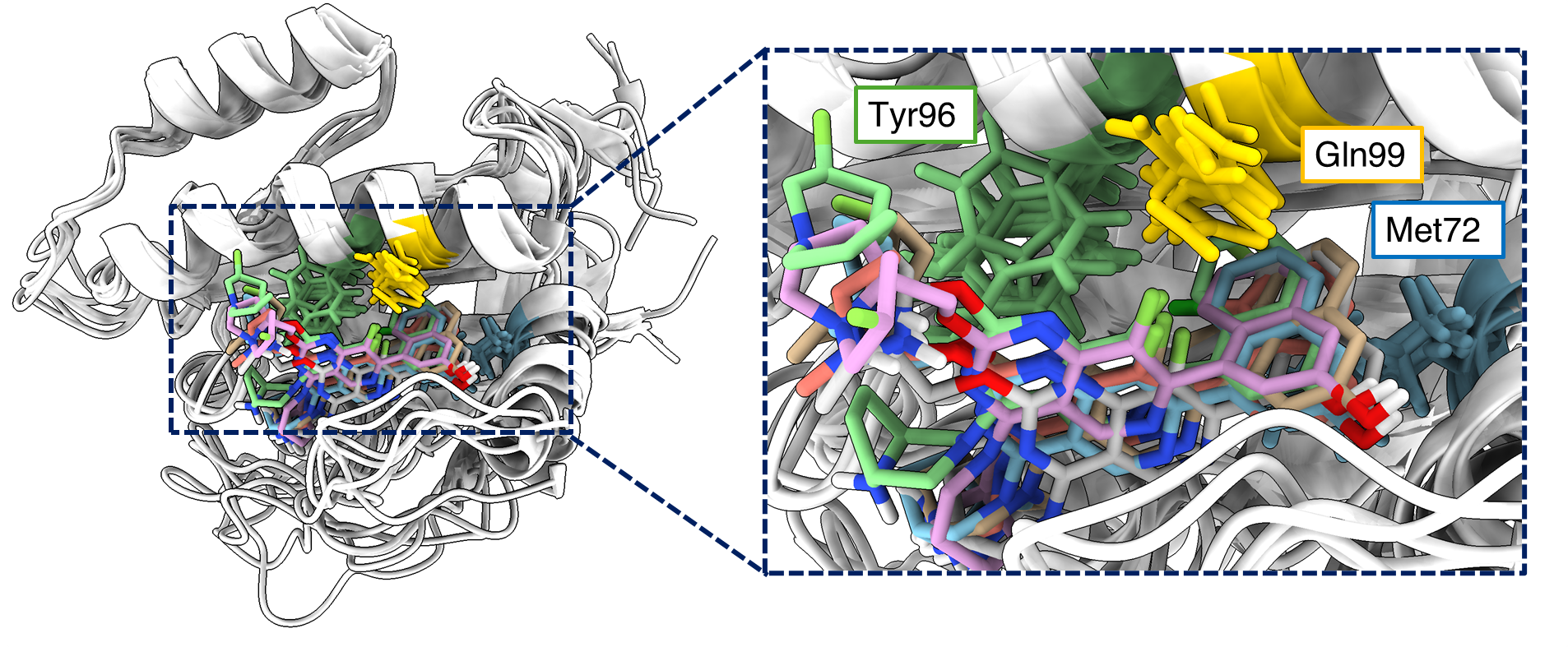


**Fig. S12. Superposition of representative KRAS^G12D^–ligand complexes (n = 6).** The inset highlights the binding site, including Gln99 (yellow), Met72 (blue), and Tyr96 (green). No hydrogen bond interactions involving these residues are observed in the complexes.


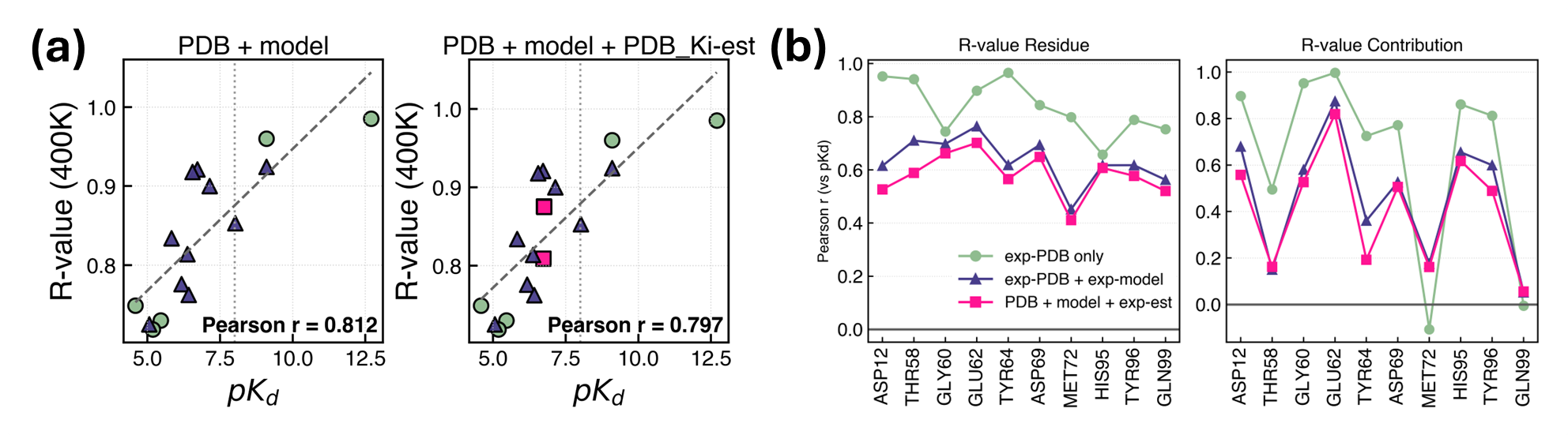


**Fig. S13. Stability metrics track experimental affinity across KRAS^G12D^ ligand evidence sets** (a) Correlation of R-values at 400 K with experimental binding affinities shows strong inverse relationships, with modeled structures generated by template-based docking represented as blue triangles and PDB-derived structures with *K*_d_ values converted from IC₅₀ using a regression model shown as violet squares. (b) Per-residue Pearson correlations of R-value Residue (left) and R-value Contribution (right) with ${pK}_{d}$ across top-10 most contributed residues in ligand stabilization across ligands.


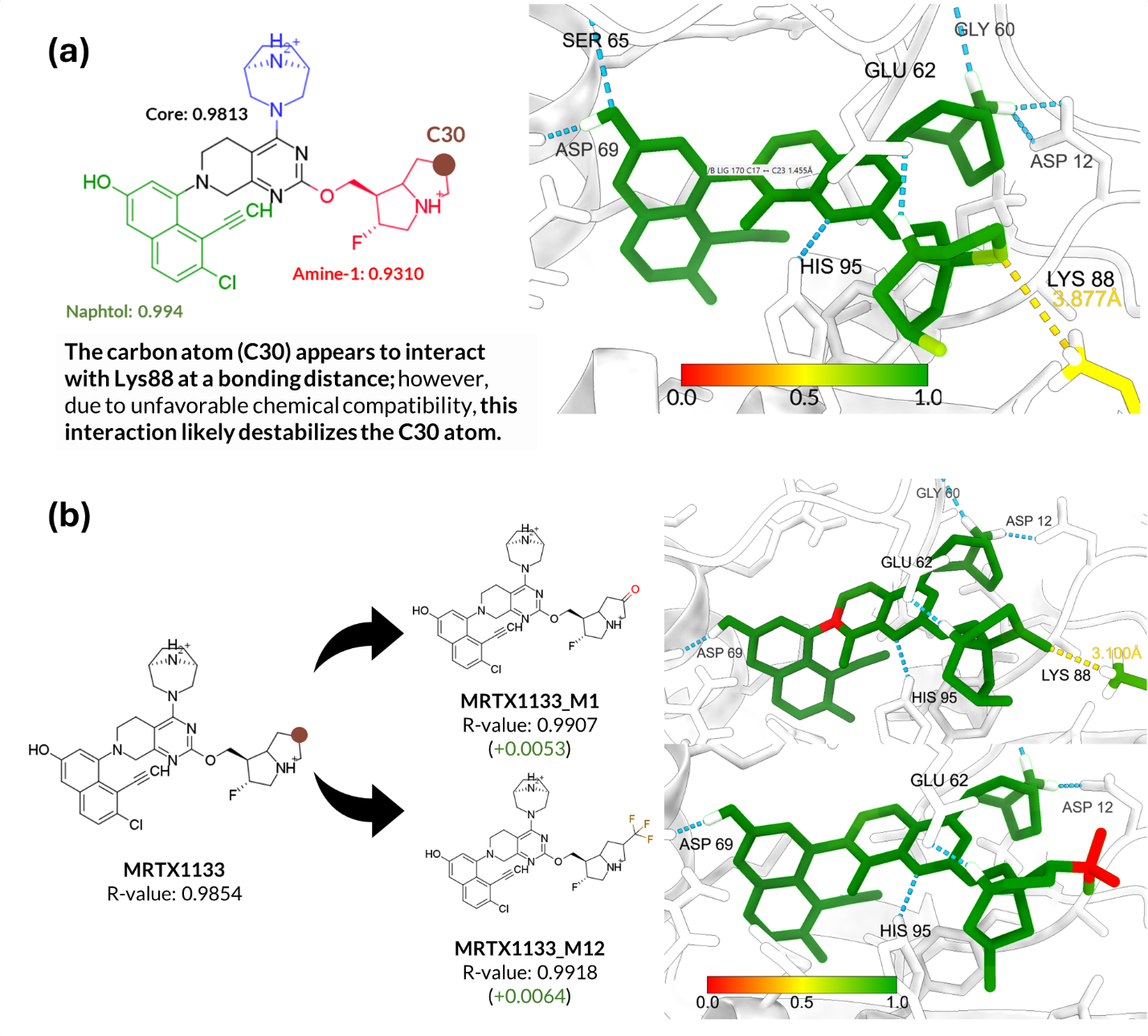


**Fig. S14. Stability profiling and derivative design of MRTX1133.** (a) Scaffold-based R-value profiling of MRTX1133 from HT-MD simulations, showing relative stability of the core, amine groups, naphthol moiety, and C30 atom. (b) Chemical structures and representative binding conformations of MRTX1133 and two derivatives (MRTX1133_M1 and MRTX1133_M12). R-values for each compound are indicated, with color gradients representing contact stability (0–1.0). Key interacting residues are labeled.


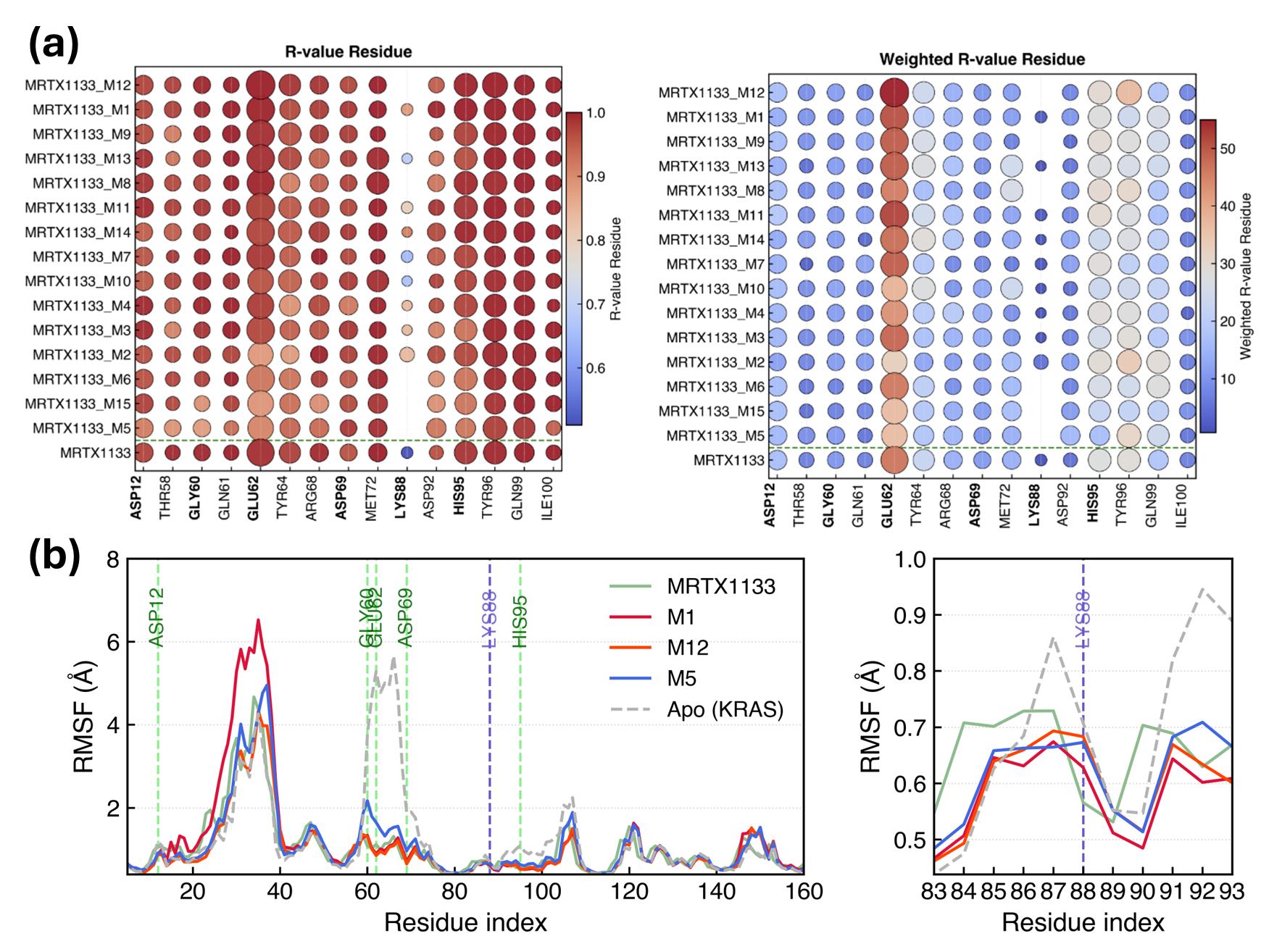


**Fig. S15.** **Residue-level maps and RMSF profiles for MRTX1133 and its analogs (MRTX1133 _Mx).** (a) Residue-level R-value and weighted R-value plots. Ligands are ordered by reference R-value at 400 K (highest at top); the green dashed line separates the parent MRTX1133. Circle area is proportional to contact count, and color encodes the metric in each panel (blue = low, red = high). Key anchoring residues (Asp12, Gly60, Glu62, Asp69, His95) and Lys88 are bolded on the x-axis. (b) RMSF plots for MRTX1133 (parent), M1, M12 (high-stability analogs), and M5 (low-stability analog). The inset showing residue 83-93 to highlight Lys88 fluctuation. The KRAS^G12D^ apo structure is shown as a reference.


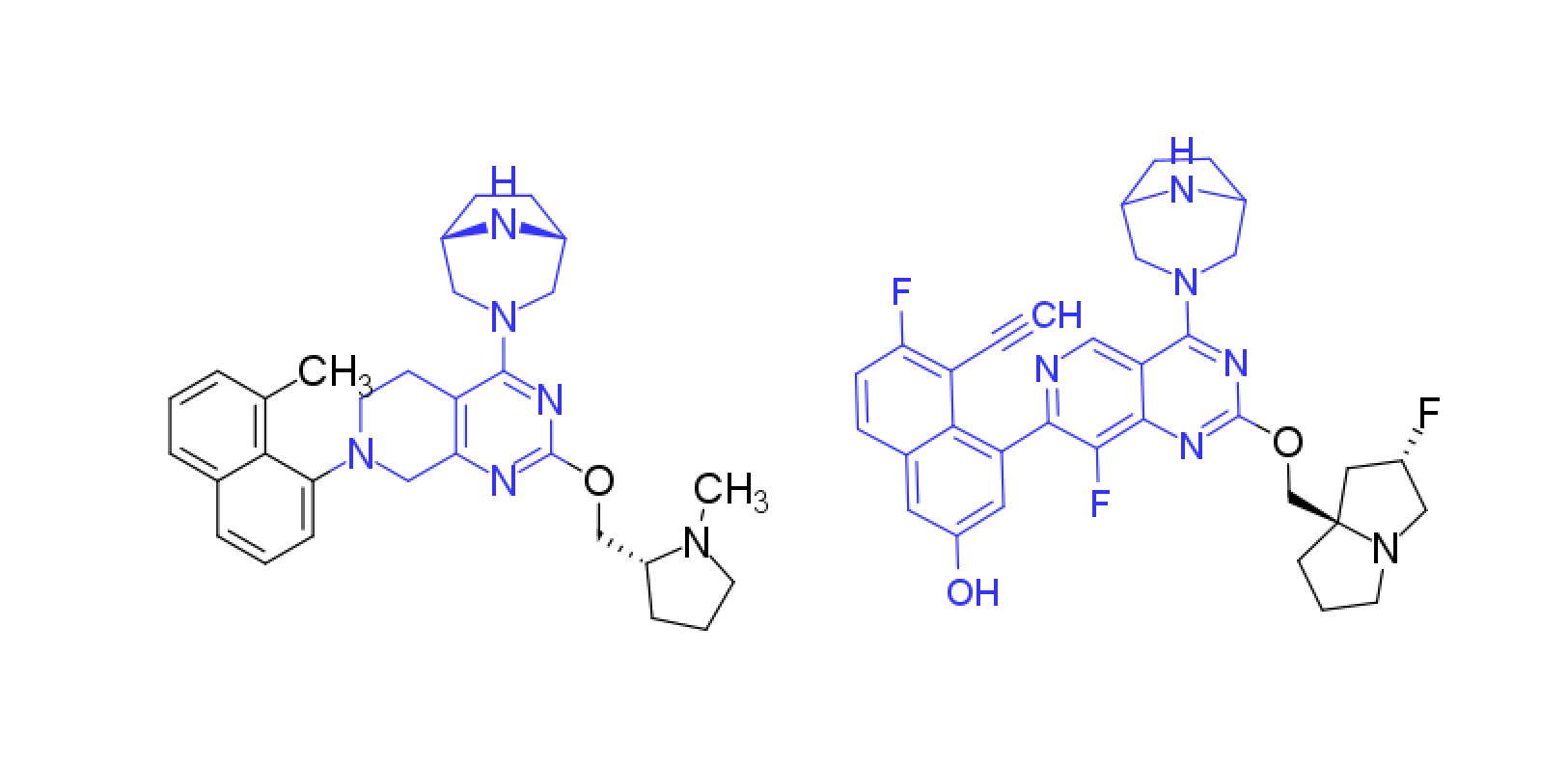


**Fig. S16.** The molecular structure of TH-Z835 (left) and MRTX1133 (right) ligands in their neutral form. The highlighted part shown in blue color depicts the part that selected as the template in template-based docking studies.

**TABLES**

**Table S1**. Full list of the PDB structure used in this study, along with their co-crystallized ligands. All ligands are displayed here in their protonated forms (after protonation at pH 7.0, except for the HIV-1pro system, which was protonated at pH 5.0).

| **No.** | **PDB ID** | **Ligand Contained** | | **Protonation Charge** | **X-Ray Resolution (Å)** | |
| --- | --- | --- | --- | --- | --- | --- |
| **1. Monkeypox VP39 (VP39)** | | | | | | |
| 1 | 8CEQ | TO427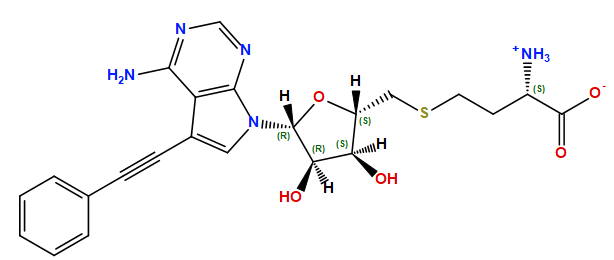 | | 0 | 2.50 | |
| 2 | 8CER | TO494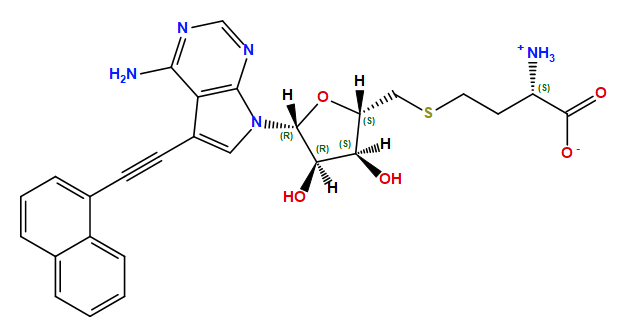 | | 0 | 2.60 | |
| 3 | 8CES | TO500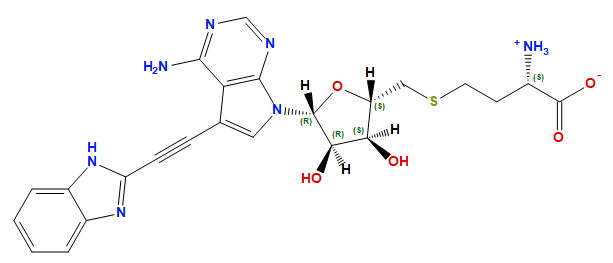 | | 0 | 2.50 | |
| 4 | 8CET | TO507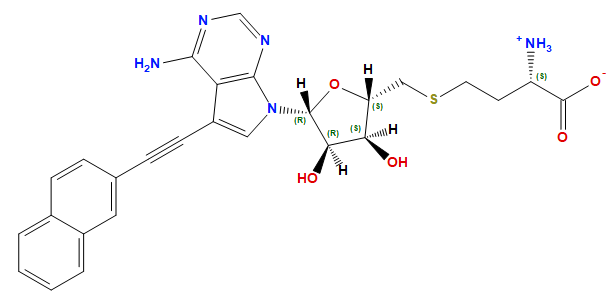 | | 0 | 2.50 | |
| 5 | 8B07 | Sinefungin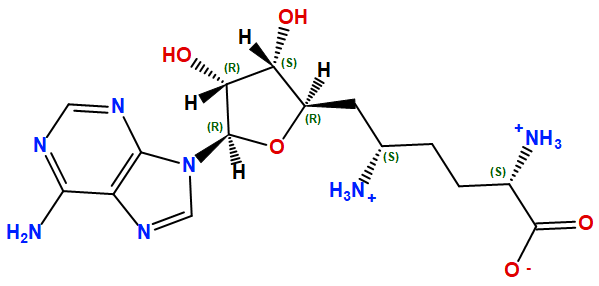 | | +1 | 2.05 | |
| 6 | 8CGB | SAH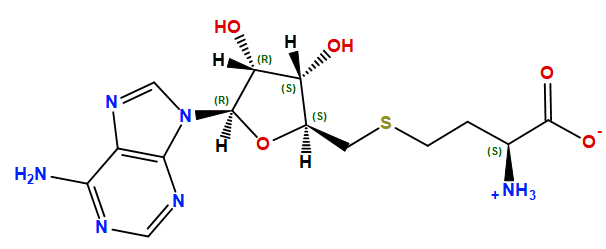 | | 0 | 2.47 | |
| **2. Set domain-containing protein 2 (SETD2)** | | | | | | |
| 1 | 5LSS | Compound 3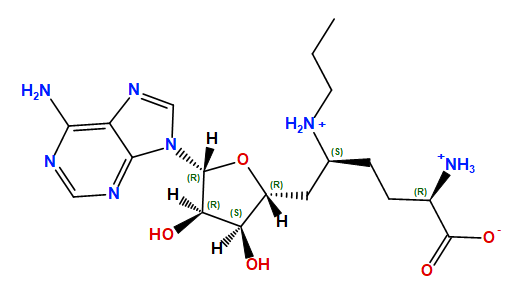 | | +1 | 1.79 | |
| 2 | 5LSY | Compound 4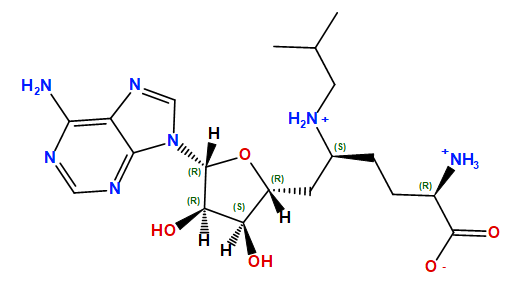 | | +1 | 1.62 | |
| 3 | 5LT7 | Compound 5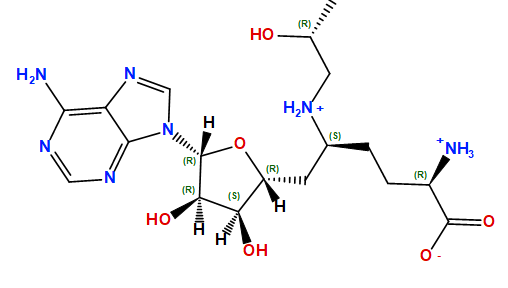 | | +1 | 1.51 | |
| 4 | 5LSZ | Compound 6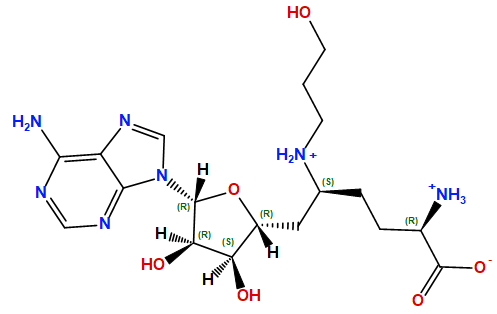 | | +1 | 1.62 | |
| 5 | 5LT6 | Compound 8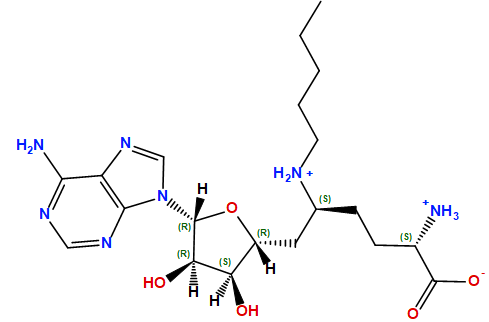 | | +1 | 2.05 | |
| 6 | 5LSX | Compound 9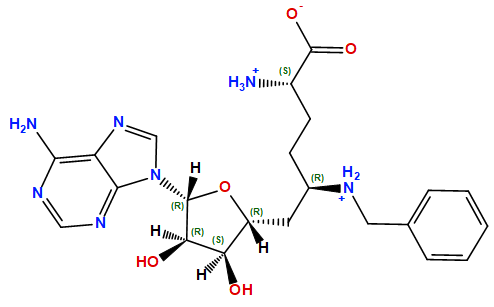 | | +1 | 2.90 | |
| 7 | 5LT8 | Sinefungin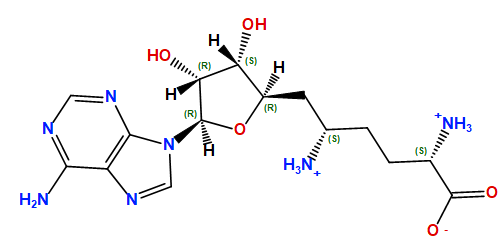 | | +1 | 1.57 | |
| 8 | 5LSU | SAM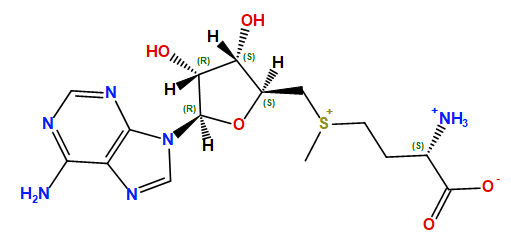 | | +1 | 2.14 | |
| **3. Protein arginine methyltransferases 6 (PRMT6)** | | | | | | |
| 1 | 7NUD | Ligand 12a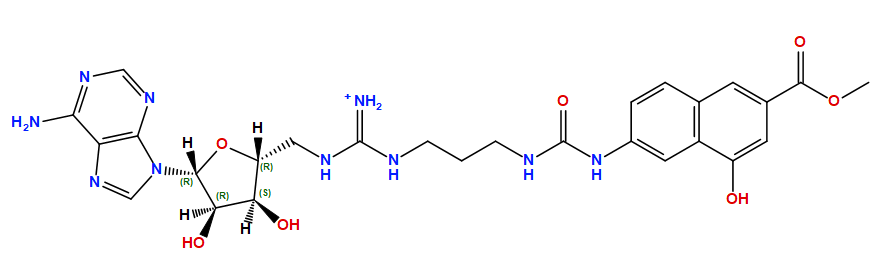 | | +1 | 1.65 | |
| 2 | 7NUE | Ligand 12c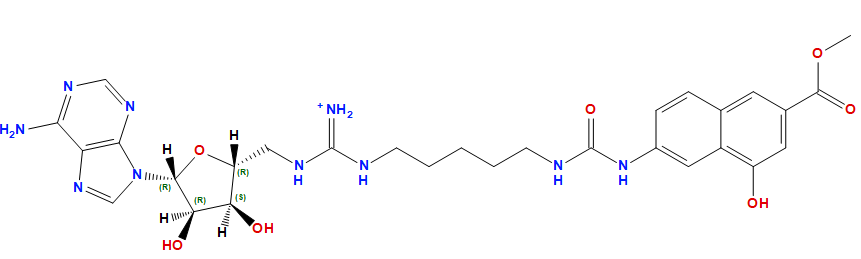 | | +1 | 2.00 | |
| 3 | 7P2R | Ligand 12f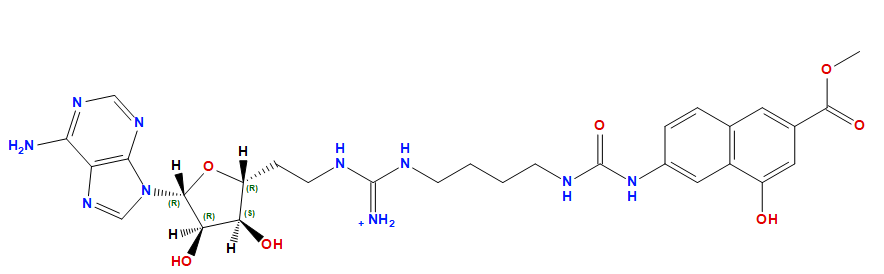 | | +1 | 2.30 | |
| 4 | 5LV4 | LH1236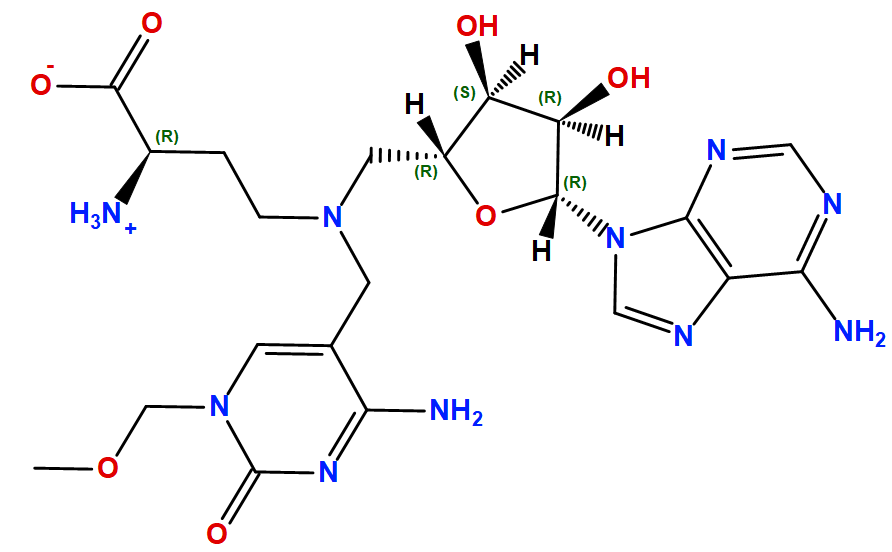 | | 0 | 1.66 | |
| 5 | 5LV5 | LH1458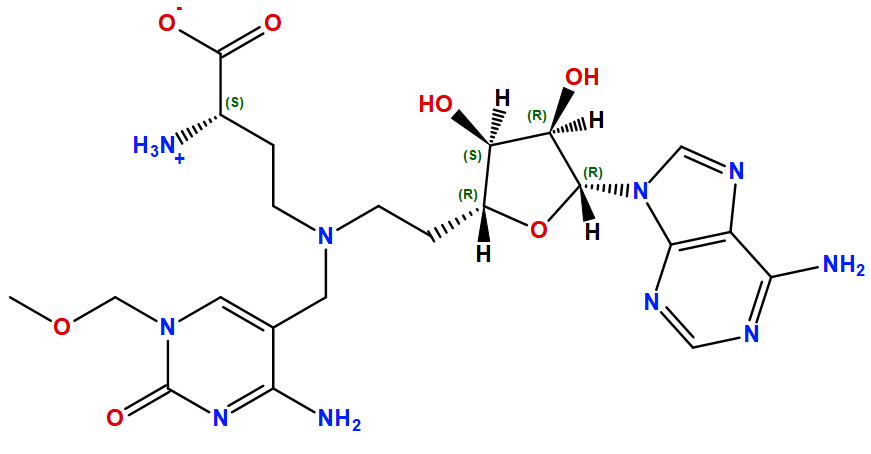 | | 0 | 1.80 | |
| 6 | 6SQ3 | Ligand U1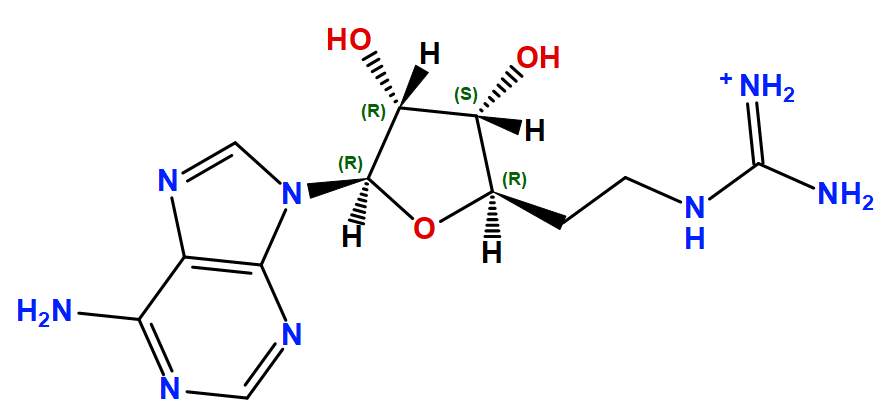 | | +1 | 2.15 | |
| 7 | 6SQ4 | Ligand U2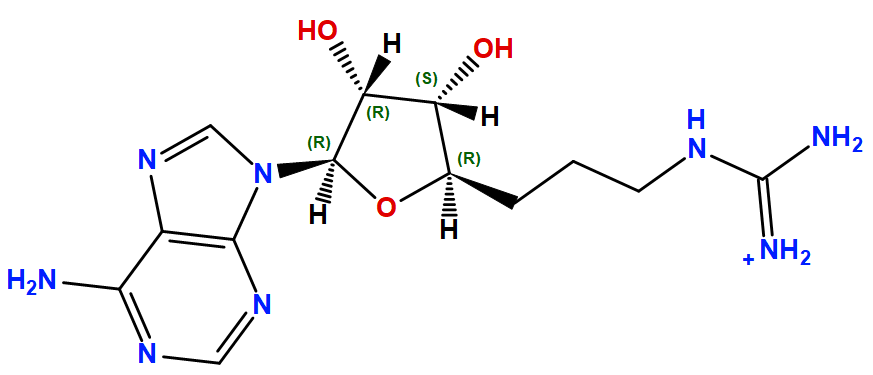 | | +1 | 1.70 | |
| 8 | 4C04 | Sinefungin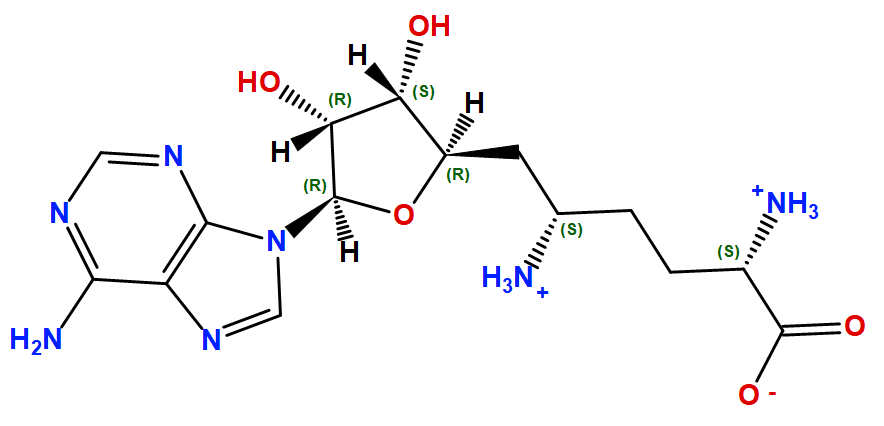 | | +1 | 1.58 | |
| 9 | 4LWP | SAH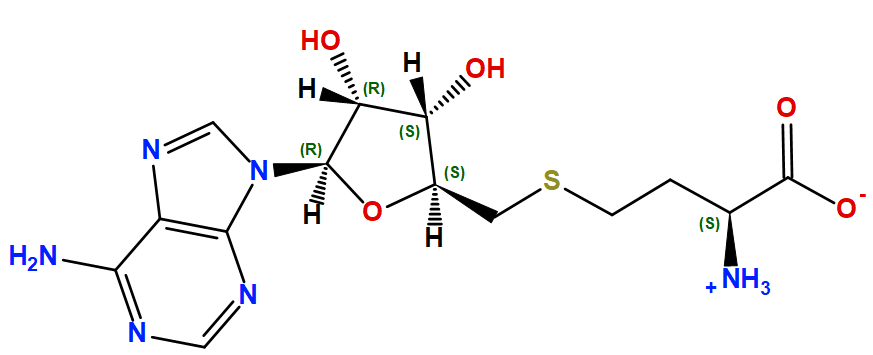 | | 0 | 2.35 | |
| **4.** **HIV-1 protease (HIV-1pro)** | | | | | | |
| 1 | 3OXC | Saquinavir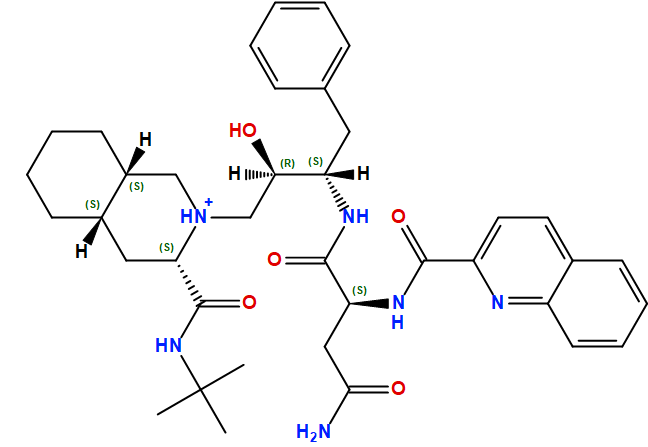 | | +1 | 1.16 | |
| 2 | 2BPX | Indinavir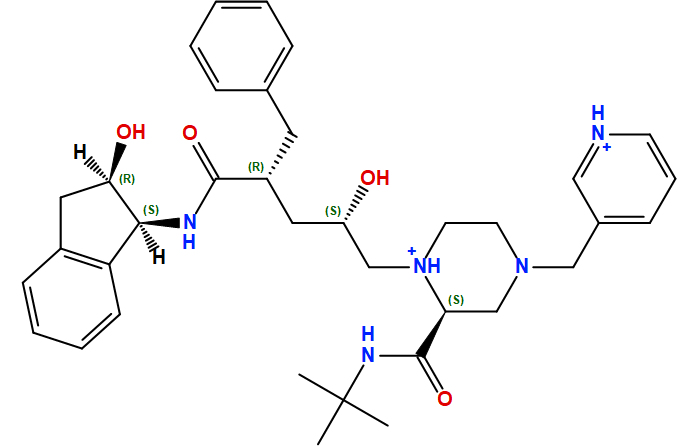 | | +2 | 2.80 | |
| 3 | 2B60 | Ritonavir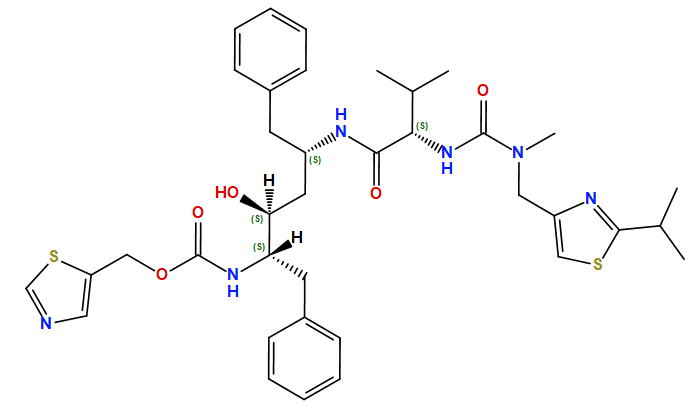 | | 0 | 2.20 | |
| 4 | 3EKX | Nelfinavir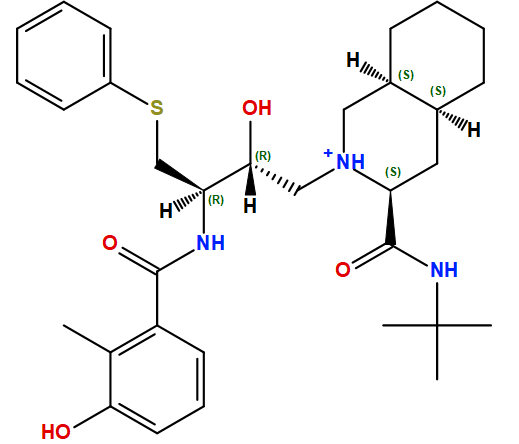 | | +1 | 1.97 | |
| 5 | 3NU3 | Amprenavir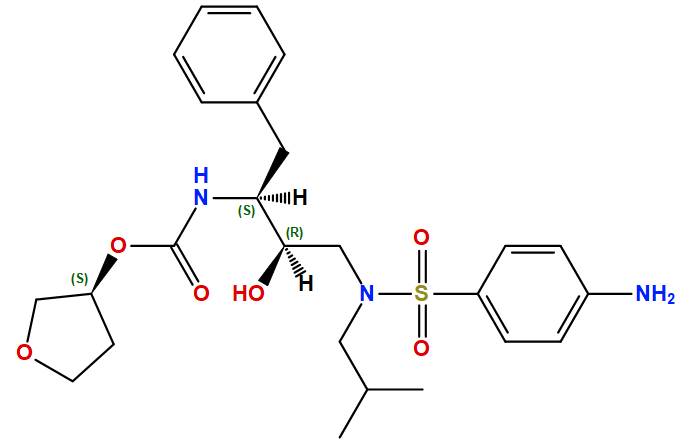 | | 0 | 1.02 | |
| 6 | 1MUI | Lopinavir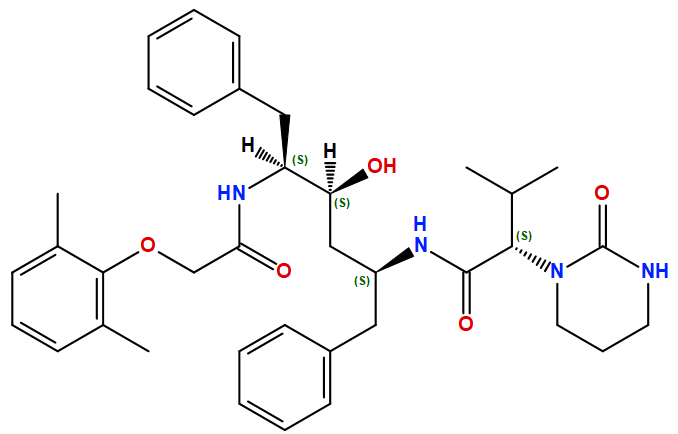 | | 0 | 2.80 | |
| 7 | 2O4K | Atazanavir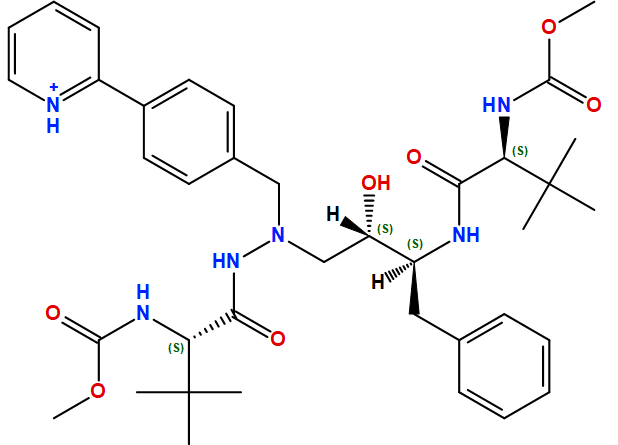 | | +1 | 1.60 | |
| 8 | 6DIF | Tipranavir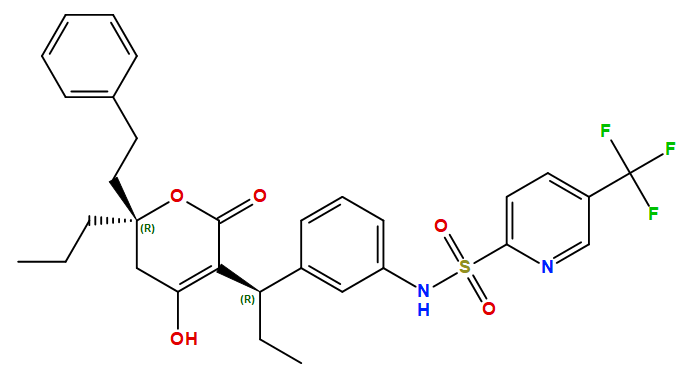 | | 0 | 1.20 | |
| 9 | 4LL3 | Darunavir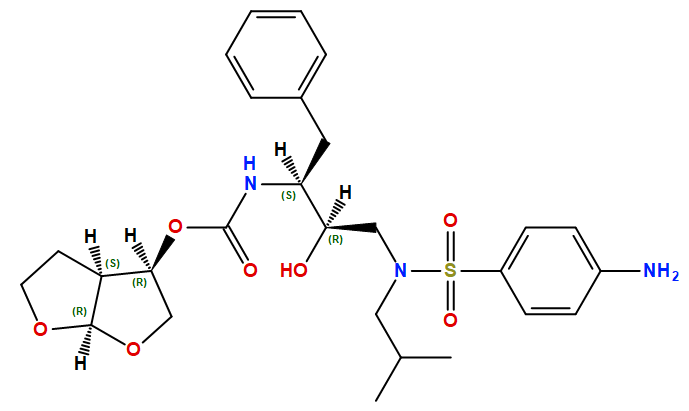 | | 0 | 1.95 | |
| **5.** **SARS-CoV-2 papain-like protease (PLpro)** | | | | | | |
| 1 | 8UOB | Jun12682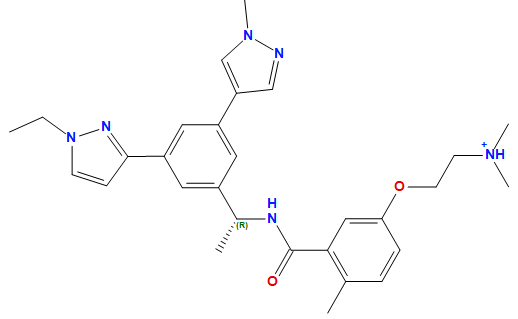 | | +1 | 2.52 | |
| 2 | 8UUF | Jun11941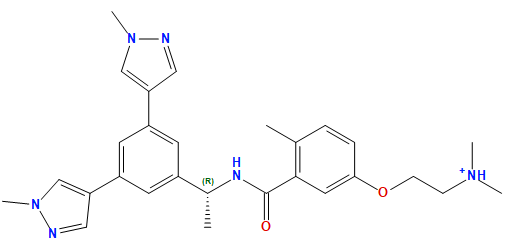 | | +1 | 2.84 | |
| 3 | 8UUG | Jun12303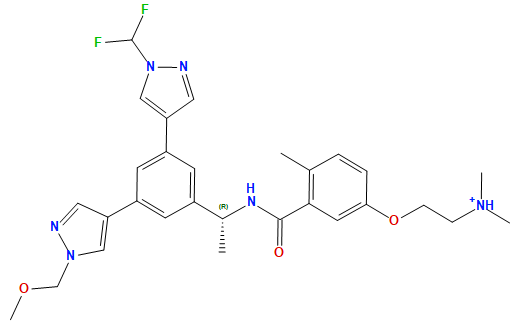 | | +1 | 2.74 | |
| 4 | 8UUH | Jun12199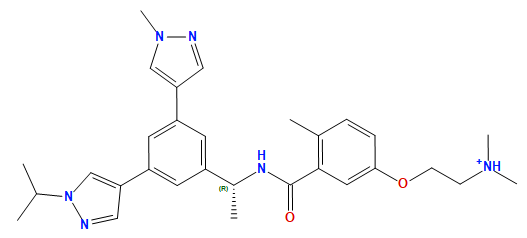 | | +1 | 2.80 | |
| 5 | 8UUU | Jun12162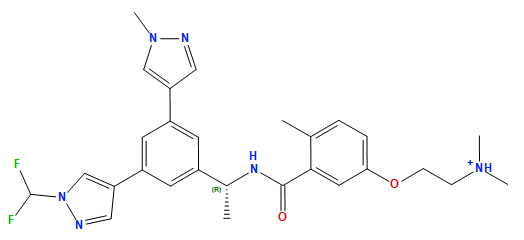 | | +1 | 3.01 | |
| 6 | 8UUV | Jun12197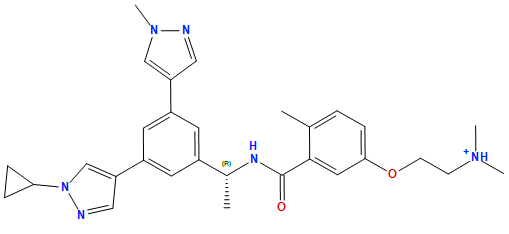 | | +1 | 3.01 | |
| 7 | 8UUY | Jun12129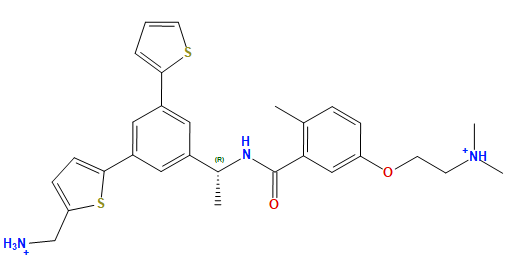 | | +2 | 3.05 | |
| 8 | 9CSY | PF-07957472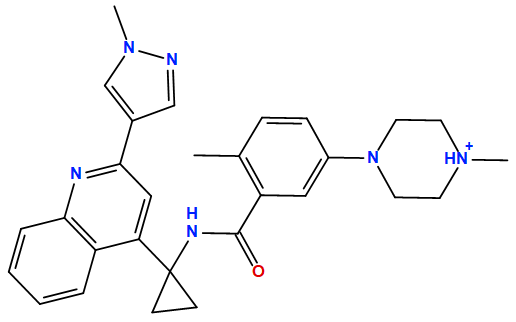 | | +1 | 2.60 | |
| 9 | 9DNU | Jun13296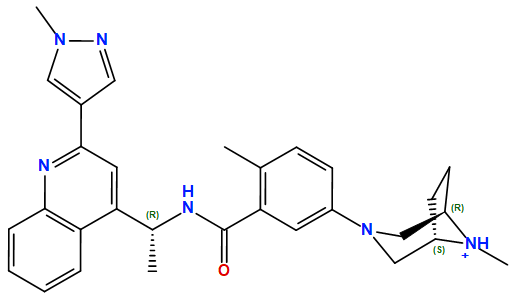 | | +1 | 2.30 | |
| 10 | 9DNV | Jun13308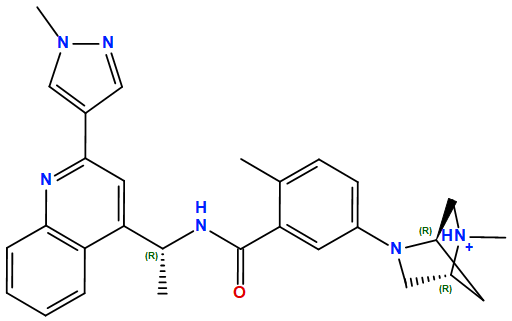 | | +1 | 2.40 | |
| 11 | 9DO1 | Jun13307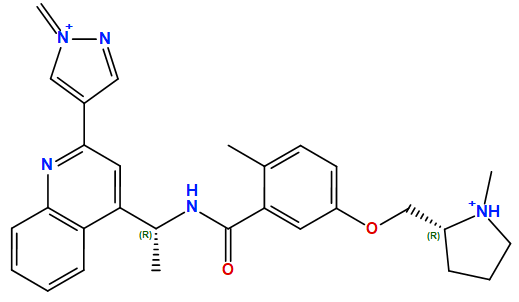 | | +2 | 2.40 | |
| 12 | 9DO3 | Jun13317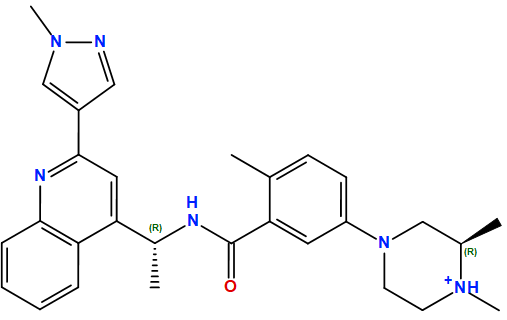 | | +1 | 2.50 | |
| 13 | 9DO5 | Jun12665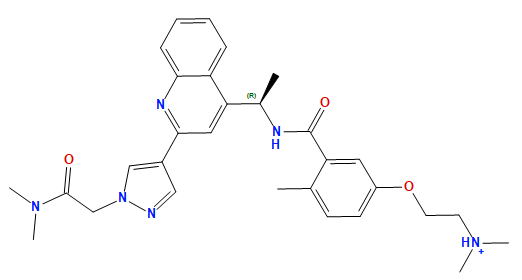 | | +1 | 3.00 | |
| 14 | 9DOI | Jun13306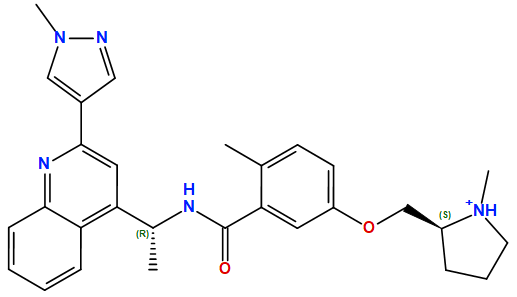 | | +1 | 2.30 | |
| **6.** **ER glucosidase I (ERGluI)** | | | | | | |
| 1 | 7R6J | EB-0668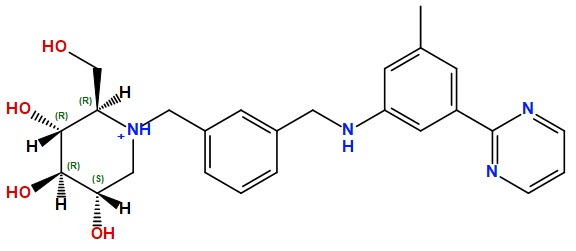 | | +1 | 1.91 | |
| 2 | 7RD2 | EB-0696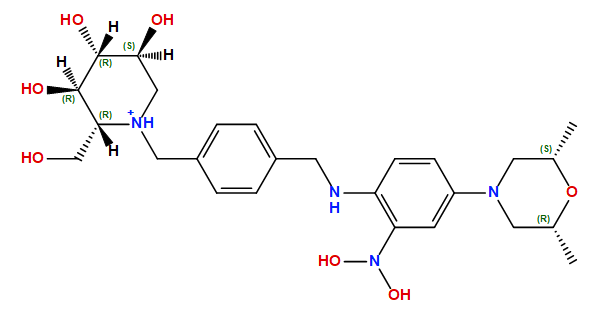 | | +1 | 2.61 | |
| 3 | 7REV | EB-0722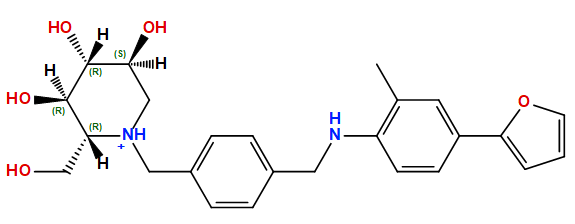 | | +1 | 2.30 | |
| 4 | 8E3J | EB-0128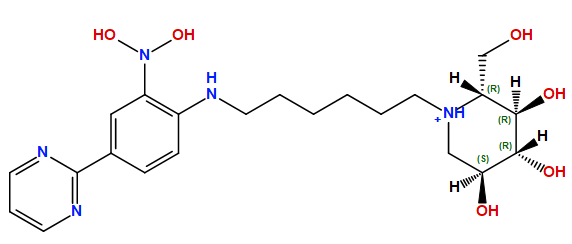 | | +1 | 2.71 | |
| 5 | 8E3P | EB-0334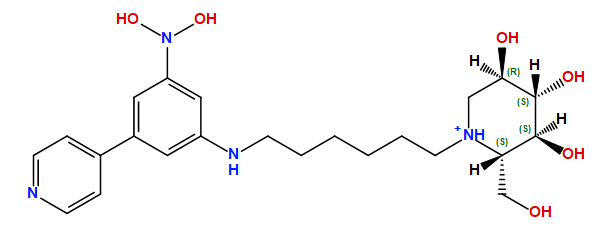 | | +1 | 2.30 | |
| 6 | 8E4I | EB-0442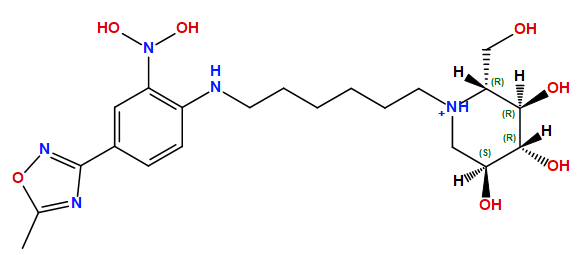 | | +1 | 2.21 | |
| 7 | 8E4K | EB-0484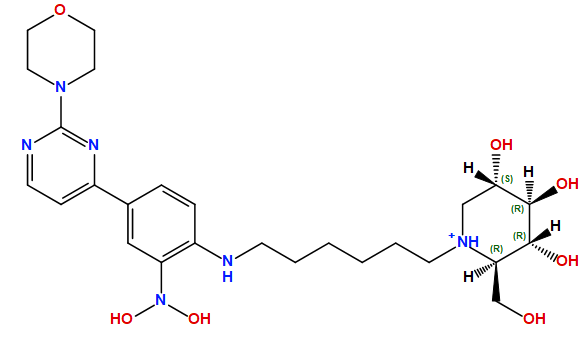 | | +1 | 2.20 | |
| 8 | 8E4Z | EB-0702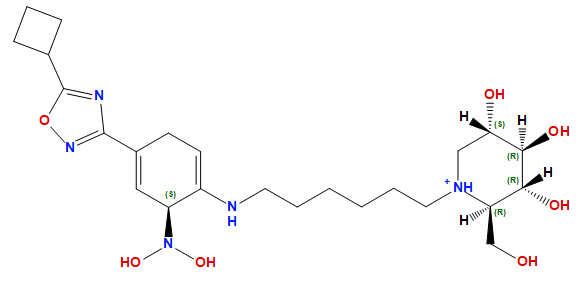 | | +1 | 2.37 | |
| 9 | 8E6G | EB-0702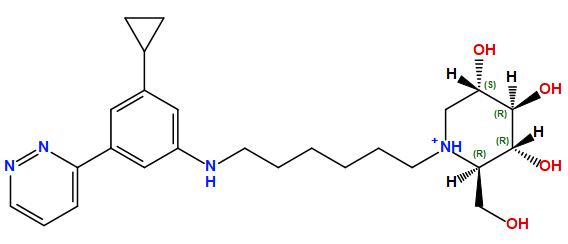 | | +1 | 2.30 | |
| 10 | 8ECW | EB-0485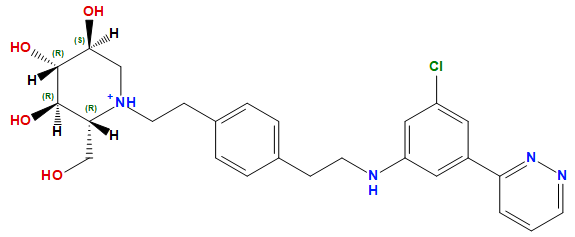 | | +1 | 2.25 | |
| 11 | 8EGV | EB-0543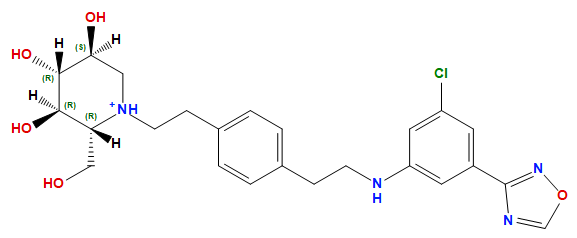 | | +1 | 2.09 | |
| 12 | 8EHP | EB-0584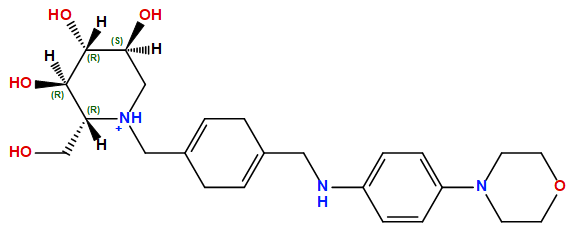 | | +1 | 2.68 | |
| 13 | 8EID | EB-0594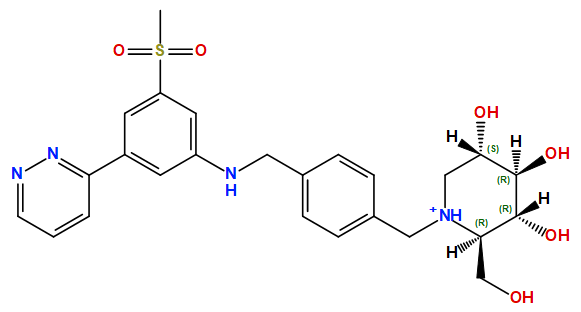 | | +1 | 2.30 | |
| 14 | 8EKN | EB-0651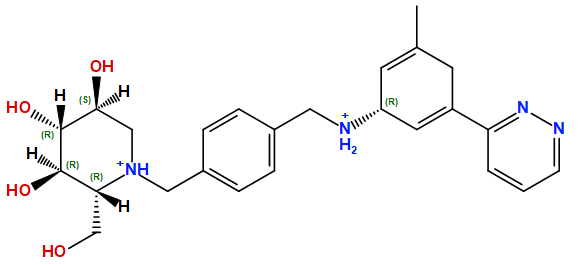 | | +2 | 2.29 | |
| 15 | 8ELE | EB-0692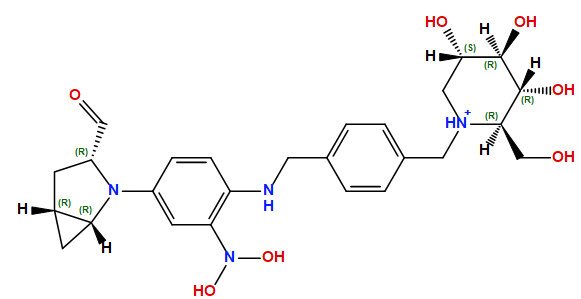 | | +1 | 2.60 | |
| 16 | 8EPJ | EB-0585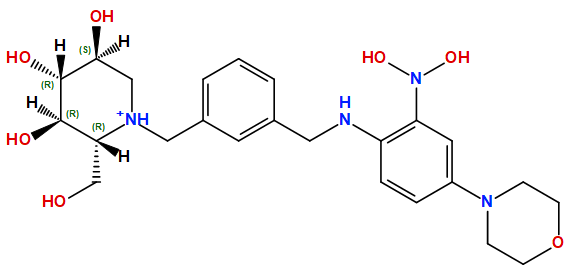 | | +1 | 2.15 | |
| 17 | 8EPO | EB-0659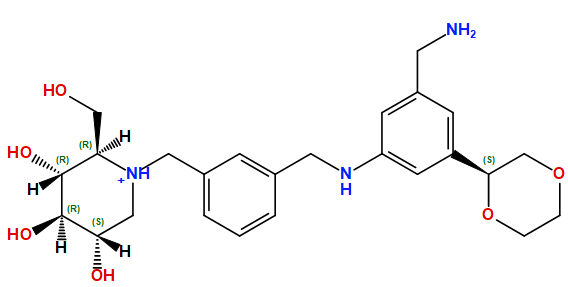 | | +1 | 2.20 | |
| 18 | 8EPR | EB-0663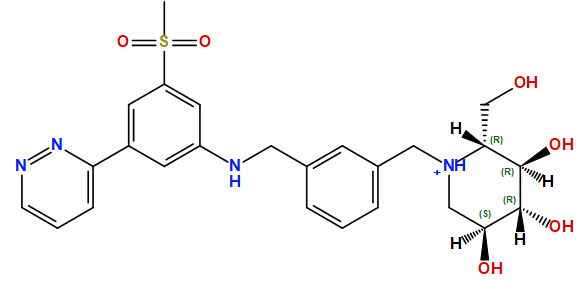 | | +1 | 1.99 | |
| 19 | 8EQ7 | EB-0682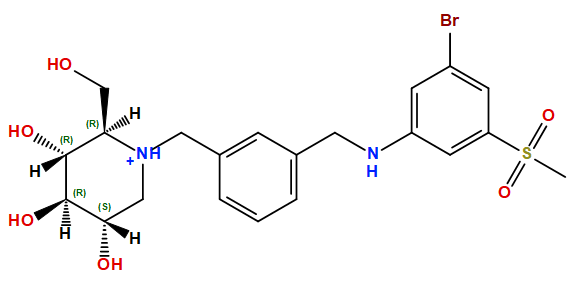 | | +1 | 2.21 | |
| 20 | 8EQX | EB-0732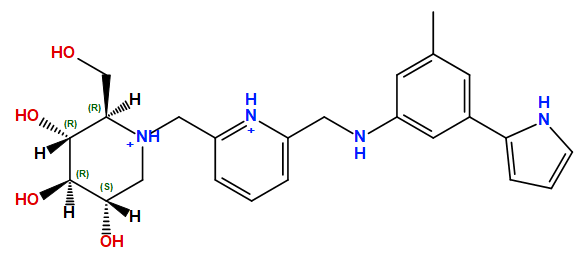 | | +2 | 2.50 | |
| 21 | 8ER4 | EB-0433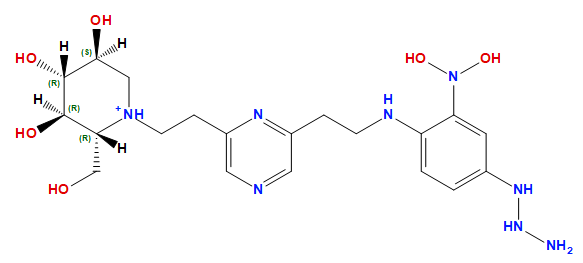 | | +1 | 2.31 | |
| 22 | 8ETL | EB-0149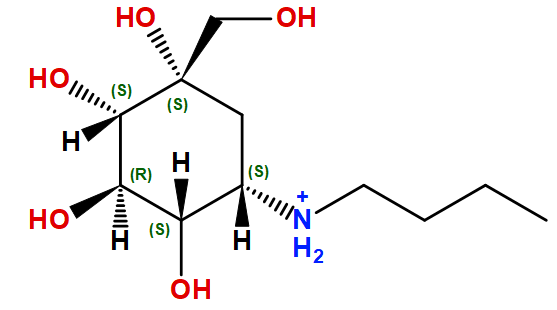 | | +1 | 2.30 | |
| 23 | 8ETO | EB-0156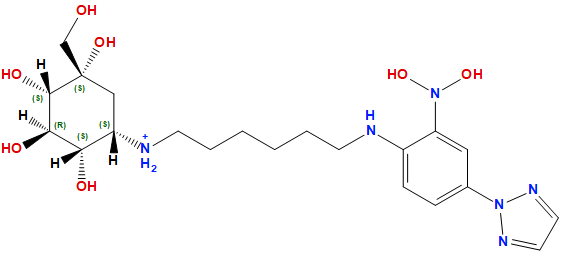 | | +1 | 2.30 | |
| 24 | 8EUD | EB-0356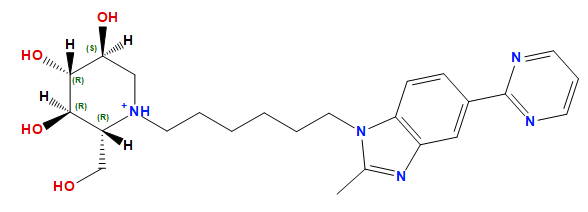 | | +1 | 2.38 | |
| 25 | 8EUR | EB-0288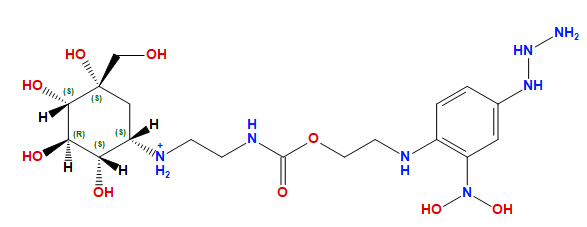 | | +1 | 2.61 | |
| 26 | 8EUT | EB-0030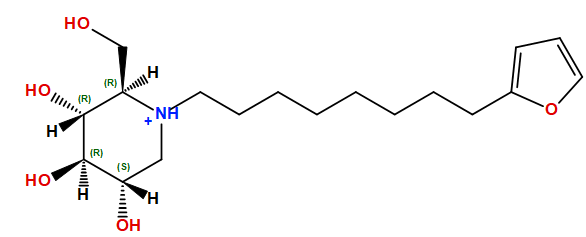 | | +1 | 2.81 | |
| 27 | 8EUX | EB-0037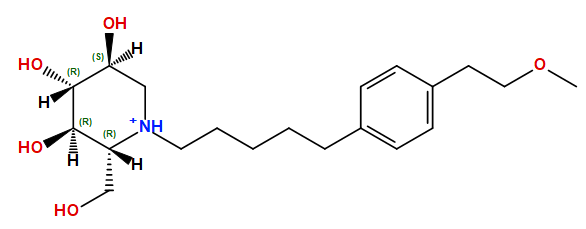 | | +1 | 2.80 | |
| 28 | 7T66 | UV-4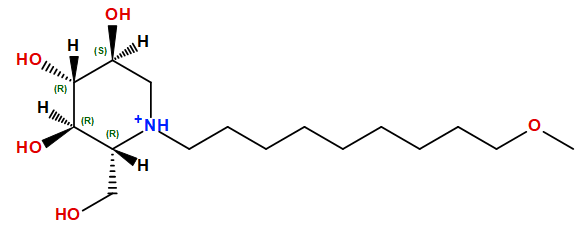 | | +1 | 2.19 | |
| 29 | 7T68 | UV-5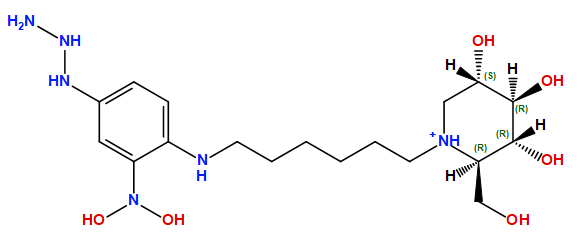 | | +1 | 2.32 | |
| 30 | 7T8V | EB-0159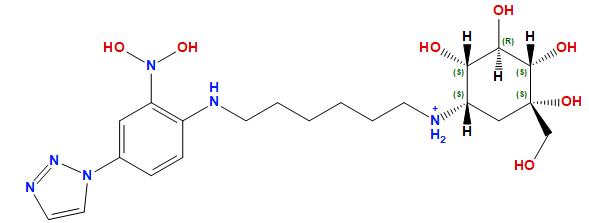 | | +1 | 2.30 | |
| **7.** **Kirsten rat sarcoma virus (KRAS^G12D^)** | | | | | | |
| 1 | 7RPZ | MRTX-1133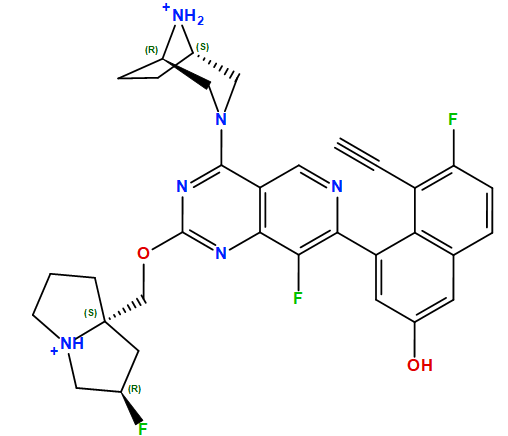 | | +2 | 1.30 | |
| 2 | 7RT1 | Compound 15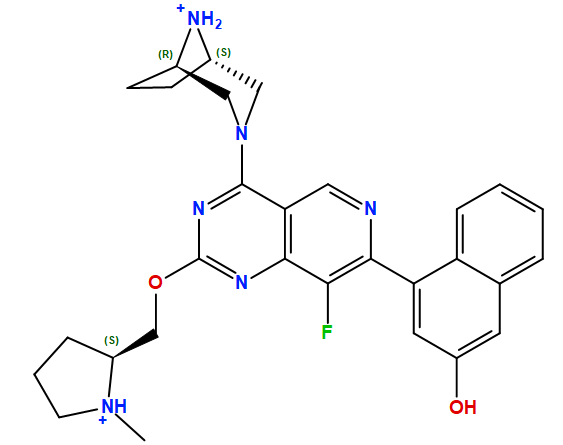 | | +2 | 1.27 | |
| 3 | 7RT2 | Compound 25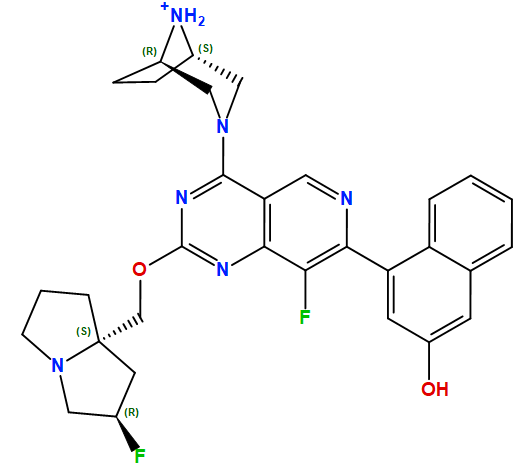 | | +1 | 1.59 | |
| 4 | 7RT3 | Compound 24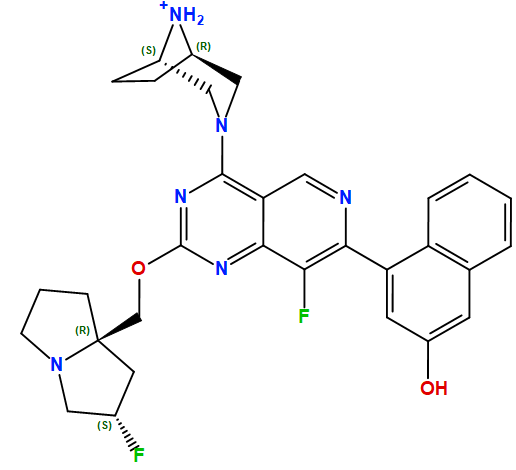 | | +1 | 1.56 | |
| 5 | 7RT4 | Compound 5B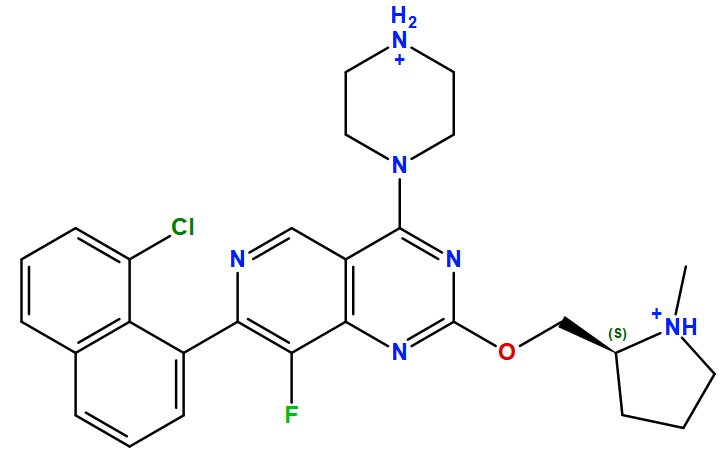 | | +2 | 2.10 | |
| 6 | 7RT5 | Compound 36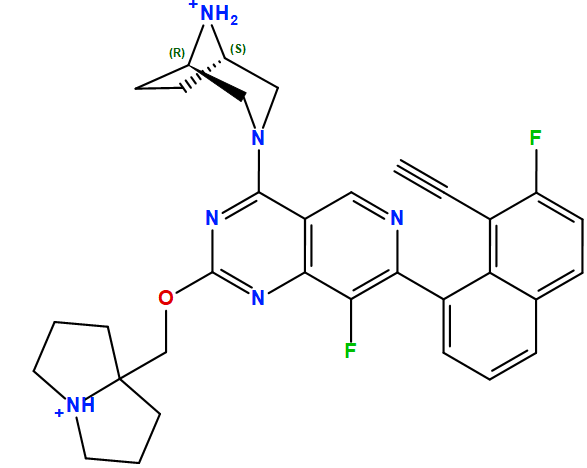 | | +2 | 1.29 | |
| 7 | 7EW9 | TH-Z816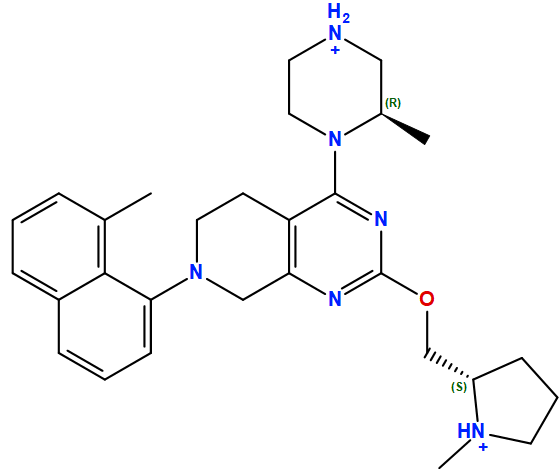 | | +2 | 2.13 | |
| 8 | 7EWA | TH-Z827 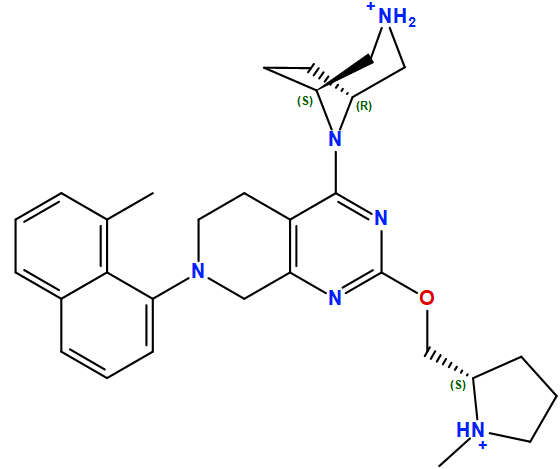 | | +2 | 2.25 | |
| 9 | 7EWB | TH-Z835 | | +2 | 1.99 | |
| 10 | 8TXE | Cp 5 | | +1 | 1.35 | |
| 11 | 8TXG | Cp 8 | | +1 | 1.50 | |
| 12 | 8TXH | Cp 14 | | +1 | 1.20 | |
| **8.** **Mammalian Ste20-like protein kinase 3 (MST3)** | | | | | | |
| 1 | 7B30 | G-5555 | | +1 | 2.10 | |
| 2 | 7B31 | MRIA9 | | +1 | 1.80 | |
| 3 | 7B32 | MRIA7 | | +1 | 1.75 | |
| 4 | 7B33 | MRIA11 | | +1 | 1.90 | |
| 5 | 7B34 | MRIA12 | | +1 | 2.10 | |
| 6 | 7B35 | MRIA13 | | +1 | 2.40 | |
| 7 | 8BZI | MR39 | | +1 | 1.72 | |
| 8 | 8QLQ | JA310 | | +1 | 1.64 | |
| 9 | 8BZJ | MRLW5 | | +1 | 2.52 | |
| 10 | 8QLR | MR24 | | +1 | 1.85 | |
| 11 | 8QLS | MR26 | | +2 | 1.61 | |
| 12 | 8QLT | MR30 | | +1 | 1.47 | |
| **9. Janus Kinase 2 (JAK2) JH1 domain** | | | | | | |
| 1 | 8BM2 | Gandotinib | | +1 | 1.5 | |
| 2 | 8BPV | Pacritinib | | +1 | 1.7 | |
| 3 | 8BPW | Lestaurtinib | 0 | | 1.8 |  |
| 4 | 8BX6 | Cerdaulatinib | | 0 | 1.5 | |
| 5 | 8BX9 | Ilginatinib | | 0 | 1.4 | |
| 6 | 8BXC | Itacitinib | | +1 | 1.9 | |
| 7 | 8BXH | Momelotinib | | 0 | 1.3 | |
| 8 | 6VGL | Ruxolitinib | | 0 | 1.9 | |
| 9 | 6VNE | Fedratinib | | +1 | 1.9 | |
| 10 | 6VN8 | Baricitinib | | 0 | 1.9 | |
| 11 | 6VNB | BL2-084 | | +1 | 2.19 | |
| 12 | 6VNC | BL2-096 | | +1 | 2.3 | |
| 13 | 6VNF | MA9-086 | | +1 | 2.06 | |
| 14 | 6VNG | PN2-118 | | +1 | 2.5 | |
| 15 | 6VNH | PN2-123 | | +1 | 2.4 | |
| 16 | 6VNI | PN3-115 | | +1 | 2.1 | |
| 17 | 6VNJ | PN4-014 | | +1 | 1.9 | |
| 18 | 6VNK | PN4-073 | | 0 | 2 | |
| 19 | 6VNL | SG3-179 | | +1 | 2.4 | |
| 20 | 6VNM | SY5-103 | | +1 | 2.2 | |
| 21 | 6VS3 | BL2-057 | | +1 | 2 | |
| 22 | 6VSN | BL2-110 | | 0 | 2.5 | |
| **10. HMG-CoA reductase (HMGR)** | | | | | | |
| 1 | 1HW8 | Mevastatin | | -1 | 2.10 | |
| 2 | 1HW9 | Simvastatin | | -1 | 2.33 | |
| 3 | 1HWI | Fluvastatin | | -1 | 2.30 | |
| 4 | 1HWJ | Cerivastatin | | -1 | 2.26 | |
| 5 | 1HWK | Atorvastatin | | -1 | 2.22 | |
| 6 | 1HWL | Rosuvastatin | | -1 | 2.10 | |
| 7 | 3CCT | Compound 13 | | -1 | 2.12 | |
| 8 | 3CCW | Compound 1 | | -1 | 2.10 | |
| 9 | 3CCZ | Compound 4 | | -1 | 1.70 | |
| 10 | 3CD0 | Compound 7 | | -1 | 2.40 | |
| 11 | 3CD5 | Compound 9 | | -1 | 2.39 | |
| 12 | 3CD7 | Compound 20 | | -1 | 2.05 | |
| 13 | 3CDA | Compound 15 | | -1 | 2.07 | |
| 14 | 3CDB | Compound 23 | | -2 | 2.30 | |
| 15 | 2Q6B | Compound 22 | | -1 | 2.00 | |

**Table S2**. Full results of HT-MD simulations at 400 K started from PDB structure

| **No.** | **Enzyme Name** | **Ligand Contained** | **R-value** | **RMSD (Å)** | **R(native)-value** | **Ligand Category** |
| --- | --- | --- | --- | --- | --- | --- |
| 1 | Monkeypox VP39 | TO427 | 0.9527 | 1.28 | 0.9193 | SS |
|  |  | TO494 | 0.9233 | 1.107 | 0.8721 | SS |
|  |  | TO500 | 0.8967 | 1.626 | 0.927 | SS |
|  |  | TO507 | 0.9438 | 1.784 | 0.8929 | SS |
|  |  | Sinefungin | 0.8847 | 0.4937 | 0.9278 | SS |
|  |  | SAH | 0.9501 | 0.4729 | 0.9442 | SS |
| 2 | Set domain-containing protein 2 (SETD2) | Compound 3 | 0.7727 | 1.314 | 0.7283 | SS |
|  |  | Compound 4 | 0.8426 | 1.626 | 0.8365 | SS |
|  |  | Compound 5 | 0.9613 | 1.743 | 0.8282 | SS |
|  |  | Compound 6 | 0.7284 | 1.658 | 0.6936 | SS |
|  |  | Compound 8 | 0.8531 | 1.052 | 0.7567 | SS |
|  |  | Compound 9 | 0.6999 | 2.266 | 0.7022 | US |
|  |  | Sinefungin | 0.8104 | 0.7674 | 0.7738 | SS |
|  |  | SAM | 0.7566 | 0.7162 | 0.8741 | SS |
| 3 | ﻿Protein arginine methyltransferase 6 (PRMT6) | Ligand 12a | 0.821 | 0.9812 | 0.7901 | SS |
|  |  | Ligand 12c | 0.7388 | 1.776 | 0.8271 | SS |
|  |  | Ligand 12f | 0.7854 | 1.222 | 0.766 | SS |
|  |  | LH1236 | 0.933 | 1.561 | 0.6583 | SS |
|  |  | LH1458 | 0.7045 | 2.19 | 0.6024 | SS |
|  |  | Ligand U1 | 0.7833 | 0.595 | 0.7837 | SS |
|  |  | Ligand U2 | 0.9207 | 0.7459 | 0.8963 | SS |
|  |  | Sinefungin | 0.9697 | 0.8732 | 0.9448 | SS |
|  |  | SAH | 0.8167 | 0.9749 | 0.793 | SS |
| 4 | HIV-1 protease (HIV-1pro) | Saquinavir | 0.7436 | 1.887 | 0.6686 | SS |
|  |  | Indinavir | 0.758 | 1.29 | 0.7066 | SS |
|  |  | Ritonavir | 0.8889 | 1.823 | 0.7771 | SS |
|  |  | Nelfinavir | 0.8386 | 1.492 | 0.7586 | SS |
|  |  | Amprenavir | 0.9844 | 1.298 | 0.9524 | SS |
|  |  | Lopinavir | 0.8213 | 1.154 | 0.7635 | SS |
|  |  | Atazanavir | 0.9525 | 1.245 | 0.8443 | SS |
|  |  | Tipranavir | 0.9519 | 0.9469 | 0.9231 | SS |
|  |  | Darunavir | 0.8981 | 1.399 | 0.844 | SS |
| 5 | SARS-CoV-2 papain-like protease (PLpro) | Jun12682 | 0.9003 | 1.211 | 0.8525 | SS |
|  |  | Jun11941 | 0.9733 | 0.953 | 0.9494 | SS |
|  |  | Jun12303 | 0.9646 | 1.097 | 0.942 | SS |
|  |  | Jun12199 | 0.9657 | 0.9716 | 0.9398 | SS |
|  |  | Jun12162 | 0.9732 | 1.135 | 0.924 | SS |
|  |  | Jun12197 | 0.9646 | 1.049 | 0.9307 | SS |
|  |  | Jun12129 | 0.9435 | 1.325 | 0.9169 | SS |
|  |  | PF-07957472 | 0.9824 | 0.4029 | 0.9803 | SS |
|  |  | Jun13296 | 0.9816 | 1.311 | 0.9673 | SS |
|  |  | Jun13308 | 0.9862 | 0.7623 | 0.96 | SS |
|  |  | Jun13307 | 0.8853 | 0.9447 | 0.8653 | SS |
|  |  | Jun13317 | 0.9546 | 0.7336 | 0.9484 | SS |
|  |  | Jun12665 | 0.9888 | 1.492 | 0.9413 | SS |
|  |  | Jun13306 | 0.9853 | 0.7102 | 0.9451 | SS |
| 6 | ER glucosidase I (ERGluI) | EB-0668 | 0.8511 | 0.8137 | 0.8149 | SS |
|  |  | EB-0696 | 0.8398 | 2.089 | 0.8425 | SS |
|  |  | EB-0722 | 0.8075 | 2.807 | 0.841 | SS |
|  |  | EB-0128 | 0.8182 | 2.066 | 0.7792 | SS |
|  |  | EB-0334 | 0.5692 | 2.021 | 0.5656 | US |
|  |  | EB-0442 | 0.9539 | 2.529 | 0.7713 | SS |
|  |  | EB-0484 | 0.7418 | 3.656 | 0.7175 | SD |
|  |  | EB-0450 | 0.7175 | 2.598 | 0.7232 | SS |
|  |  | EB-0702 | 0.7314 | 1.33 | 0.7127 | SS |
|  |  | EB-0485 | 0.7612 | 3.321 | 0.7378 | SD |
|  |  | EB-0543 | 0.7503 | 2.809 | 0.7663 | SS |
|  |  | EB-0584 | 0.8981 | 2.572 | 0.8896 | SS |
|  |  | EB-0594 | 0.8101 | 0.9509 | 0.7639 | SS |
|  |  | EB-0651 | 0.91 | 2.465 | 0.9387 | SS |
|  |  | EB-0692 | 0.7638 | 2.828 | 0.8447 | SS |
|  |  | EB-0585 | 0.8178 | 3.259 | 0.8592 | SD |
|  |  | EB-0659 | 0.8544 | 1.83 | 0.8283 | SS |
|  |  | EB-0663 | 0.93 | 2.23 | 0.7882 | SS |
|  |  | EB-0682 | 0.9029 | 2.311 | 0.7131 | SS |
|  |  | EB-0732 | 0.9108 | 3.201 | 0.9321 | SD |
|  |  | EB-0433 | 0.8075 | 1.401 | 0.8346 | SS |
|  |  | EB-0149 | 0.8705 | 2.566 | 0.6687 | SS |
|  |  | EB-0156 | 0.7701 | 0.7085 | 0.8568 | SS |
|  |  | EB-0356 | 0.7561 | 1.706 | 0.7943 | SS |
|  |  | EB-0288 | 0.8847 | 2.103 | 0.8201 | SS |
|  |  | EB-0030 | 0.8785 | 2.401 | 0.9405 | SS |
|  |  | EB-0037 | 0.8504 | 2.41 | 0.8688 | SS |
|  |  | UV-4 | 0.9734 | 1.36 | 0.8829 | SS |
|  |  | UV-5 | 0.8795 | 2.499 | 0.7517 | SS |
|  |  | EB-0159 | 0.7713 | 2.117 | 0.802 | SS |
| 7 | Kirsten rat sarcoma virus (KRAS^G12D^) | MRTX-1133 | 0.9846 | 1.236 | 0.8982 | SS |
|  |  | Compound 15 | 0.96 | 0.4388 | 0.9384 | SS |
|  |  | Compound 25 | 0.8595 | 1.729 | 0.7449 | SS |
|  |  | Compound 24 | 0.8711 | 1.345 | 0.7529 | SS |
|  |  | Compound 5B | 0.7299 | 0.7533 | 0.6573 | SS |
|  |  | Compound 36 | 0.9007 | 0.5151 | 0.7876 | SS |
|  |  | TH-Z816 | 0.7488 | 1.2348 | 0.6948 | SS |
|  |  | TH-Z827 | 0.7184 | 1.404 | 0.6748 | SS |
|  |  | TH-Z835 | 0.7758 | 1.318 | 0.7517 | SS |
|  |  | Cp 5 | 0.8754 | 1.448 | 0.7168 | SS |
|  |  | Cp 8 | 0.8087 | 1.496 | 0.7124 | SS |
|  |  | Cp 14 | 0.8748 | 1.66 | 0.8248 | SS |
| 8 | Mammalian Ste20-like protein kinase 3 (MST3) | G-5555 | 0.9119 | 0.7773 | 0.81 | SS |
|  |  | MRIA9 | 0.9323 | 1.116 | 0.805 | SS |
|  |  | MRIA7 | 0.8508 | 0.8355 | 0.8191 | SS |
|  |  | MRIA11 | 0.9209 | 1.246 | 0.7638 | SS |
|  |  | MRIA12 | 0.9003 | 1.37 | 0.7829 | SS |
|  |  | MRIA13 | 0.9469 | 1.046 | 0.8419 | SS |
|  |  | MR39 | 0.9308 | 1.358 | 0.7923 | SS |
|  |  | JA310 | 0.7131 | 0.6837 | 0.7221 | SS |
|  |  | MRLW5 | 0.948 | 1.225 | 0.8143 | SS |
|  |  | MR24 | 0.9503 | 1.488 | 0.8614 | SS |
|  |  | MR26 | 0.9287 | 1.268 | 0.828 | SS |
|  |  | MR30 | 0.8367 | 1.453 | 0.8446 | SS |
| 9 | Janus Kinase 2 (JAK2) JH1 domain | Gandotinib | 0.8406 | 0.9326 | 0.8523 | SS |
|  |  | Pacritinib | 0.9732 | 0.9447 | 0.892 | SS |
|  |  | Lestaurtinib | 0.9986 | 0.2252 | 0.9919 | SS |
|  |  | Cerdaulatinib | 0.9901 | 1.588 | 0.9744 | SS |
|  |  | Ilginatinib | 0.9976 | 0.4822 | 0.9962 | SS |
|  |  | Itacitinib | 0.9227 | 1.28 | 0.8617 | SS |
|  |  | Momelotinib | 0.9835 | 0.4747 | 0.9681 | SS |
|  |  | Ruxolitinib | 0.9486 | 0.8695 | 0.9075 | SS |
|  |  | Fedratinib | 0.9624 | 1.498 | 0.9032 | SS |
|  |  | Baricitinib | 0.9814 | 0.7964 | 0.9483 | SS |
|  |  | BL2-084 | 0.9624 | 0.5485 | 0.9843 | SS |
|  |  | BL2-096 | 0.9955 | 1.022 | 0.9768 | SS |
|  |  | MA9-086 | 0.9393 | 1.477 | 0.8934 | SS |
|  |  | PN2-118 | 0.9648 | 1.5 | 0.9397 | SS |
|  |  | PN2-123 | 0.9669 | 1.104 | 0.9398 | SS |
|  |  | PN3-115 | 0.9762 | 1.182 | 0.9558 | SS |
|  |  | PN4-014 | 0.9904 | 1.239 | 0.9439 | SS |
|  |  | PN4-073 | 0.9831 | 1.197 | 0.9395 | SS |
|  |  | SG3-179 | 0.8028 | 2.014 | 0.8972 | SS |
|  |  | SY5-103 | 0.9786 | 0.7314 | 0.9626 | SS |
|  |  | BL2-057 | 0.9811 | 1.625 | 0.946 | SS |
|  |  | BL2-110 | 0.9575 | 0.3169 | 0.9231 | SS |
| 10 | HMG-CoA reductase (HMGR) | Mevastatin | 0.7343 | 1.007 | 0.7714 | SS |
|  |  | Simvastatin | 0.8847 | 0.77 | 0.8195 | SS |
|  |  | Fluvastatin | 0.8762 | 0.5661 | 0.7852 | SS |
|  |  | Cerivastatin | 0.779 | 0.6224 | 0.8024 | SS |
|  |  | Atorvastatin | 0.746 | 0.6391 | 0.6517 | SS |
|  |  | Rosuvastatin | 0.651 | 1.925 | 0.428 | US |
|  |  | Compound 13 | 0.8059 | 1.295 | 0.8405 | SS |
|  |  | Compound 1 | 0.8766 | 2.407 | 0.6418 | SS |
|  |  | Compound 4 | 0.7478 | 1.547 | 0.723 | SS |
|  |  | Compound 7 | 0.6483 | 1.809 | 0.559 | US |
|  |  | Compound 9 | 0.8879 | 1.383 | 0.8438 | SS |
|  |  | Compound 20 | 0.9585 | 0.9904 | 0.8437 | SS |
|  |  | Compound 15 | 0.9301 | 1.243 | 0.7918 | SS |
|  |  | Compound 23 | 0.8891 | 1.357 | 0.8431 | SS |
|  |  | Compound 22 | 0.877 | 0.8308 | 0.8612 | SS |

**Table S3**. Full HT-MD simulations results at 400 K for representative docking poses, showing the highest R-value among all poses for each ligand.

| **No.** | **Enzyme Name** | **Ligand Contained** | **R-value** | **RMSD Post-MDS (Å)** | **R(dock)-value** | **RMSD Post-Dock (Å)** | **Ligand Category** |
| --- | --- | --- | --- | --- | --- | --- | --- |
| 1 | Monkeypox VP39 | TO427 | 0.9675 | 1.121 | 0.9331 | 0.7883 | SS |
|  |  | TO494 | 0.9505 | 0.8545 | 0.9038 | 1.393 | SS |
|  |  | TO500 | 0.9563 | 1.377 | 0.9286 | 1.259 | SS |
|  |  | TO507 | 0.8531 | 1.855 | 0.7926 | 2.47 | SS |
|  |  | Sinefungin | 0.9694 | 1.696 | 0.8493 | 2.048 | SS |
|  |  | SAH | 0.9725 | 0.7208 | 0.8193 | 2.03 | SS |
| 2 | Set domain-containing protein 2 (SETD2) | Compound 3 | 0.8966 | 1.715 | 0.7754 | 0.735 | SS |
|  |  | Compound 4 | 0.897 | 2.047 | 0.7274 | 1.735 | SS |
|  |  | Compound 5 | 0.7438 | 1.864 | 0.7722 | 0.9724 | SS |
|  |  | Compound 6 | 0.8027 | 2.316 | 0.7616 | 2.392 | SS |
|  |  | Compound 8 | 0.7788 | 1.025 | 0.7992 | 0.6803 | SS |
|  |  | Compound 9 | 0.84 | 2.559 | 0.777 | 1.764 | SS |
|  |  | Sinefungin | 0.8939 | 1.141 | 0.8351 | 0.8219 | SS |
|  |  | SAM | 0.8535 | 0.6959 | 0.7966 | 0.7494 | SS |
| 3 | ﻿Protein arginine methyltransferase 6 (PRMT6) | Ligand 12a | 0.8316 | 4.73 | 0.8841 | 4.009 | SD |
|  |  | Ligand 12c | 0.7514 | 3.622 | 0.7083 | 4.022 | SD |
|  |  | Ligand 12f | 0.6314 | 3.507 | 0.6365 | 3.339 | UD |
|  |  | LH1236 | 0.9139 | 2.035 | 0.8713 | 2.223 | SS |
|  |  | LH1458 | 0.8733 | 3.339 | 0.7284 | 2.609 | SD |
|  |  | Ligand U1 | 0.9665 | 0.7217 | 0.8625 | 0.8063 | SS |
|  |  | Ligand U2 | 0.9052 | 1.147 | 0.7867 | 1.879 | SS |
|  |  | Sinefungin | 0.9266 | 1.884 | 0.8643 | 1.913 | SS |
|  |  | SAH | 0.8693 | 1.258 | 0.8469 | 1.457 | SS |
| 4 | HIV-1 protease (HIV-1pro) | Saquinavir | 0.8782 | 3.802 | 0.6697 | 3.683 | SD |
|  |  | Indinavir | 0.9248 | 3.734 | 0.8007 | 4.223 | SD |
|  |  | Ritonavir | 0.8626 | 1.305 | 0.7464 | 2.241 | SS |
|  |  | Nelfinavir | 0.8642 | 3.925 | 0.7741 | 3.822 | SD |
|  |  | Amprenavir | 0.9743 | 1.2 | 0.9528 | 0.7374 | SS |
|  |  | Lopinavir | 0.9738 | 1.702 | 0.9077 | 1.929 | SS |
|  |  | Atazanavir | 0.9253 | 1.375 | 0.748 | 2.263 | SS |
|  |  | Tipranavir | 0.9097 | 1.988 | 0.7215 | 1.037 | SS |
|  |  | Darunavir | 0.9659 | 1.203 | 0.8556 | 1.401 | SS |
| 5 | SARS-CoV-2 papain-like protease (PLpro) | Jun12682 | 0.9734 | 1.022 | 0.9462 | 1.177 | SS |
|  |  | Jun11941 | 0.9907 | 1.332 | 0.8359 | 1.883 | SS |
|  |  | Jun12303 | 0.9844 | 1.614 | 0.9419 | 1.472 | SS |
|  |  | Jun12199 | 0.9871 | 4.961 | 0.8241 | 4.186 | SD |
|  |  | Jun12162 | 0.979 | 4.82 | 0.9521 | 4.646 | SD |
|  |  | Jun12197 | 0.9772 | 1.289 | 0.9208 | 0.9726 | SS |
|  |  | Jun12129 | 0.9721 | 4.699 | 0.9442 | 4.432 | SD |
|  |  | PF-07957472 | 0.9873 | 0.3933 | 0.9869 | 0.9239 | SS |
|  |  | Jun13296 | 0.9859 | 3.653 | 0.9651 | 3.649 | SD |
|  |  | Jun13308 | 0.9913 | 3.655 | 0.9279 | 3.791 | SD |
|  |  | Jun13307 | 0.9571 | 3.857 | 0.736 | 2.714 | SD |
|  |  | Jun13317 | 0.9906 | 3.704 | 0.9686 | 3.814 | SD |
|  |  | Jun12665 | 0.9866 | 1.81 | 0.9351 | 1.94 | SS |
|  |  | Jun13306 | 0.9853 | 3.776 | 0.9354 | 3.757 | SD |
| 6 | ER glucosidase I (ERGluI) | EB-0668 | 0.9192 | 2.083 | 0.6471 | 1.848 | SS |
|  |  | EB-0696 | 0.8918 | 1.929 | 0.7311 | 1.988 | SS |
|  |  | EB-0722 | 0.9097 | 1.646 | 0.8151 | 1.635 | SS |
|  |  | EB-0128 | 0.7961 | 3.068 | 0.7257 | 1.883 | SD |
|  |  | EB-0334 | 0.8632 | 2.533 | 0.5661 | 3.123 | SS |
|  |  | EB-0442 | 0.928 | 2.511 | 0.6015 | 2.478 | SS |
|  |  | EB-0484 | 0.8511 | 2.721 | 0.7975 | 3.535 | SS |
|  |  | EB-0450 | 0.9138 | 2.085 | 0.6611 | 1.543 | SS |
|  |  | EB-0702 | 0.864 | 2.17 | 0.5808 | 2.062 | SS |
|  |  | EB-0485 | 0.8593 | 2.019 | 0.8139 | 1.676 | SS |
|  |  | EB-0543 | 0.833 | 3.067 | 0.736 | 2.852 | SD |
|  |  | EB-0584 | 0.8882 | 1.484 | 0.8106 | 1.731 | SS |
|  |  | EB-0594 | 0.8792 | 2.641 | 0.7286 | 3.065 | SS |
|  |  | EB-0651 | 0.9465 | 2.37 | 0.7842 | 2.407 | SS |
|  |  | EB-0692 | 0.9042 | 2.084 | 0.8338 | 2.418 | SS |
|  |  | EB-0585 | 0.7302 | 2.504 | 0.7341 | 2.048 | SS |
|  |  | EB-0659 | 0.9134 | 2.643 | 0.7895 | 1.336 | SS |
|  |  | EB-0663 | 0.9324 | 2.667 | 0.7094 | 3.61 | SS |
|  |  | EB-0682 | 0.9655 | 1.909 | 0.697 | 2.917 | SS |
|  |  | EB-0732 | 0.9363 | 1.972 | 0.8007 | 2.856 | SS |
|  |  | EB-0433 | 0.964 | 1.387 | 0.763 | 1.293 | SS |
|  |  | EB-0149 | 0.9084 | 3.101 | 0.7261 | 2.879 | SD |
|  |  | EB-0156 | 0.9733 | 0.5648 | 0.8902 | 0.6321 | SS |
|  |  | EB-0356 | 0.9155 | 2.764 | 0.75 | 2.974 | SS |
|  |  | EB-0288 | 0.8339 | 3.322 | 0.7355 | 3.462 | SD |
|  |  | EB-0030 | 0.9415 | 1.731 | 0.6088 | 3.921 | SS |
|  |  | EB-0037 | 0.9679 | 1.516 | 0.7471 | 1.111 | SS |
|  |  | UV-4 | 0.9815 | 1.226 | 0.7781 | 1.913 | SS |
|  |  | UV-5 | 0.9303 | 1.11 | 0.687 | 3.267 | SS |
|  |  | EB-0159 | 0.6385 | 4.4 | 0.6802 | 3.181 | UD |
| 7 | Kirsten rat sarcoma virus (KRAS^G12D^) | MRTX-1133 | 0.9263 | 1.364 | 0.8296 | 1.116 | SS |
|  |  | Compound 15 | 0.86 | 1.358 | 0.5896 | 1.367 | SS |
|  |  | Compound 25 | 0.846 | 1.466 | 0.7387 | 1.794 | SS |
|  |  | Compound 24 | 0.8289 | 2.368 | 0.7187 | 2.044 | SS |
|  |  | Compound 5B | 0.8932 | 0.4073 | 0.8433 | 1.397 | SS |
|  |  | Compound 36 | 0.8364 | 1.026 | 0.7512 | 1.298 | SS |
|  |  | TH-Z816 | 0.9188 | 1.304 | 0.8473 | 1.114 | SS |
|  |  | TH-Z827 | 0.8864 | 0.7987 | 0.8069 | 1.174 | SS |
|  |  | TH-Z835 | 0.8485 | 1.746 | 0.7111 | 1.554 | SS |
|  |  | Cp 5 | 0.8884 | 1.696 | 0.7663 | 1.09 | SS |
|  |  | Cp 8 | 0.9348 | 1.329 | 0.8761 | 1.33 | SS |
|  |  | Cp 14 | 0.9688 | 0.9981 | 0.7644 | 0.9135 | SS |
| 8 | Mammalian Ste20-like protein kinase 3 (MST3) | G-5555 | 0.9694 | 1.394 | 0.7357 | 1.398 | SS |
|  |  | MRIA9 | 0.9478 | 1.603 | 0.8591 | 1.478 | SS |
|  |  | MRIA7 | 0.9253 | 1.667 | 0.8299 | 1.616 | SS |
|  |  | MRIA11 | 0.9702 | 1.011 | 0.8908 | 1.034 | SS |
|  |  | MRIA12 | 0.9622 | 2.115 | 0.8477 | 2.45 | SS |
|  |  | MRIA13 | 0.9628 | 1.036 | 0.7975 | 0.529 | SS |
|  |  | MR39 | 0.974 | 1.311 | 0.8126 | 0.4117 | SS |
|  |  | JA310 | 0.991 | 0.9768 | 0.9285 | 0.4895 | SS |
|  |  | MRLW5 | 0.9466 | 1.659 | 0.8597 | 1.678 | SS |
|  |  | MR24 | 0.9558 | 1.055 | 0.8076 | 1.091 | SS |
|  |  | MR26 | 0.9337 | 1.585 | 0.846 | 1.389 | SS |
|  |  | MR30 | 0.9637 | 0.3217 | 0.7715 | 1.978 | SS |
| 9 | Janus Kinase 2 (JAK2) JH1 domain | Gandotinib | 0.9957 | 0.7606 | 0.9315 | 0.7947 | SS |
|  |  | Pacritinib | 0.9892 | 0.3427 | 0.9177 | 0.2407 | SS |
|  |  | Lestaurtinib | 0.8222 | 1.461 | 0.7147 | 1.458 | SS |
|  |  | Cerdaulatinib | 0.9788 | 0.2864 | 0.5646 | 2.648 | SS |
|  |  | Ilginatinib | 0.8961 | 2.032 | 0.6161 | 2.433 | SS |
|  |  | Itacitinib | 0.9763 | 1.113 | 0.9257 | 1.101 | SS |
|  |  | Momelotinib | 0.9833 | 0.8511 | 0.9351 | 1.119 | SS |
|  |  | Ruxolitinib | 0.9746 | 1.687 | 0.6978 | 2.401 | SS |
|  |  | Fedratinib | 0.9839 | 2.331 | 0.8775 | 2.16 | SS |
|  |  | Baricitinib | 0.9689 | 1.91 | 0.8646 | 2.889 | SS |
|  |  | BL2-084 | 0.9852 | 1.428 | 0.9491 | 1.236 | SS |
|  |  | BL2-096 | 0.9683 | 1.661 | 0.8151 | 2.138 | SS |
|  |  | MA9-086 | 0.9653 | 1.414 | 0.6788 | 2.802 | SS |
|  |  | PN2-118 | 0.9595 | 1.722 | 0.6927 | 3.205 | SS |
|  |  | PN2-123 | 0.702 | 2.766 | 0.7423 | 2.729 | SS |
|  |  | PN3-115 | 0.9805 | 1.353 | 0.9179 | 1.045 | SS |
|  |  | PN4-014 | 0.9821 | 1.224 | 0.9651 | 1.272 | SS |
|  |  | PN4-073 | 0.8969 | 3.602 | 0.726 | 3.449 | SD |
|  |  | SG3-179 | 0.958 | 1.174 | 0.9253 | 1.624 | SS |
|  |  | SY5-103 | 0.9828 | 2.189 | 0.8715 | 2.041 | SS |
|  |  | BL2-057 | 0.9654 | 0.2985 | 0.9492 | 0.617 | SS |
|  |  | BL2-110 | 0.9359 | 0.8701 | 0.7647 | 1.193 | SS |
| 10 | HMG-CoA reductase (HMGR) | Mevastatin | 0.9614 | 0.8184 | 0.8326 | 1.308 | SS |
|  |  | Simvastatin | 0.9569 | 1.235 | 0.7423 | 1.343 | SS |
|  |  | Fluvastatin | 0.9053 | 0.7445 | 0.7739 | 1.566 | SS |
|  |  | Cerivastatin | 0.8279 | 1.542 | 0.726 | 1.717 | SS |
|  |  | Atorvastatin | 0.8876 | 2.511 | 0.6495 | 2.386 | SS |
|  |  | Rosuvastatin | 0.9723 | 1.561 | 0.8685 | 1.646 | SS |
|  |  | Compound 13 | 0.8841 | 1.681 | 0.7847 | 1.738 | SS |
|  |  | Compound 1 | 0.7788 | 2.541 | 0.3649 | 2.294 | SS |
|  |  | Compound 4 | 0.7909 | 1.966 | 0.7297 | 2.077 | SS |
|  |  | Compound 7 | 0.7677 | 3.177 | 0.67 | 3.475 | SD |
|  |  | Compound 9 | 0.9552 | 1.529 | 0.7787 | 1.628 | SS |
|  |  | Compound 20 | 0.9282 | 1.815 | 0.4973 | 3.069 | SS |
|  |  | Compound 15 | 0.995 | 1.572 | 0.8491 | 1.452 | SS |
|  |  | Compound 23 | 0.8911 | 1.367 | 0.7994 | 1.373 | SS |
|  |  | Compound 22 | 0.9675 | 1.121 | 0.9331 | 0.7883 | SS |

**Table S4.** Experimental binding affinities (*K*_d_) and corresponding R-values from HT-MD simulations. Experimental *K*_d_ values (reported in nM) were collected from the literature for each ligand in KRAS^G12D^, JAK2, HIV-1 protease, and HMGR.

| No | System | Ligand Name | Experimental *K*_d_ (nM) | R-value |
| --- | --- | --- | --- | --- |
| 1 | KRAS^G12D^ | MRTX1133 | 0.0002 | 0.9854 |
|  |  | Compound 15 | 0.8 | 0.9600 |
|  |  | Compound 5B | 3500 | 0.7299 |
|  |  | TH-Z816 | 25800 | 0.7488 |
|  |  | TH-Z827 | 6580 | 0.7184 |
| 2 | JAK2 | Gandotinib | 11 | 0.9833 |
|  |  | Pacritinib | 6.6 | 0.9732 |
|  |  | Lestaurtinib | 2.9 | 0.9986 |
|  |  | Cerdaulatinib | 4.8 | 0.9901 |
|  |  | Ilginatinib | 4.5 | 0.9976 |
|  |  | Itacitinib | 26 | 0.9227 |
|  |  | Momelotinib | 6.7 | 0.9835 |
|  |  | Ruxolitinib | 0.8 | 0.9722 |
|  |  | Fedratinib | 4.3 | 0.9736 |
| 3 | HIV-1pro | Saquinavir | 67.4 | 0.7436 |
|  |  | Indinavir | 1.07 | 0.7580 |
|  |  | Ritonavir | 0.6 | 0.8889 |
|  |  | Nelfinavir | 1.64 | 0.8386 |
|  |  | Amprenavir | 0.39 | 0.9844 |
|  |  | Lopinavir | 0.1 | 0.8213 |
|  |  | Atazanavir | 0.4 | 0.9525 |
|  |  | Tipranavir | 0.019 | 0.9519 |
|  |  | Darunavir | 0.0045 | 0.8981 |
| 4 | HMGR | Compound 13 | 27.4 | 0.8059 |
|  |  | Compound 1 | 13.5 | 0.8766 |
|  |  | Compound 4 | 51.7 | 0.7478 |
|  |  | Compound 9 | 12.7 | 0.8879 |
|  |  | Compound 20 | 14.3 | 0.9585 |
|  |  | Compound 15 | 26.3 | 0.9301 |
|  |  | Compound 23 | 95.7 | 0.8891 |
|  |  | Compound 22 | 21.6 | 0.8770 |

**Table S5**. Molecular structure of the modified TH-Z835 ligand, with the modification at the core heterocyclics, as well as at C2 and N7 positions and their HT-MD simulations results. All ligands are displayed here in their neutral forms (prior to protonation).

|  | | | | |
| --- | --- | --- | --- | --- |
| **Compound Name** | **R1** | **R2** | **Core** | **R-value** |
| TH-Z835_M1 |  |  |  | 0.8955 |
| TH-Z835_M2 |  |  |  | 0.9645 |
| TH-Z835_M3 |  |  |  | 0.9452 |
| TH-Z835_M4 |  |  |  | 0.9231 |
| TH-Z835_M5 |  |  |  | 0.4428 |
| TH-Z835_M6 |  |  |  | 0.7658 |
| TH-Z835_M7 |  |  |  | 0.8342 |
| **TH-Z835_M8** |  |  |  | 0.9720 |
| TH-Z835_M9 |  |  |  | 0.9596 |
| TH-Z835_M10 |  |  |  | 0.9424 |
| TH-Z835_M11 |  |  |  | 0.9365 |
| **TH-Z835_M12** |  |  |  | 0.9677 |
| TH-Z835_M13 |  |  |  | 0.9580 |
| TH-Z835_M14 |  |  |  | 0.8651 |
| TH-Z835_M15 |  |  |  | 0.8805 |
| TH-Z835_M16 |  |  |  | 0.9475 |
| TH-Z835_M17 |  |  |  | 0.9361 |
| TH-Z835_M18 |  |  |  | 0.9536 |
| TH-Z835_M19 |  |  |  | 0.9198 |
| TH-Z835_M20 |  |  |  | 0.6466 |
| **TH-Z835** |  |  |  | 0.8485 |

**Table S6**. Molecular structure of the modified MRTX1133 ligand, with the modification at C30 atom. Resulting in 15 ligands in processes and their HT-MD simulations results. All ligands are displayed here in their neutral forms (prior to protonation).

| **No** | **Compound Name** | **R-value** | **No** | **Compound Name** | **R-value** |
| --- | --- | --- | --- | --- | --- |
| **1** | **MRTX1133_M1** | 0.9907 | **9** | MRTX1133_M9 | 0.9824 |
| **2** | MRTX1133_M2 | 0.9617 | **10** | MRTX1133_M10 | 0.9751 |
| **3** | MRTX1133_M3 | 0.9708 | **11** | MRTX1133_M11 | 0.981 |
| **4** | MRTX1133_M4 | 0.9721 | **12** | **MRTX1133_M12** | 0.9918 |
| **5** | MRTX1133_M5 | 0.9412 | **13** | MRTX1133_M13 | 0.9815 |
| **6** | MRTX1133_M6 | 0.9589 | **14** | MRTX1133_M14 | 0.9795 |
| **7** | MRTX1133_M7 | 0.9788 | **15** | MRTX1133_M15 | 0.9477 |
| **8** | MRTX1133_M8 | 0.9813 | **16** | **MRTX1133** | 0.9854 |

**REFERENCES**

(1) Bekker, G. J.; Nagao, C.; Shirota, M.; Nakamura, T.; Katayama, T.; Kihara, D.; Kinoshita, K.; Kurisu, G. Protein Data Bank Japan: Computational Resources for Analysis of Protein Structures. *J. Mol. Biol.* **2025**, *437* (15). https://doi.org/10.1016/j.jmb.2025.169013.

(2) Bekker, G. J.; Ma, B.; Kamiya, N. Thermal Stability of Single-Domain Antibodies Estimated by Molecular Dynamics Simulations. *Protein Science* **2019**, *28* (2), 429–438. https://doi.org/10.1002/pro.3546.

(3) Bekker, G. J.; Araki, M.; Oshima, K.; Okuno, Y.; Kamiya, N. Dynamic Docking of a Medium-Sized Molecule to Its Receptor by Multicanonical MD Simulations. *Journal of Physical Chemistry B* **2019**, *123* (11), 2479–2490. https://doi.org/10.1021/acs.jpcb.8b12419.

(4) McGibbon, R. T.; Beauchamp, K. A.; Harrigan, M. P.; Klein, C.; Swails, J. M.; Hernández, C. X.; Schwantes, C. R.; Wang, L. P.; Lane, T. J.; Pande, V. S. MDTraj: A Modern Open Library for the Analysis of Molecular Dynamics Trajectories. *Biophys. J.* **2015**, *109* (8), 1528–1532. https://doi.org/10.1016/j.bpj.2015.08.015.

(5) Best, R. B.; Hummer, G.; Eaton, W. A. Native Contacts Determine Protein Folding Mechanisms in Atomistic Simulations. *Proc. Natl. Acad. Sci. U. S. A.* **2013**, *110* (44), 17874–17879. https://doi.org/10.1073/pnas.1311599110.

(6) Tan, B.; Zhang, X.; Ansari, A.; Jadhav, P.; Tan, H.; Li, K.; Chopra, A.; Ford, A.; Chi, X.; Ruiz, F. X.; Arnold, E.; Deng, X.; Wang, J. Design of a SARS-CoV-2 Papain-like Protease Inhibitor with Antiviral Efficacy in a Mouse Model. *Science (1979).* **2024**, *383* (6690), 1434–1440. https://doi.org/10.1126/science.adm9724.

(7) Garnsey, M. R.; Robinson, M. C.; Luong, †; Nguyen, T.; Cardin, R.; Tillotson, J.; Mashalidis, E.; Yu, A.; Aschenbrenner, L.; Balesano, A.; Behzadi, A.; Boras, B.; Chang, J. S.; Eng, H.; Ephron, A.; Foley, T.; Ford, K. K.; Frick, J. M.; Gibson, S.; Hao, L.; Hurst, B.; Kalgutkar, A. S.; Korczynska, M.; Lengyel-Zhand, Z.; Gao, L.; Meredith, H. R.; Patel, N. C.; Polivkova, J.; Rai, D.; Rose, C. R.; Rothan, H.; Sakata, S. K.; Vargo, T. R.; Qi, W.; Wu, H.; Liu, Y.; Yurgelonis, I.; Zhang, J.; Zhu, Y.; Zhang, L.; Lee, A. A. *Discovery of SARS-CoV-2 Papain-like Protease (PL pro ) Inhibitors with Efficacy in a Murine Infection Model*; 2024; Vol. 10. https://www.science.org.

(8) Jadhav, P.; Liang, X.; Ansari, A.; Tan, B.; Tan, H.; Li, K.; Chi, X.; Ford, A.; Ruiz, F. X.; Arnold, E.; Deng, X.; Wang, J. Design of Quinoline SARS-CoV-2 Papain-like Protease Inhibitors as Oral Antiviral Drug Candidates. *Nature Communications* **2025**, *16* (1). https://doi.org/10.1038/s41467-025-56902-x.

(9) Shen, Z.; Ratia, K.; Cooper, L.; Kong, D.; Lee, H.; Kwon, Y.; Li, Y.; Alqarni, S.; Huang, F.; Dubrovskyi, O.; Rong, L.; Thatcher, G. R. J.; Xiong, R. Design of SARS-CoV-2 PLpro Inhibitors for COVID-19 Antiviral Therapy Leveraging Binding Cooperativity. *J. Med. Chem.* **2022**, *65* (4), 2940–2955. https://doi.org/10.1021/acs.jmedchem.1c01307.

(10) Caputo, A. T.; Alonzi, D. S.; Marti, L.; Reca, I.-B.; Kiappes, J. L.; Struwe, W. B.; Cross, A.; Basu, S.; Lowe, E. D.; Darlot, B.; Santino, A.; Roversi, P.; Zitzmann, N. Structures of Mammalian ER α-Glucosidase II Capture the Binding Modes of Broad-Spectrum Iminosugar Antivirals. *Proceedings of the National Academy of Sciences* **2016**, *113* (32), E4630–E4638. https://doi.org/10.1073/pnas.1604463113.

(11) Karade, S. S.; Kolesnikov, A.; Treston, A. M.; Mariuzza, R. A. Identification of Endoplasmic Reticulum α-Glucosidase I from a Thermophilic Fungus as a Platform for Structure-Guided Antiviral Drug Design. *Biochemistry* **2022**. https://doi.org/10.1021/acs.biochem.2c00092.

(12) Karade, S. S.; Franco, E. J.; Rojas, A. C.; Hanrahan, K. C.; Kolesnikov, A.; Yu, W.; MacKerell, A. D.; Hill, D. C.; Weber, D. J.; Brown, A. N.; Treston, A. M.; Mariuzza, R. A. Structure-Based Design of Potent Iminosugar Inhibitors of Endoplasmic Reticulum α-Glucosidase I with Anti-SARS-CoV-2 Activity. *J. Med. Chem.* **2023**, *66* (4), 2744–2760. https://doi.org/10.1021/acs.jmedchem.2c01750.
